# Pharmacovigilance of SSRIs in Children and Adolescents with Depression: Disproportionality Analysis of Serotonin Adverse Events Using the FAERS Database

**DOI:** 10.2174/0109298673440558260406211258

**Published:** 2026-05-22

**Authors:** Hongyi Du, Jiabei Liu, Ruihua Zhou, Chenghui Kuang, Ke Ma

**Affiliations:** 1 College of Traditional Chinese Medicine, Shandong University of Traditional Chinese Medicine, Jinan, 250355, P.R. China;; 2 College of Acupuncture and Massage, Shandong University of Traditional Chinese Medicine, Jinan, 250355, P.R. China;; 3 Shandong Co-Innovation Center of Classic TCM Formula, Shandong University of Traditional Chinese Medicine, Jinan, 250355, P.R. China

**Keywords:** SSRIs, fluoxetine, sertraline, escitalopram, fluvoxamine, minors, depression, pharmacovigilance, FAERS, adverse drug reactions

## Abstract

**Introduction:**

Depression in minors is a growing global health concern with limited pharmacological options. This study evaluated the safety profiles of four selective serotonin reuptake inhibitors (SSRIs)-fluoxetine, sertraline, escitalopram, and fluvoxamine-in children and adolescents, focusing on serious adverse events (SAEs).

**Methods:**

Pediatric adverse event reports from the FAERS database (Q1 2004-Q1 2025) were analyzed. Only reports listing the study drugs as Primary Suspect were included; duplicates were removed. Events were coded using MedDRA 26.1 and analyzed at system organ class (SOC) and preferred term (PT) levels. Disproportionality analyses used reporting odds ratio (ROR), proportional reporting ratio (PRR), Bayesian confidence propagation neural network (BCPNN), and multi-item gamma-Poisson shrinker (MGPS), with Bayesian shrinkage (EBGM05) applied to low-frequency signals.

**Results:**

Psychiatric events, including suicidal ideation, suicide attempts, and self-injury, showed the most consistent signals. Fluoxetine and sertraline had broad profiles; escitalopram showed higher disproportionality for psychiatric, cardiac, and perinatal events; fluvoxamine showed elevated signals for impulsive behaviors and rare systemic events. Neurological events, such as serotonin syndrome and tremor, were consistently reported; congenital and perinatal signals had limited statistical stability.

**Discussion:**

SSRIs share common psychiatric AE signals in minors, but differences in strength and distribution likely reflect real-world exposure, metabolic pathways, and structural properties rather than causal differences.

**Conclusion:**

FAERS-based pharmacovigilance provides a comparative overview of SSRI safety, with multi-algorithm assessment and Bayesian shrinkage enhancing low-frequency signal reliability, informing safer SSRI use and guiding future studies integrating pharmacokinetics and pharmacogenomics.

## 1 INTRODUCTION

Depression is a globally prevalent mental disorder characterized by persistent low mood and anhedonia, often accompanied by impaired concentration, loss of interest, and suicidal ideation. It has profound impacts on individuals, families, and society, and is one of the leading causes of health-related disability and global disease burden [1]. Approximately 4.7% of the population experiences a depressive episode within one year, and in minors, the prevalence increases sharply during adolescence, with nearly one-quarter of adolescents having experienced a depressive episode by the age of 19 [2]. Depression in minors is closely associated with interpersonal difficulties, impaired academic and occupational performance, and reduced quality of life; it also increases the risk of multiple physical and mental illnesses, self-harm, and suicide [3, 4]. However, resources for the prevention and treatment of adolescent depression remain severely inadequate worldwide. Even in high-income countries, fewer than 20% of patients with major depression receive effective treatment, while in low- and middle-income countries, the proportion is even lower, at less than 10% [5]. Amid economic transition and increasing social pressure, the prevalence of adolescent depression continues to rise, underscoring the need for more precise screening, prevention, and intervention strategies based on epidemiological evidence [6, 7].

In terms of pharmacological treatment, selective serotonin reuptake inhibitors (SSRIs) are currently the most commonly prescribed first-line antidepressants for depressive disorders in children and adolescents, owing to their relatively well-established efficacy and overall favorable tolerability, and are therefore widely used in clinical practice. Compared with tricyclic antidepressants or certain noradrenergic agents, SSRIs generally exhibit lower toxicity in overdose, reduced anticholinergic burden, and a lower risk of serious cardiac arrhythmias, which contributes to their preferential use in pediatric populations [8]. Nevertheless, evidence from previous meta-analyses and randomized controlled trials indicates that the use of SSRIs in pediatric populations has been associated with an increased risk of suicidality, particularly during early treatment or in susceptible individuals [9-11]. Moreover, most evidence regarding antidepressant safety has been derived from adult populations. Given the substantial differences between adolescents and adults in terms of neurodevelopment, physiological maturation, and drug metabolism, adverse reaction profiles observed in adults may not be directly generalizable to younger individuals [12-14]. Previous studies have also shown that, compared with placebo, SSRIs treatment in pediatric patients is more frequently associated with adverse events such as agitation, abdominal pain, and sedation or somnolence [15].

Based on these considerations, the present study selected fluoxetine, escitalopram, sertraline, and fluvoxamine as the drugs of interest for safety analysis. This selection was guided by the following factors: (1) all four SSRIs are widely used in the treatment of psychiatric disorders among minors and thus demonstrate good real-world representativeness; and (2) fluoxetine and escitalopram have been approved by regulatory authorities for the treatment of depressive disorders in minors, whereas sertraline and fluvoxamine are primarily approved for obsessive-compulsive disorder or anxiety-related disorders in pediatric populations. However, in routine clinical practice, the latter two agents are also commonly prescribed to minors with depression accompanied by prominent anxiety symptoms, thereby reflecting real-world prescribing patterns [16, 17].

Using data from the U.S. Food and Drug Administration Adverse Event Reporting System (FAERS), this study conducted a comparative analysis of serious adverse events associated with these four SSRIs in minors. Through a systematic pharmacovigilance evaluation, we aimed to further characterize the safety profiles of individual SSRIs and to provide more robust evidence to inform clinical decision-making. We anticipate that the findings of this study may offer useful insights for pharmacological treatment of depression in minors, contributing to more informed prescribing decisions and improved safety monitoring in pediatric antidepressant therapy. In addition, this analysis may enhance the understanding of antidepressant-related adverse reactions and support more effective clinical management and risk control in antidepressant therapy for minors.

## 2 METHODS

### 2.1 Data Source

Data for this study were obtained from the FAERS datasets, focusing on adverse event reports associated with fluoxetine, escitalopram, sertraline, and fluvoxamine. We downloaded quarterly data files from Q1 2004 to Q1 2025 from the FAERS official website (https://open.fda.gov/data/faers/) in ASCII text format. The downloaded datasets included key fields such as patient demographics (DEMO), drug records (DRUG), adverse event outcomes (OUTCOME), reporting sources (REPORTER), indications (INDICATIONS), and therapy duration (THERAPY). Data preprocessing and subsequent analyses were conducted using R (version 4.5.2).

### 2.2 Data Processing and Analysis Methods

First, duplicate records in the FAERS raw data were removed: when identical Case IDs were found in the DEMO table, only the most recent report date was retained. Drug names were standardized using the Medex_UIMA_1.8.3 tool, and reports in which fluoxetine, escitalopram, sertraline, or fluvoxamine was designated as the primary suspect drug were extracted. Variables obtained included report time, patient sex, age, reporting source, and country. The drug dictionaries are provided in Supplementary Tables **S1**-**S4**.

For signal detection, we applied four methods: reporting odds ratio (ROR) [18], proportional reporting ratio (PRR) [19], Bayesian confidence propagation neural network (BCPNN) [20], and multi-item gamma-Poisson shrinker (MGPS) [21]. ROR and PRR are based on 2×2 contingency table frequency analysis: ROR assesses the strength of association between a drug and a specific adverse event by calculating the reporting ratio, offering simple and intuitive results but being sensitive to rare events and small samples [22]. PRR measures the proportion of a specific drug-event pair among all reports compared with the background proportion, performing better in large datasets but still prone to reporting bias [18, 23]. In contrast, BCPNN applies a Bayesian statistical framework, calculating the Information Component (IC) and its confidence interval to assess drug-event associations, providing robust estimates in sparse data and effectively controlling false positives. MGPS applies gamma-Poisson shrinkage to adjust observed-to-expected (O/E) ratios and generates the empirical Bayes geometric mean (EBGM) with corresponding confidence intervals, improving stability for multiple comparisons and low-frequency signals [24]. This O/E-based shrinkage estimation effectively mitigates the inflation of signal strength caused by low reporting frequency or small sample sizes, thereby providing improved stability and robustness when addressing multiple comparisons and rare adverse events. Accordingly, when the lower bound of the EBGM confidence interval (EB05) exceeds 1, the drug-event association is generally considered to be stable and reliable within the Bayesian framework.

Therefore, in practical pharmacovigilance research, the combined application of four complementary signal detection methods-ROR, PRR, BCPNN, and MGPS-enables cross-validation of detected signals, improving both sensitivity and specificity while reducing the risk of false-positive findings attributable to the limitations of any single algorithm. In the present study, a drug-adverse event signal was considered statistically significant only when all of the following criteria were satisfied: a minimum of three reports (a ≥ 3), an ROR or PRR ≥ 2.0, and a lower bound of the 95% confidence interval (95% CI) greater than 1.0. Results derived from the MGPS analysis were primarily used to assess the stability and consistency of detected signals rather than serving as an independent primary decision criterion. Detailed descriptions of each algorithm and the corresponding decision rules are provided in Supplementary Table **S5** [25, 26]. Detailed methodological information is provided in Table **1**.

It should be noted that disproportionality analyses are designed for signal detection rather than causal inference, and the identified associations do not imply causality.

### 2.3 Study Size and Rationale

This study was based on the FAERS dataset and included all reports that met the predefined criteria, namely those in which fluoxetine, sertraline, escitalopram, or fluvoxamine was listed as the primary suspect drug, the patient was younger than 18 years of age, and duplicate cases were removed using case identification numbers. Because disproportionality analysis methods (including ROR, PRR, BCPNN, and MGPS) are non-parametric signal detection approaches based on 2×2 contingency tables, a priori sample size estimation was not required. The inclusion of all eligible reports was intended to maximize the use of available information, thereby improving the stability of signal estimation and reducing potential selection bias. The statistical frameworks, signal metrics, and interpretation criteria for each disproportionality method applied in this study are summarized in Supplementary Table **S5**.

### 2.4 Signal Filtering and Classification

To improve the stability and reliability of the analysis, only Preferred Terms (PTs) with at least three reports were included for subsequent evaluation. All adverse events were coded and classified according to MedDRA version 26.1 at both the PT and System Organ Class (SOC) levels, allowing identification of the major system-level distribution of signals.

## 3 RESULTS

### 3.1 Annual Reports of Adverse Events

Fig. (**1**) illustrates the temporal trends and proportional distribution of adverse event reports associated with four selective serotonin reuptake inhibitors (SSRIs)-fluoxetine, sertraline, escitalopram, and fluvoxamine-in minors reported to the FAERS database from 2004 to 2025. The upper panel depicts the annual number of adverse event reports for each drug over the study period. Overall, the four SSRIs exhibited distinct temporal patterns, with noticeable differences in reporting trends over time. Throughout the entire period, sertraline and fluoxetine accounted for relatively higher numbers of reports, with a marked increase observed after 2010; sertraline reached a peak during 2018-2019, whereas fluoxetine showed a transient peak around 2015. In contrast, escitalopram was associated with a moderate number of reports, characterized by a gradual increase followed by stabilization, while fluvoxamine consistently showed low reporting volumes with only minor fluctuations across the study period. Additional details are presented in Fig. (**1**).

### 3.2 General Characteristics of Adverse Event Reports

Based on FAERS data from 2004 to 2025, this study systematically analyzed adverse event reports associated with fluoxetine, sertraline, escitalopram, and fluvoxamine in minors, with a focus on general characteristics including demographic features, indications, reporting sources, clinical outcomes, and temporal patterns. Overall, the four SSRIs exhibited notable differences in reporting patterns among pediatric populations (Table **2**). Fluoxetine and fluvoxamine showed relatively higher proportions of reports among adolescents aged 12-18 years, suggesting more frequent use in this age group, whereas sertraline and escitalopram were more commonly reported in children younger than 6 years, which may reflect differences in prescribing preferences across developmental stages. Regarding reporting sources, adverse event reports for all four drugs were predominantly submitted by physicians, followed by other healthcare professionals. In terms of clinical outcomes, after excluding reports with unknown seriousness, hospitalization represented the most frequently reported outcome across all four SSRIs. Geographically, the majority of reports originated from high-income countries, particularly the United States. With respect to routes of administration, adverse event reports involving minors predominantly indicated oral administration or other unspecified/non-standard routes across all four SSRIs. For fluoxetine, sertraline, and fluvoxamine, oral administration accounted for a substantial proportion of reports (each exceeding approximately 40%), consistent with their routine use *via* conventional oral dosing in clinical practice. Across all four SSRIs, a slightly higher proportion of reports involved female patients. Female cases accounted for the majority of reports for fluoxetine, sertraline, and escitalopram (approximately 54%-58%), whereas fluvoxamine-related reports showed a relatively higher proportion of males compared with females, indicating potential differences in sex distribution among the drugs. The proportion of missing sex information was generally low, suggesting relatively complete recording of this variable in the FAERS database. In terms of time to onset (TTO), all four drugs demonstrated a tendency toward early reporting of adverse events, with most reports occurring within 7 days of drug initiation, followed by a secondary peak of delayed events occurring at ≥60 days. This pattern suggests that SSRI-associated adverse events may be reported both shortly after treatment initiation and after a period of continued exposure. Sertraline, escitalopram, and fluvoxamine showed relatively higher proportions of reports within the first 7 days, whereas escitalopram also exhibited a comparatively higher proportion of reports occurring at ≥60 days. A certain proportion of reports lacked TTO information, which is consistent with the inherent characteristics of spontaneous reporting systems. Body weight (WT) data indicated that the median reported weights for fluoxetine-, sertraline-, and fluvoxamine-related cases fell within ranges commonly observed in adolescent populations, whereas escitalopram-related reports showed a notably lower median body weight, suggesting that these reports may include younger or lighter minors. The interquartile ranges of body weight were wide across all four drugs, reflecting substantial heterogeneity in age and physiological development within the pediatric population.

### 3.3 Indications of the Four SSRIs

A comprehensive analysis of the indication distributions associated with adverse event reports for the SSRIs in the FAERS database revealed both shared patterns and distinct inter-drug differences in prescribing contexts among minors (Figs. **2**-**4**). For clarity and interpretability, only indications reported more than four times are displayed in Figs. (**2****-****4**). Overall, depressive disorders and anxiety disorders constituted the most frequently reported indications across all four drugs, reflecting their core clinical roles as antidepressant and anxiolytic agents. Among the four SSRIs, fluoxetine and sertraline accounted for the largest number of adverse event reports and exhibited the broadest range of reported indications. In addition to depression and anxiety, these reports encompassed bipolar disorder, post- traumatic stress disorder, attention-deficit/hyperactivity disorder, autism spectrum disorder, and various psychiatric and behavioral abnormalities, suggesting more diverse real-world utilization scenarios. In contrast, escitalopram-related adverse event reports showed a more concentrated indication profile, predominantly involving depression and anxiety disorders. Notably, escitalopram reports also included a relatively higher proportion of pregnancy- and perinatal-related indications, indicating its use within specific clinical contexts. Fluvoxamine was associated with the lowest overall number of adverse event reports, and its indication profile was highly concentrated in obsessive-compulsive disorder and anxiety-related conditions. The proportion of reports involving depression was substantially lower than that observed for the other three SSRIs, a pattern consistent with its primary clinical positioning as a treatment for obsessive-compulsive disorder rather than as a first-line antidepressant in minors. Additionally, all four drugs included a non-negligible proportion of reports with unspecified or unclear indications, highlighting the presence of incomplete medication records or off-label use within real-world data. Taken together, these inter-drug differences in indication distributions provide important contextual information for interpreting adverse event profiles and safety signals, and underscore the importance of individualized medication assessment and risk management in pediatric populations.

### 3.4 SOC-level Disproportionality Analysis of Adverse Events

Tables **3-6** present the SOC-level disproportionality analyses of adverse event reports associated with fluoxetine, sertraline, escitalopram, and fluvoxamine in the treatment of depression among minors. Multiple signal detection methods-including the ROR, PRR, BCPNN, and MGPS-were jointly applied to comprehensively assess the magnitude and stability of drug-event reporting imbalances. Overall, results derived from different algorithms showed a high degree of concordance at the SOC level, supporting the internal consistency of the findings. Across all four SSRIs, psychiatric disorders consistently represented the most frequently reported SOC and exhibited the most robust and stable disproportionality patterns across both frequentist and Bayesian approaches. This observation is consistent with the central nervous system-targeted pharmacological profiles of SSRIs and reflects the predominance of psychiatric events in pediatric adverse event reporting. Among non-psychiatric SOCs, congenital, familial, and genetic disorders showed elevated reporting imbalances for fluoxetine, sertraline, and escitalopram, with concordant patterns observed across multiple algorithms, indicating stable reporting associations at the SOC level. In contrast, fluvoxamine was associated with a limited number of reports in these categories; although point estimates exceeded unity in some analyses, the corresponding confidence intervals were wide, suggesting limited stability and warranting cautious interpretation. In addition, escitalopram exhibited relatively higher disproportionality estimates in the SOC of pregnancy, puerperium, and perinatal conditions. With respect to cardiac disorders, fluoxetine, sertraline, and escitalopram showed reporting imbalances supported primarily by chi-square statistics and IC (IC025) estimates, whereas fluvoxamine did not display comparable positive signals for cardiac-related SOCs under standard analytical thresholds. In the SOC of nervous system disorders, all four drugs demonstrated positive estimates in chi-square and IC (IC025) analyses; however, for fluvoxamine, Bayesian shrinkage estimates suggested limited signal stability. For most other SOCs-including gastrointestinal disorders, skin and subcutaneous tissue disorders, metabolism and nutrition disorders, infections and infestations, blood and lymphatic system disorders, immune system disorders, hepatobiliary disorders, vascular disorders, and renal and urinary disorders-disproportionality estimates across all four drugs were generally below unity across multiple detection methods. These patterns indicate an absence of relative enrichment of adverse event reporting in these systems, with Bayesian shrinkage analyses further supporting the stability of these observations. In summary, while SSRIs shared a common pattern of reporting enrichment within psychiatric SOCs, notable inter-drug differences were observed across several non-psychiatric organ systems. These findings provide an important foundation for subsequent analyses at more granular levels (*e.g.*, preferred term-level analyses) and for mechanistic interpretation in conjunction with drug-specific pharmacological and pharmacokinetic characteristics.

### 3.5 PT-level Analysis of Adverse Events

Tables **7**-**10** summarize the top 20 adverse events at the PT level, ranked by reporting frequency, associated with fluoxetine, sertraline, escitalopram, and fluvoxamine in minors treated for depression. Overall, PT-level signals were highly clustered rather than evenly distributed, with concentrations observed in categories related to suicidal and self-injurious behaviors, pregnancy- and perinatal-related outcomes, congenital structural abnormalities, central nervous system responses, and cardiac electrophysiological findings. Across all four SSRIs, results derived from the ROR, PRR, BCPNN, and MGPS were largely concordant, supporting the internal consistency of the detected reporting patterns. For fluoxetine, the most frequently reported PTs were predominantly related to suicidal behaviors, including suicidal ideation, intentional self-injury, suicide attempt, and completed suicide, all of which consistently ranked among the leading PTs. Other psychiatric PTs, such as agitation, confusional state, depressed mood, and drug abuse, were also commonly reported. Among non-psychiatric events, intentional overdose was a prominent PT within the injury and poisoning category. Several PTs related to pregnancy and fetal exposure showed consistent over-representation in fluoxetine-associated reports, indicating a concentration of perinatal exposure-related records. Notably, serotonin syndrome represented one of the strongest PT-level signals overall, followed by tremor and syncope. In addition, electrocardiographic QT interval prolongation and urinary retention were among the frequently reported PTs, and multiple congenital cardiac anomalies-including atrial septal defect, patent ductus arteriosus, and ventricular septal defect-were recurrently observed, suggesting potential relevance to prenatal or perinatal exposure contexts. For sertraline, the PT-level profile was characterized by the coexistence of severe psychiatric events and a broad range of pregnancy- and neonatal-related outcomes. Intentional self-injury, suicide attempt, suicidal ideation, and completed suicide were among the most frequently reported PTs, with intentional self-injury ranking particularly high. Neurological PTs included tremor and serotonin syndrome, while neonatal agitation showed notable concentration, highlighting perinatal exposure-related reporting patterns. Multiple pregnancy-related PTs, such as fetal exposure during pregnancy and maternal exposure with unknown timing, were repeatedly observed and were accompanied by neonatal outcomes, including small for gestational age and neonatal respiratory disorders. Congenital cardiac anomalies-such as atrial septal defect, ventricular septal defect, and patent ductus arteriosus-were consistently represented, indicating a recurring pattern of structural cardiac findings in sertraline-associated reports. Escitalopram exhibited a more focused but comparatively intense PT-level profile, with several adverse events ranking among the highest across all analyzed drugs. Pregnancy- and perinatal-related outcomes were prominent, including fetal exposure during pregnancy, small for gestational age, preterm birth, neonatal respiratory disorders, and neonatal drug withdrawal syndrome. Congenital and developmental anomalies constituted another major category, with PTs such as atrial septal defect, ventricular septal defect, patent ductus arteriosus, congenital malformations, clubfoot, and craniosynostosis appearing among the most frequently reported. Severe psychiatric outcomes-including suicide attempt, suicidal ideation, completed suicide, and intentional self-injury-were also commonly reported and ranked higher relative to fluoxetine and sertraline. In addition, tremor and QT interval prolongation were present among the leading PTs, drawing attention to central nervous system excitability and cardiac electrophysiological considerations. In contrast, fluvoxamine was associated with a smaller overall number of reports, but its PT-level signals were highly concentrated and pronounced. Suicide attempt was the most frequently reported event, followed by intentional overdose, agitation, suicidal ideation, intentional self-injury, and completed suicide. Of particular note were PTs related to disinhibition and impulsive behaviors, which showed exceptionally high disproportionality estimates, indicating a strong concentration of such reports among minors. Neurological PTs included somnolence, serotonin syndrome, and papilledema; although the latter was based on a limited number of cases, it ranked highly among reported PTs. Several severe non-psychiatric events-including rhabdomyolysis, metabolic acidosis, QT interval prolongation, and elevated liver enzymes-were also among the leading PTs. Neonatal drug withdrawal syndrome appeared among the major signals as well, suggesting potential perinatal safety considerations; however, given the small number of reports, these findings warrant cautious interpretation.

## 4 DISCUSSION

### 4.1 Study Positioning and Methodological Considerations

It should be emphasized that the present study was not designed to estimate the true incidence or to establish causal relationships between SSRIs and adverse events in minors. Instead, using a real-world spontaneous reporting system, this analysis aimed to systematically compare both shared and drug-specific patterns within the safety signal profiles of different SSRIs. Although a pharmacovigilance framework was applied, the scientific value of this work extends beyond descriptive pharmacoepidemiology. Specifically, it lies in contextualizing observed adverse event signals alongside differences in molecular structure, pharmacokinetic properties, metabolic pathways, and cytochrome P450 inhibition profiles among individual SSRIs, thereby providing a hypothesis-generating basis for potential drug-specific safety mechanisms. SSRIs differ substantially in lipophilicity, central nervous system distribution, elimination half-life, presence of active metabolites, and the strength of inhibition across CYP450 enzyme systems. These pharmacochemical and pharmacokinetic characteristics may influence the duration of systemic and central exposure, susceptibility to drug-drug interactions, and the manifestation patterns of central and peripheral toxicities. Consequently, interpretation of signal heterogeneity observed in the FAERS database should be grounded in these pharmacological and metabolic contexts, rather than being viewed simply as differences in clinical risk across agents.

### 4.2 Psychiatric Adverse Events

Among minors with depressive disorders, psychiatric adverse events remain the most frequently reported and clinically relevant safety concerns associated with SSRI therapy. These events primarily include suicidal ideation, suicide attempts, completed suicide, intentional self-harm, agitation, impulsive behavior, and emotional dysregulation. Although increases in suicidal ideation have been reported during antidepressant treatment, population-level evidence suggests that completed suicide rates have shown a declining trend in parallel with increased antidepressant prescribing [27-29]. In the present study, the above outcomes were collectively classified as “suicide-related adverse events” for analysis. Evaluation of FAERS data indicated that all four SSRIs examined-fluoxetine, sertraline, escitalopram, and fluvoxamine-exhibited consistent disproportionality signals for suicide-related events in minors, suggesting that such reports are commonly captured during pediatric SSRI use. According to Hetrick *et al.* (2021), while newer generation antidepressants such as fluoxetine and escitalopram demonstrate efficacy in alleviating depressive symptoms, the study also highlights that these medications may pose an increased risk of suicide-related behaviors in the adolescent population, particularly during the early stages of treatment when the risk of suicidal ideation and behaviors is elevated [30].At the same time, noticeable differences were observed across agents with respect to reporting frequency and signal strength. Suicide-related adverse events in adolescent depression are influenced by a complex interplay of biological, psychological, and social factors [31]. From a neurodevelopmental perspective, immaturity of prefrontal cortical regulatory circuits combined with heightened reactivity of the amygdala and limbic systems renders adolescents physiologically vulnerable to emotional dysregulation and impaired impulse control. Within this context, early SSRI treatment-through enhancement of central serotonergic neurotransmission-may coincide with a so-called activation period, during which behavioral disinhibition may become more apparent and pre-existing suicidal ideation may be more readily expressed as behavior [32, 33]. This phenomenon has been described as a temporal mismatch whereby improvements in energy and motivation precede mood stabilization, potentially facilitating behavioral expression of suicidal thoughts [34]. Recent studies suggest that intestinal epithelial serotonin (5-HT) plays a key role in regulating mood and anxiety, particularly through gut-brain communication. Hung *et al.* (2025) found that SSRIs affect mood *via* serotonin signaling not only in the central nervous system but also in the gut, providing new insights into the multi-system effects of SSRIs in treating mood disorders [35]. Psychologically, adolescents are prone to affective lability and catastrophic thinking, while insufficient family support, academic stress, peer difficulties, or exposure to online suicide-related content may further amplify emotional fluctuations observed during SSRI treatment [36, 37]. The present analysis suggests heterogeneity across individual SSRIs with respect to suicide-related reporting patterns. Regulatory authorities, including the U.S. Food and Drug Administration (FDA), have required boxed warnings for all SSRIs highlighting the potential for increased suicidality in children, adolescents, and young adults under 24 years of age [9]. In FAERS reports, sertraline accounted for the largest number of suicide-related submissions, a pattern that may reflect its widespread use in community and primary care settings, where systematic monitoring may be less intensive. Sertraline is frequently prescribed for depressive disorders accompanied by prominent anxiety symptoms, a clinical profile often characterized by emotional instability and impulsivity, which may overlap with features of the activation syndrome [38]. Nevertheless, clinical trials have generally reported favorable tolerability and low discontinuation rates for sertraline, supporting its use in patients who have not tolerated other treatments [39]. Compared with sertraline, fluoxetine showed a slightly lower number of reports but a comparatively higher signal intensity. Previous pediatric trials, including those reported by Emslie and colleagues, have noted increases in suicidal ideation during early phases of fluoxetine treatment in some patients [40]. A network meta-analysis published in The Lancet, which included 5,260 patients aged 6-18 years, identified fluoxetine as the only antidepressant demonstrating efficacy superior to placebo, with favorable rankings for both efficacy (SUCRA 76.6%) and tolerability (SUCRA 75.7%) [16]. Long-term follow-up studies have further suggested reduced relapse during maintenance treatment, supporting its continued use in pediatric depression. As the only SSRI approved by the FDA for patients aged 8 years and older, fluoxetine is commonly prescribed in younger children with limited emotional regulation capacity [41]. Its long elimination half-life (2-4 days) and the presence of an active metabolite, norfluoxetine (7-15 days), may prolong serotonergic exposure, which could coincide with sustained periods of agitation or impulsivity during treatment initiation. Consequently, international guidelines generally recommend fluoxetine as a first-line option for pediatric depression, while emphasizing close monitoring in individuals with elevated suicidality vulnerability [42, 43]. A comprehensive review of treatment strategies for adolescent depression reveals that the combination of fluoxetine and cognitive behavioral therapy (CBT) yields the best treatment response, while fluoxetine alone also demonstrates significant efficacy. In contrast, CBT alone showed no significant difference when compared to a placebo [44, 45]. This combined approach of pharmacological treatment and psychosocial interventions has shown higher efficacy and acceptability in treating various pediatric and adolescent psychiatric disorders [46]. Escitalopram was associated with a smaller overall number of reports but showed concentrated signals for severe outcomes such as suicide attempts and intentional overdose 47. FAERS data indicate consistent reporting of associations with suicidal ideation and self-harm, some of which are not explicitly detailed in product labeling [47-49]. In clinical practice, escitalopram is often prescribed for severe, recurrent, or psychotic depression-populations characterized by higher baseline vulnerability to suicidality [50]. As such, its use may be more representative of high-risk clinical contexts rather than intrinsic drug effects. Careful dose adjustment, integration of psychosocial interventions, and close monitoring are therefore recommended when escitalopram is used in pediatric populations [51]. Fluvoxamine accounted for the fewest reports overall, although selected signals appeared relatively elevated. Its primary approved indications involve anxiety and obsessive-compulsive disorder, with more limited direct evidence for pediatric depression [52, 53]. In clinical practice, its use for adolescent depression is generally restricted to cases with comorbid obsessive-compulsive symptoms and is not typically recommended as a first-line SSRI [54]. To further contextualize heterogeneity in psychiatric adverse event signals across SSRIs, we integrated drug-specific pharmacochemical and pharmacokinetic characteristics (Table **11**). Fluoxetine is characterized by high lipophilicity and strong brain penetration, along with prolonged half-lives of both the parent compound and its active metabolite, which may extend central serotonergic exposure and coincide with persistent activation-related signals [55]. In addition, potent inhibition of CYP2D6 may increase the likelihood of drug-drug interactions, potentially influencing central adverse event reporting patterns [56, 57]. Sertraline, while also exhibiting substantial brain penetration, has a shorter half-life and lacks active metabolites, which may correspond to broader but less extreme psychiatric signal distributions [58]. Escitalopram’s dependence on CYP2C19 metabolism introduces interindividual variability in exposure, particularly given the prevalence of CYP2C19 polymorphisms in pediatric populations, which may contribute to disproportionate reporting in susceptible subgroups [59]. Fluvoxamine’s strong CYP1A2 inhibition and σ1 receptor affinity further distinguish its pharmacological profile and may provide a plausible framework for interpreting clustered impulsivity-related signals [60]. It is important to emphasize that these pharmacological considerations are intended to facilitate interpretation of observed safety signals rather than to support causal inference. Psychiatric adverse event reports captured in FAERS are more likely to reflect the combined influence of real-world drug exposure, patient vulnerability, comorbidities, concomitant medications, and reporting behavior, rather than direct drug-induced effects.

### 4.3 Nervous System-related Events

Among nervous system AEs, serotonin syndrome and tremor were the two clinically relevant findings observed in this study. Reports involving fluoxetine and sertraline were more common, whereas those involving escitalopram and fluvoxamine were comparatively fewer. This pattern is consistent with existing prescribing practices, as fluoxetine and sertraline are recommended by multiple international guidelines as first-line SSRIs for adolescent depression and are widely used in clinical settings [61]. Although serotonin syndrome is uncommon in minors, reported cases typically involve autonomic instability, altered mental status, and neuromuscular abnormalities, and have been described more frequently in the context of rapid dose escalation or concomitant use of other serotonergic agents [62, 63]. In this context, the distribution of serotonin syndrome reports in FAERS may reflect differential exposure across commonly used SSRIs rather than drug-specific susceptibility. These observations highlight the need for careful monitoring during dose adjustments and polypharmacy in adolescent patients.

Tremor appeared more frequently among reports involving sertraline and escitalopram than among those involving fluoxetine or fluvoxamine. Tremor is a relatively common AE reported with SSRI use, and its occurrence in adolescents treated with sertraline or escitalopram has been described in previous studies [48]. While not life-threatening, tremors may interfere with daily functioning and treatment adherence in adolescents. Persistent motor symptoms may contribute to dose modification or treatment discontinuation, potentially affecting overall therapeutic outcomes [64]. As summarized in Table **11**, SSRIs with higher lipophilicity and greater brain penetrability are more likely to achieve higher effective exposure within the central nervous system60.In parallel, differences in CYP inhibition profiles across individual agents may indirectly modulate overall serotonergic tone through drug-drug interactions [65]. These pharmacological characteristics provide a plausible background for interpreting the distribution of nervous system-related adverse event signals; however, they are insufficient to distinguish exposure-driven effects from potential drug-specific risk.

### 4.4 Metabolic and Systemic Reactions

In this study, several notable signals were observed in metabolic and systemic AEs. For fluvoxamine, strong associations were detected with rhabdomyolysis and metabolic acidosis. Although the absolute number of reports was small, the strength of the disproportionality signals suggests that these events, while rare, may represent severe idiosyncratic reactions in susceptible minors. Case reports have previously described fluvoxamine-induced rhabdomyolysis, often linked to drug interactions or overdose, which can rapidly progress to renal impairment if not promptly recognized and treated. Previous case reports have described instances of rhabdomyolysis reported in association with fluvoxamine exposure, often in the context of drug-drug interactions or overdose. These reports provide biological plausibility for the observed signals but do not establish causality. Taken together, the FAERS findings highlight the need for further evaluation of these rare events in independent data sources and clinical settings [66]. As shown in Table **11**, the strong inhibitory effect of fluvoxamine on CYP1A2, together with its σ1 receptor-related central regulatory properties, may, under specific treatment contexts, influence energy metabolism and systemic homeostasis, thereby offering a hypothesis-generating explanation for these rare but severe adverse event signals [60, 67]. However, given the limited number of reported cases and the pronounced Bayesian shrinkage observed in EBGM05 estimates, these findings should be interpreted as safety signals requiring further validation rather than as evidence of stable or established risk [68]. For escitalopram, this study detected a disproportionality signal for metabolic acidosis. External evidence suggests that antidepressant exposure is more commonly associated with metabolic acidosis in the context of polydrug intoxication or overdose. Clinically, when acid-base imbalance is suspected, arterial blood gas analysis (ABG; evaluating pH, PaCO_2_, HCO_3_^−^, and anion gap), together with serotonin lactate measurement, should be performed promptly to distinguish high- *versus* normal-anion gap metabolic acidosis. At the same time, potential concomitant medications and complicating factors (*e.g.*, seizures, hypoperfusion, or shock) should be systematically investigated [69]. From a mechanistic perspective, recent mitochondrial research has suggested that certain psychotropic medications may impair energy metabolism. A systematic review reported: “Only amitriptyline, escitalopram, and haloperidol were identified as having exclusively adverse effects on mitochondrial function.” This finding provides a plausible mechanistic hypothesis of “mitochondrial dysfunction,” but the evidence remains indirect and requires further clinical studies to establish a causal link with metabolic acidosis [70].

### 4.5 Cautious Interpretation of Perinatal and Congenital Signals

Signals observed at the SOC level for Congenital, familial and genetic disorders and Pregnancy, puerperium, and perinatal conditions, particularly those involving escitalopram and fluvoxamine, warrant careful interpretation. Recent studies show that perinatal SSRI exposure can affect brain development and emotional behavior in offspring, particularly in relation to depression. Both animal and human studies found that early SSRI exposure alters brain structure and increases fear responses, particularly in the amygdala, which is closely linked to anxiety and depression symptoms. These findings suggest that maternal SSRI use during pregnancy may have lasting effects on offspring's mental health, playing an important role in the onset and development of adolescent depression [48, 71, 72]. Given that the study population was restricted to minors, such signals are more likely to reflect reports of maternal drug exposure [73, 74], documentation bias related to perinatal background information, or misclassification of indications and outcomes, rather than direct effects of these medications on the pediatric patients themselves. In addition, these signals are often based on very small absolute numbers of cases, rendering disproportionality estimates potentially unstable from a statistical perspective. By emphasizing Bayesian shrinkage estimates such as EBGM05, the present study aims to minimize overinterpretation of low-frequency events and to clarify that these findings should be regarded as signal-level observations rather than as evidence of established or stable risk.

### 4.6 SSRI Class Effects and Drug-Specific Differences

Comparisons between the present findings and previous pharmacovigilance studies of non-SSRI antidepressants suggest that signals such as suicidality-related behaviors and serotonin syndrome may, to some extent, reflect a class effect associated with enhanced serotonergic activity [75]. However, the observed differences in signal strength and distribution across individual SSRIs indicate that drug-specific structural features, metabolic pathways, and enzyme inhibition profiles may modulate the expression of these class-level signals, either amplifying or attenuating them. This pattern-characterized by a shared class effect overlaid with drug-specific modifiers-may help explain the heterogeneity of safety signal profiles observed among different SSRIs in real-world settings.

### 4.7 Distinguishing Established Signals from Potentially Novel Findings

It should be noted that several signals observed in the present study (such as suicide-related events) are highly consistent with existing regulatory warnings and clinical guidelines, and therefore represent a real- world reconfirmation of previously recognized safety concerns [75]. In contrast, the differentiated signal patterns observed across individual SSRIs in domains such as cardiac electrophysiology, metabolic disturbances, and perinatal-related outcomes-particularly when supported by consistency across multiple disproportionality algorithms and Bayesian shrinkage analyses-offer a comparative perspective that may extend beyond currently well-characterized safety profiles. These findings provide a foundation for future investigations integrating pharmacokinetic, pharmacogenomic, and mechanistic evidence to further contextualize SSRI-associated safety signals in pediatric populations.

## CONCLUSION

This study provides a comprehensive pharmacovigilance analysis of fluoxetine, sertraline, escitalopram, and fluvoxamine in minors with depression using the FAERS database. Our results highlight that psychiatric adverse events-particularly suicidal ideation, suicide attempt, intentional self-injury, and completed suicide-represent the most prominent safety concerns across all four SSRIs. Neurological complications such as serotonin syndrome and tremor also demonstrated strong signals, underscoring the importance of careful monitoring of central nervous system reactions. In addition, cardiac events, including QT interval prolongation, were observed with several SSRIs, and metabolic disturbances such as rhabdomyolysis and metabolic acidosis were most strongly associated with fluvoxamine and escitalopram, respectively. The findings indicate that different SSRIs exhibit distinct safety profiles in minors: fluvoxamine shows the highest risk for psychiatric and neurological abnormalities, escitalopram is strongly associated with developmental and cardiac risks, while Fluoxetine and sertraline share comparable risks in psychiatric and cardiac domains. These differences reflect the unique vulnerability of minors related to neurodevelopment, emotional regulation, and drug metabolism.

Given these insights, clinicians should conduct individualized risk-benefit assessments when prescribing SSRIs to minors, with particular attention to psychiatric monitoring, cardiac safety, and potential systemic complications. Close follow-up, integration of family support, and laboratory monitoring are crucial to optimize therapeutic safety and minimize the risk of severe adverse events. Further prospective studies and integration of real-world data are needed to validate these findings and guide evidence-based, safer treatment strategies for depression in children and adolescents.

## STUDY LIMITATIONS

This study evaluated AE signals for fluoxetine, sertraline, escitalopram, and fluvoxamine in minors with depression using FAERS. The results should be interpreted with several caveats. First, FAERS is a spontaneous (largely voluntary) system prone to under-reporting, selective/notoriety-driven reporting, missing fields, and residual duplicates, introducing reporting and selection bias. In pediatrics, mild or atypical AEs are particularly likely to be overlooked, which may underestimate certain risks, whereas well-publicized events (*e.g.*, suicidality) may be overrepresented. Second, FAERS lacks exposure denominators, dosage, treatment duration, disease severity, and detailed co-medication data; therefore, true incidence cannot be estimated, and between-drug differences may reflect utilization patterns and case-mix (confounding by indication and polypharmacy) rather than pharmacology. Third, misclassification may occur for age, indication, and outcomes (MedDRA PT/SOC assignment), and primary-suspect attribution is uncertain with concomitant therapies; dechallenge/rechallenge and time-to-onset information are often incomplete, limiting causal inference. Fourth, disproportionality metrics (ROR/PRR/IC/EBGM) are signal-detection tools whose outputs are algorithm- and threshold-dependent (*e.g.*, a≥3, ROR/PRR≥2.0, CI lower bound>1), may be unstable for rare PTs and under multiplicity, and do not establish causality. Fifth, generalizability is constrained by the U.S. focus of FAERS and our restriction to depression in minors without finer stratification by developmental stage (children vs adolescents), sex, formulation, or comorbidity, which may mask differential susceptibility. Finally, the analysis emphasized statistical signals and did not integrate external epidemiology or mechanistic context (*e.g.*, pharmacokinetics/pharmacogenomics), limiting biological interpretation.

Future directions: To strengthen inference, future work should triangulate FAERS findings with multi-source real-world data (EHRs/claims/registries) and other pharmacovigilance systems (*e.g.*, VigiBase/EudraVigilance); obtain exposure denominators and conduct stratified analyses by age, sex, comorbidity, and formulation; perform sensitivity analyses (vary thresholds and backgrounds; primary- vs secondary-suspect roles) and incorporate time-to-onset modeling; evaluate drug-drug interactions; and integrate pharmacokinetic/pharmacogenomic and dose-response evidence.

## Figures and Tables

**Fig. (1):**
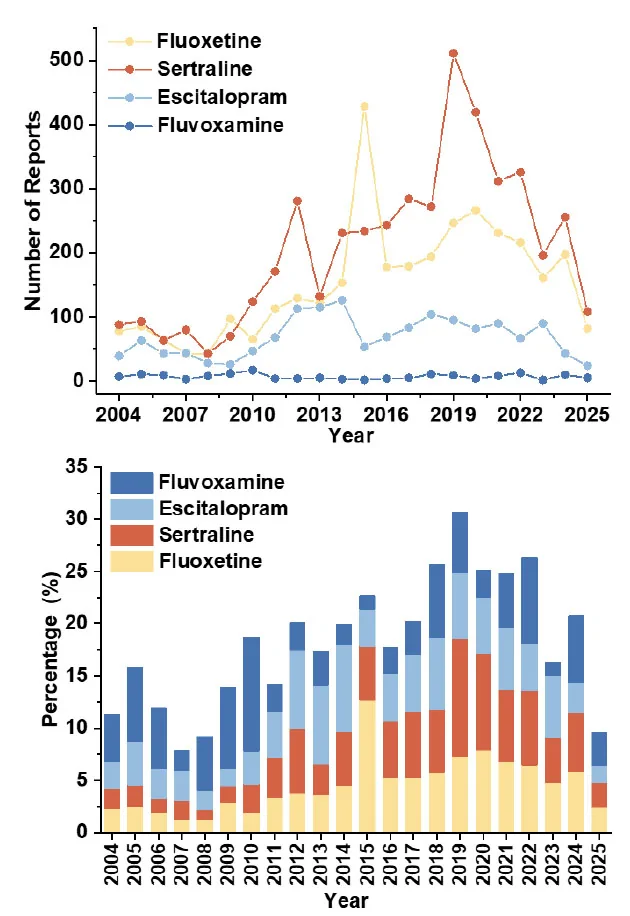
Number of reported adverse effects of fluoxetine, sertraline, escitalopram, and fluvoxamine per quarter from 2004 to 2025.

**Fig. (2):**
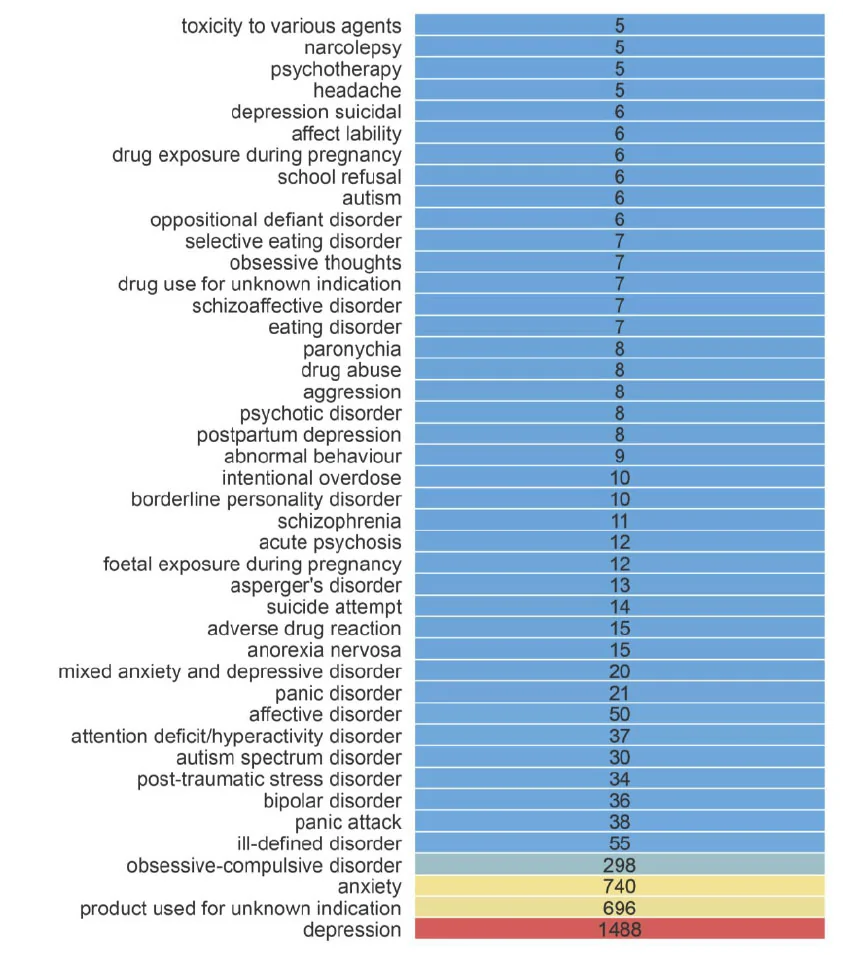
Fluoxetine: Indications Reported ≥5 Times in the FAERS Database.

**Fig. (3):**
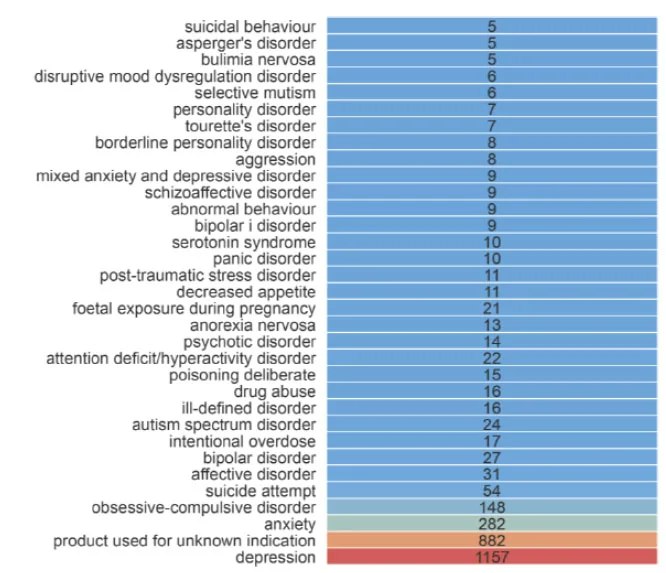
Sertraline: Indications Reported ≥5 Times in the FAERS Database.

**Fig. (4):**
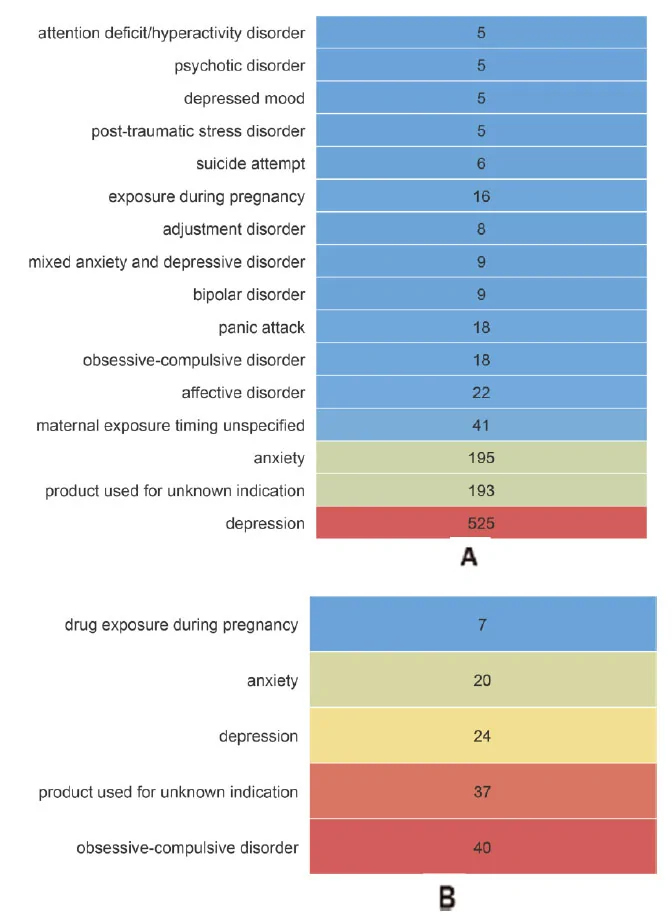
Escitalopram and Fluvoxamine: Indications Reported ≥5 Times in the FAERS Database. (**A)** Escitalopram and Fluvoxamine: Indications Reported ≥5 Times. (**B**) Fluvoxamine: Indications Reported ≥5 Times.

**Table 1:** Four grid table.

	**Drug-related ADEs**	**Non-drug-related ADEs**	**Total**
Drug	a	b	a + b
Non-drug	c	d	c + d
Total	a + c	b + d	N = a + b + c + d
**Method**	**Formula**		﻿**Threshold**
ROR	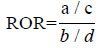		a ≥ 3 ROR ≥ 3 ﻿95%CI (lower limit) > 1
	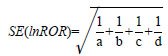		
	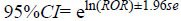		
PRR	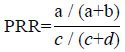		a ≥ 3 PRR ≥ 2 ﻿95%CI (lower limit) > 1
	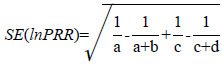		
	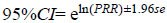		
BCPNN	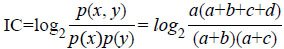		IC025>0
	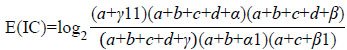		
	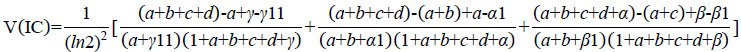		
	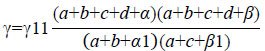		
	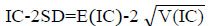		
EBGM	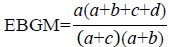		EBGM05>2
	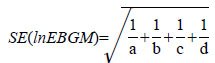		
	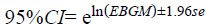		

**Table 2:** Basic information table.

**Variable**	**Fluoxetine** **Total**	**Sertraline** **Total**	**Escitalopram** **Total**	**Fluvoxamine** **Total**
**Age in years (age_yr)**	15.00(10.00,16.00)	14.00(8.00,17.00)	8.00(0.00,16.00)	14.00(11.00,17.00)
**Age group (age_yrQ)**	-	-	-	-
<6	669(22.26)	1060(25.84)	736(53.26)	15(10.42)
6~11	283(9.42)	444(10.82)	60(4.34)	29(20.14)
12~18	2053(68.32)	2598(63.33)	586(42.40)	100(69.44)
**Reporter**	-	-	-	-
Physician	1090(32.34)	1384(30.50)	434(28.70)	59(37.82)
Consumer	895(26.56)	1187(26.16)	328(21.69)	23(14.74)
Other health-professional	581(17.24)	703(15.49)	315(20.83)	22(14.10)
Pharmacist	529(15.70)	745(16.42)	135(8.93)	21(13.46)
unknown	194(5.76)	297(6.55)	68(4.50)	30(19.23)
Lawyer	81(2.40)	220(4.85)	224(14.81)	1(0.64)
Registered Nurse	-	1(0.02)	8(0.53)	-
**Outcomes**	-	-	-	-
other serious	2025(43.27)	2841(48.61)	727(38.38)	92(49.73)
hospitalization	1369(29.25)	1555(26.60)	453(23.92)	55(29.73)
life threatening	413(8.82)	352(6.02)	85(4.49)	11(5.95)
congenital anomaly	404(8.63)	583(9.97)	420(22.18)	7(3.78)
death	252(5.38)	199(3.40)	116(6.12)	16(8.65)
disability	187(4.00)	279(4.77)	57(3.01)	3(1.62)
Required intervention to Prevent Permanent Impairment/Damage	30(0.64)	36(0.62)	36(1.90)	1(0.54)
**Reported countries**	-	-	-	-
other	943(27.98)	1340(29.53)	674(44.58)	156(100.00)
Netherlands	50(1.48)	158(3.48)	-	-
United States	926(27.48)	771(16.99)	426(28.17)	-
United Kingdom	508(15.07)	769(16.95)	-	-
Germany	225(6.68)	446(9.83)	260(17.20)	-
Denmark	-	-	98(6.48)	-
France	192(5.70)	270(5.95)	54(3.57)	-
Sweden	174(5.16)	316(6.96)	-	-
Canada	122(3.62)	75(1.65)	-	-
Italy	121(3.59)	176(3.88)	-	-
Turkey	109(3.23)	104(2.29)	-	-
China	-	112(2.47)	-	-
**route**	-	-	-	-
oral	1459(43.27)	1993(43.77)	555(36.71)	102(65.38)
other	1389(41.19)	1648(36.20)	330(21.83)	54(34.62)
transplacental	511(15.15)	871(19.13)	613(40.54)	-
transmammary	13(0.39)	41(0.90)	14(0.93)	-
**sex**	-	-	-	-
female	1933(57.36)	2640(58.19)	810(53.57)	66(42.31)
male	1135(33.68)	1752(38.62)	650(42.99)	87(55.77)
unknown	302(8.96)	145(3.20)	52(3.44)	3(1.92)
**Time to onset**	10.50(0.00,104.00)	9.00(0.00,218.50)	51.00(0.00,269.00)	8.00(0.00,100.00)
**Time to onset group**	-	-	-	-
<7	504(29.75)	959(34.09)	225(23.56)	32(32.00)
7~28	163(9.62)	242(8.60)	57(5.97)	7(7.00)
28~60	105(6.20)	135(4.80)	47(4.92)	5(5.00)
>=60	335(19.78)	680(24.17)	307(32.15)	25(25.00)
unknow	587(34.65)	797(28.33)	319(33.40)	31(31.00)
**weight**	54.00(32.45,65.77)	45.00(3.68,60.00)	3.82(2.79,56.77)	43.85(29.48,65.00)

**Table 3:** Disproportionality analysis of system organ class-level adverse events for fluoxetine in the treatment of depression in minors.

**soc_english**	**Fluoxetine**						
	**Case Reports**	**ROR(95% CI)**	**PRR(95% CI)**	**Chi sq**	**IC(IC025)**	**EBGM(EBGM05)**	**O/E Shrinkage**
Psychiatric disorders	3275	3.44(3.3, 3.57)	2.9(2.79, 3.02)	4318.43	1.51(1.46)	2.86(2.77)	1.51(1.46, 1.56)
Nervous system disorders	2069	1.75(1.67, 1.83)	1.64(1.58, 1.71)	562.59	0.71(0.64)	1.64(1.57)	0.71(0.64, 0.76)
Injury, poisoning and procedural complications	1615	0.78(0.74, 0.82)	0.81(0.78, 0.84)	87.43	-0.31(-0.38)	0.81(0.77)	-0.31(-0.39, -0.25)
General disorders and administration site conditions	1371	0.65(0.61, 0.68)	0.68(0.64, 0.72)	241.05	-0.56(-0.64)	0.68(0.65)	-0.56(-0.65, -0.49)
Congenital, familial and genetic disorders	892	4.38(4.09, 4.7)	4.18(3.94, 4.43)	2126.92	2.03(1.93)	4.09(3.86)	2.03(1.92, 2.11)
Investigations	760	0.83(0.77, 0.89)	0.84(0.78, 0.91)	25.54	-0.25(-0.36)	0.84(0.79)	-0.25(-0.37, -0.17)
Respiratory, thoracic and mediastinal disorders	686	0.92(0.85, 0.99)	0.92(0.85, 1)	4.72	-0.12(-0.23)	0.92(0.87)	-0.12(-0.24, -0.02)
Gastrointestinal disorders	680	0.59(0.54, 0.63)	0.6(0.55, 0.65)	189.18	-0.72(-0.83)	0.61(0.57)	-0.72(-0.85, -0.63)
Cardiac disorders	585	1.9(1.75, 2.07)	1.87(1.73, 2.02)	238.09	0.89(0.77)	1.86(1.73)	0.89(0.76, 0.99)
Musculoskeletal and connective tissue disorders	408	1.07(0.97, 1.18)	1.07(0.97, 1.18)	1.8	0.09(-0.05)	1.07(0.98)	0.09(-0.07, 0.21)
Pregnancy, puerperium and perinatal conditions	399	2.96(2.68, 3.27)	2.91(2.64, 3.21)	493.87	1.52(1.38)	2.87(2.64)	1.52(1.35, 1.64)
Metabolism and nutrition disorders	327	0.93(0.84, 1.04)	0.93(0.84, 1.03)	1.53	-0.1(-0.26)	0.93(0.85)	-0.1(-0.28, 0.04)
Skin and subcutaneous tissue disorders	275	0.19(0.17, 0.21)	0.2(0.18, 0.22)	956.86	-2.3(-2.47)	0.2(0.18)	-2.3(-2.5, -2.16)
Eye disorders	230	0.85(0.75, 0.97)	0.86(0.75, 0.99)	5.66	-0.22(-0.41)	0.86(0.77)	-0.22(-0.44, -0.06)
Vascular disorders	202	0.71(0.61, 0.81)	0.71(0.62, 0.81)	24.37	-0.49(-0.69)	0.71(0.63)	-0.49(-0.72, -0.32)
Reproductive system and breast disorders	199	1.28(1.11, 1.47)	1.27(1.11, 1.46)	11.82	0.35(0.15)	1.27(1.13)	0.35(0.11, 0.52)
Renal and urinary disorders	193	0.91(0.79, 1.05)	0.91(0.79, 1.04)	1.7	-0.13(-0.34)	0.91(0.81)	-0.13(-0.37, 0.04)
Infections and infestations	185	0.2(0.17, 0.23)	0.21(0.18, 0.24)	605.64	-2.28(-2.49)	0.21(0.18)	-2.27(-2.52, -2.1)
Blood and lymphatic system disorders	115	0.31(0.25, 0.37)	0.31(0.26, 0.37)	179.26	-1.68(-1.94)	0.31(0.27)	-1.67(-1.98, -1.45)
Hepatobiliary disorders	113	0.62(0.51, 0.75)	0.62(0.52, 0.74)	26.19	-0.68(-0.95)	0.62(0.53)	-0.68(-0.99, -0.45)
Ear and labyrinth disorders	55	1.37(1.05, 1.79)	1.37(1.04, 1.8)	5.4	0.45(0.07)	1.36(1.09)	0.44(0, 0.76)
Endocrine disorders	52	0.99(0.76, 1.31)	0.99(0.75, 1.3)	0	-0.01(-0.4)	0.99(0.79)	-0.01(-0.47, 0.32)
Immune system disorders	41	0.19(0.14, 0.26)	0.19(0.14, 0.26)	141.51	-2.37(-2.81)	0.19(0.15)	-2.36(-2.88, -1.99)
Neoplasms benign, malignant and Unspecified (incl cysts and polyps)	35	0.27(0.19, 0.38)	0.27(0.19, 0.38)	68.02	-1.86(-2.34)	0.27(0.21)	-1.85(-2.41, -1.45)

**Table 4:** Disproportionality analysis of system organ class-level adverse events for sertraline in the treatment of depression in minors.

**soc_english**	**Sertraline**						
	**Case Reports**	**ROR(95% CI)**	**PRR(95% CI)**	**Chi sq**	**IC(IC025)**	**EBGM(EBGM05)**	**O/E Shrinkage**
psychiatric disorders	4045	3.49(3.37, 3.61)	2.93(2.87, 2.99)	5441.12	1.53(1.48)	2.88(2.8)	1.53(1.48, 1.57)
nervous system disorders	2569	1.78(1.71, 1.86)	1.67(1.61, 1.74)	741.36	0.73(0.67)	1.66(1.6)	0.73(0.66, 0.78)
injury, poisoning and procedural complications	2236	0.9(0.86, 0.94)	0.91(0.88, 0.95)	22.15	-0.13(-0.2)	0.91(0.88)	-0.13(-0.2, -0.08)
general disorders and administration site conditions	1538	0.59(0.56, 0.62)	0.62(0.6, 0.64)	409.93	-0.68(-0.76)	0.62(0.6)	-0.68(-0.77, -0.62)
congenital, familial and genetic disorders	1280	5.26(4.96, 5.57)	4.96(4.68, 5.26)	3932.2	2.26(2.18)	4.79(4.56)	2.26(2.17, 2.33)
investigations	646	0.56(0.52, 0.61)	0.58(0.54, 0.63)	208.65	-0.78(-0.89)	0.58(0.54)	-0.78(-0.91, -0.69)
respiratory, thoracic and mediastinal disorders	707	0.77(0.71, 0.83)	0.78(0.72, 0.84)	48.2	-0.36(-0.47)	0.78(0.73)	-0.36(-0.49, -0.27)
gastrointestinal disorders	1056	0.75(0.71, 0.8)	0.77(0.73, 0.82)	79.69	-0.38(-0.47)	0.77(0.73)	-0.38(-0.48, -0.3)
cardiac disorders	712	1.9(1.76, 2.04)	1.86(1.72, 2.01)	284.62	0.88(0.78)	1.85(1.73)	0.88(0.76, 0.97)
musculoskeletal and connective tissue disorders	495	1.06(0.97, 1.16)	1.06(0.98, 1.15)	1.63	0.08(-0.05)	1.06(0.98)	0.08(-0.07, 0.19)
pregnancy, puerperium and perinatal conditions	352	2.11(1.9, 2.35)	2.09(1.89, 2.31)	197.54	1.05(0.89)	2.07(1.89)	1.05(0.87, 1.17)
metabolism and nutrition disorders	309	0.71(0.64, 0.8)	0.72(0.64, 0.81)	34.3	-0.47(-0.63)	0.72(0.66)	-0.47(-0.66, -0.33)
skin and subcutaneous tissue disorders	345	0.19(0.17, 0.21)	0.21(0.19, 0.23)	1158.56	-2.27(-2.42)	0.21(0.19)	-2.27(-2.44, -2.14)
eye disorders	365	1.11(1, 1.24)	1.11(1.01, 1.22)	4.12	0.15(0)	1.11(1.02)	0.15(-0.02, 0.28)
vascular disorders	224	0.64(0.56, 0.73)	0.64(0.56, 0.73)	45.33	-0.63(-0.82)	0.64(0.58)	-0.63(-0.85, -0.47)
reproductive system and breast disorders	227	1.19(1.04, 1.36)	1.19(1.04, 1.36)	6.73	0.25(0.06)	1.19(1.06)	0.25(0.03, 0.4)
renal and urinary disorders	211	0.81(0.71, 0.93)	0.81(0.71, 0.93)	9.2	-0.3(-0.49)	0.81(0.73)	-0.3(-0.52, -0.13)
infections and infestations	236	0.2(0.18, 0.23)	0.21(0.19, 0.24)	726.49	-2.22(-2.4)	0.21(0.19)	-2.22(-2.43, -2.06)
blood and lymphatic system disorders	90	0.19(0.16, 0.24)	0.2(0.16, 0.25)	297.93	-2.32(-2.62)	0.2(0.17)	-2.32(-2.66, -2.06)
hepatobiliary disorders	150	0.67(0.57, 0.79)	0.67(0.57, 0.78)	23.77	-0.56(-0.8)	0.68(0.59)	-0.56(-0.83, -0.37)
ear and labyrinth disorders	99	2.03(1.66, 2.48)	2.02(1.66, 2.46)	50.52	1(0.72)	2.01(1.7)	1(0.66, 1.24)
endocrine disorders	59	0.92(0.71, 1.19)	0.92(0.71, 1.19)	0.4	-0.12(-0.49)	0.92(0.74)	-0.12(-0.55, 0.19)
immune system disorders	81	0.31(0.25, 0.38)	0.31(0.25, 0.38)	126.35	-1.68(-2)	0.31(0.26)	-1.68(-2.05, -1.41)
neoplasms benign, malignant and unspecified (incl cysts and polyps)	40	0.25(0.19, 0.35)	0.25(0.18, 0.34)	87.66	-1.96(-2.41)	0.26(0.2)	-1.95(-2.48, -1.57)

**Table 5:** Disproportionality analysis of system organ class-level adverse events for escitalopram in the treatment of depression in minors.

**soc_english**	**Escitalopram**						
	**Case Reports**	**ROR(95% CI)**	**PRR(95% CI)**	**Chi sq**	**IC(IC025)**	**EBGM(EBGM05)**	**O/E Shrinkage**
psychiatric disorders	1074	2.86(2.68, 3.06)	2.5(2.36, 2.65)	1042.66	1.32(1.22)	2.49(2.36)	1.32(1.22, 1.39)
nervous system disorders	521	1.11(1.01, 1.21)	1.1(1.02, 1.19)	4.7	0.13(0)	1.09(1.02)	0.13(-0.01, 0.24)
injury, poisoning and procedural complications	903	1.24(1.15, 1.33)	1.2(1.13, 1.27)	34.56	0.26(0.16)	1.2(1.13)	0.26(0.15, 0.34)
general disorders and administration site conditions	470	0.58(0.53, 0.64)	0.62(0.57, 0.67)	127.38	-0.69(-0.83)	0.62(0.57)	-0.69(-0.84, -0.58)
congenital, familial and genetic disorders	833	12.07(11.2, 13)	10.4(9.81, 11.03)	6992.97	3.34(3.24)	10.15(9.54)	3.34(3.22, 3.42)
investigations	225	0.64(0.56, 0.74)	0.66(0.58, 0.76)	42.21	-0.6(-0.79)	0.66(0.59)	-0.6(-0.82, -0.44)
respiratory, thoracic and mediastinal disorders	213	0.75(0.66, 0.86)	0.76(0.66, 0.87)	16.77	-0.39(-0.59)	0.76(0.68)	-0.39(-0.62, -0.23)
gastrointestinal disorders	239	0.55(0.48, 0.62)	0.57(0.51, 0.64)	85.86	-0.82(-1.01)	0.57(0.51)	-0.82(-1.03, -0.66)
cardiac disorders	192	1.65(1.42, 1.9)	1.62(1.41, 1.86)	46.74	0.7(0.49)	1.62(1.44)	0.69(0.46, 0.87)
musculoskeletal and connective tissue disorders	127	0.88(0.74, 1.05)	0.88(0.74, 1.05)	2.01	-0.18(-0.43)	0.88(0.76)	-0.18(-0.47, 0.03)
pregnancy, puerperium and perinatal conditions	232	4.63(4.05, 5.28)	4.48(3.98, 5.04)	624.82	2.15(1.96)	4.44(3.97)	2.14(1.92, 2.3)
metabolism and nutrition disorders	66	0.5(0.39, 0.63)	0.5(0.4, 0.63)	33.55	-1(-1.34)	0.5(0.41)	-0.99(-1.4, -0.7)
skin and subcutaneous tissue disorders	103	0.19(0.15, 0.23)	0.2(0.16, 0.24)	359.03	-2.31(-2.59)	0.2(0.17)	-2.3(-2.63, -2.07)
eye disorders	56	0.55(0.42, 0.71)	0.55(0.43, 0.71)	20.46	-0.85(-1.23)	0.55(0.44)	-0.84(-1.29, -0.53)
vascular disorders	47	0.43(0.33, 0.58)	0.44(0.33, 0.59)	34.27	-1.18(-1.6)	0.44(0.35)	-1.18(-1.66, -0.83)
reproductive system and breast disorders	71	1.21(0.96, 1.53)	1.21(0.96, 1.53)	2.56	0.27(-0.06)	1.21(0.99)	0.27(-0.12, 0.55)
renal and urinary disorders	29	0.36(0.25, 0.52)	0.36(0.25, 0.52)	32.77	-1.46(-1.98)	0.36(0.27)	-1.44(-2.06, -1)
infections and infestations	61	0.17(0.13, 0.22)	0.18(0.14, 0.23)	241.54	-2.47(-2.83)	0.18(0.15)	-2.46(-2.88, -2.15)
blood and lymphatic system disorders	25	0.18(0.12, 0.26)	0.18(0.12, 0.27)	95.39	-2.47(-3.02)	0.18(0.13)	-2.44(-3.11, -1.97)
hepatobiliary disorders	28	0.41(0.28, 0.59)	0.41(0.28, 0.59)	24.02	-1.28(-1.81)	0.41(0.3)	-1.27(-1.9, -0.82)
ear and labyrinth disorders	7	0.46(0.22, 0.97)	0.46(0.22, 0.97)	4.42	-1.12(-2.12)	0.46(0.25)	-1.06(-2.36, -0.21)
endocrine disorders	9	0.46(0.24, 0.88)	0.46(0.24, 0.88)	5.83	-1.13(-2.02)	0.46(0.26)	-1.09(-2.23, -0.33)
immune system disorders	11	0.14(0.08, 0.24)	0.14(0.08, 0.25)	60.49	-2.86(-3.68)	0.14(0.08)	-2.81(-3.83, -2.11)
neoplasms benign, malignant and unspecified (incl cysts and polyps)	10	0.21(0.11, 0.38)	0.21(0.11, 0.39)	30.32	-2.26(-3.11)	0.21(0.12)	-2.2(-3.28, -1.48)

**Table 6:** Disproportionality analysis of system organ class-level adverse events for fluvoxamine in the treatment of depression in minors.

**soc_english**	**Fluvoxamine**						
	**Case Reports**	**ROR(95% CI)**	**PRR(95% CI)**	**Chi sq**	**IC(IC025)**	**EBGM(EBGM05)**	**O/E Shrinkage**
psychiatric disorders	176	4.4(3.7, 5.23)	3.48(3.09, 3.91)	336.31	1.8(1.56)	3.47(3)	1.79(1.54, 1.97)
nervous system disorders	95	1.84(1.48, 2.29)	1.72(1.44, 2.05)	31.03	0.78(0.47)	1.72(1.43)	0.77(0.43, 1.02)
injury, poisoning and procedural complications	45	0.45(0.34, 0.62)	0.49(0.37, 0.64)	27.44	-1.02(-1.45)	0.49(0.38)	-1.01(-1.51, -0.66)
general disorders and administration site conditions	68	0.74(0.58, 0.95)	0.77(0.62, 0.96)	5.51	-0.38(-0.74)	0.77(0.62)	-0.38(-0.78, -0.09)
congenital, familial and genetic disorders	13	1.49(0.86, 2.58)	1.48(0.87, 2.51)	2.07	0.57(-0.2)	1.48(0.94)	0.54(-0.4, 1.18)
investigations	79	2.07(1.64, 2.63)	1.94(1.56, 2.41)	38.6	0.96(0.62)	1.94(1.6)	0.95(0.58, 1.22)
respiratory, thoracic and mediastinal disorders	10	0.3(0.16, 0.55)	0.31(0.17, 0.57)	16.44	-1.7(-2.56)	0.31(0.18)	-1.65(-2.73, -0.93)
gastrointestinal disorders	29	0.57(0.4, 0.83)	0.59(0.41, 0.84)	8.7	-0.75(-1.28)	0.59(0.43)	-0.74(-1.36, -0.3)
cardiac disorders	12	0.86(0.48, 1.52)	0.86(0.49, 1.52)	0.27	-0.22(-1.01)	0.86(0.53)	-0.21(-1.19, 0.46)
musculoskeletal and connective tissue disorders	30	1.84(1.28, 2.66)	1.8(1.26, 2.56)	11	0.85(0.33)	1.8(1.33)	0.83(0.22, 1.26)
pregnancy, puerperium and perinatal conditions	5	0.85(0.35, 2.04)	0.85(0.35, 2.05)	0.13	-0.24(-1.4)	0.85(0.41)	-0.22(-1.78, 0.77)
metabolism and nutrition disorders	15	0.99(0.59, 1.64)	0.99(0.59, 1.65)	0	-0.02(-0.74)	0.99(0.64)	-0.02(-0.89, 0.58)
skin and subcutaneous tissue disorders	28	0.48(0.33, 0.7)	0.5(0.35, 0.71)	15.26	-1(-1.53)	0.5(0.36)	-0.99(-1.62, -0.54)
eye disorders	12	1.01(0.57, 1.78)	1.01(0.57, 1.78)	0	0.01(-0.78)	1.01(0.62)	0.01(-0.97, 0.68)
vascular disorders	7	0.55(0.26, 1.15)	0.55(0.26, 1.16)	2.62	-0.86(-1.87)	0.55(0.3)	-0.82(-2.12, 0.03)
reproductive system and breast disorders	-	-	-	-	-	-	-
renal and urinary disorders	6	0.63(0.28, 1.4)	0.63(0.28, 1.41)	1.3	-0.66(-1.74)	0.63(0.32)	-0.62(-2.04, 0.29)
infections and infestations	-	-	-	-	-	-	-
blood and lymphatic system disorders	4	0.23(0.09, 0.62)	0.24(0.09, 0.64)	10.1	-2.08(-3.35)	0.24(0.1)	-1.95(-3.71, -0.87)
hepatobiliary disorders	7	0.87(0.41, 1.83)	0.87(0.41, 1.83)	0.14	-0.2(-1.21)	0.87(0.47)	-0.19(-1.49, 0.67)
ear and labyrinth disorders	-	-	-	-	-	-	-
endocrine disorders	-	-	-	-	-	-	-
immune system disorders	-	-	-	-	-	-	-
neoplasms benign, malignant and unspecified (incl cysts and polyps)	-	-	-	-	-	-	-

**Table 7:** Disproportionality analysis of the top 20 adverse events for fluoxetine ranked by case report frequency.

SOC	PT	**Fluoxetine**								
		**Case Reports**	**ROR(95% CI)**	**PRR(95% CI)**	**Chi sq**	**IC(IC025)**	**EBGM(EBGM05)**	**O/E Shrinkage**	***p*.value**	***p*.adj**
injury, poisoning, and procedural complications	foetal exposure during pregnancy	382	3.8(3.43, 4.21)	3.73(3.38, 4.11)	747.32	1.87(1.72)	3.66(3.35)	1.86(1.7, 1.99)	0	0
injury, poisoning, and procedural complications	intentional overdose	346	6.18(5.55, 6.89)	6.06(5.49, 6.68)	1407.44	2.55(2.39)	5.85(5.34)	2.54(2.36, 2.67)	0	0
psychiatric disorders	suicidal ideation	254	6.14(5.41, 6.97)	6.05(5.38, 6.81)	1029.01	2.55(2.36)	5.84(5.25)	2.53(2.32, 2.68)	0	0
psychiatric disorders	intentional self-injury	252	10.99(9.66, 12.51)	10.82(9.62, 12.17)	2088.4	3.34(3.15)	10.12(9.08)	3.31(3.1, 3.46)	0	0
psychiatric disorders	suicide attempt	191	4.38(3.79, 5.06)	4.33(3.77, 4.97)	476.21	2.08(1.87)	4.23(3.75)	2.07(1.83, 2.24)	0	0
general disorders and administration site conditions	drug interaction	181	4.24(3.65, 4.92)	4.2(3.66, 4.82)	429.63	2.04(1.82)	4.11(3.63)	2.03(1.78, 2.2)	0	0
nervous system disorders	sertraline serotonin syndrome	145	23.26(19.5, 27.75)	23.05(19.32, 27.5)	2626.98	4.32(4.07)	19.93(17.19)	4.23(3.95, 4.42)	0	0
psychiatric disorders	completed suicide	143	6.44(5.44, 7.62)	6.38(5.35, 7.61)	622.08	2.62(2.38)	6.15(5.34)	2.59(2.32, 2.79)	0	0
psychiatric disorders	agitation	135	3.04(2.56, 3.61)	3.02(2.53, 3.6)	179.45	1.58(1.33)	2.98(2.58)	1.56(1.28, 1.77)	0	0
nervous system disorders	tremor	117	3.02(2.51, 3.63)	3(2.51, 3.58)	153.51	1.57(1.3)	2.96(2.54)	1.55(1.25, 1.78)	0	0
congenital, familial, and genetic disorders	atrial septal defect	103	5.51(4.52, 6.71)	5.48(4.5, 6.67)	363.46	2.41(2.13)	5.31(4.5)	2.38(2.05, 2.61)	0	0
psychiatric disorders	drug abuse	91	3.38(2.74, 4.16)	3.36(2.71, 4.17)	147.81	1.73(1.43)	3.31(2.78)	1.71(1.36, 1.96)	0	0
psychiatric disorders	confusional state	73	3.14(2.49, 3.97)	3.13(2.47, 3.96)	103.89	1.63(1.29)	3.09(2.54)	1.61(1.22, 1.88)	0	0
nervous system disorders	syncope	66	3.25(2.54, 4.15)	3.24(2.56, 4.1)	99.86	1.67(1.32)	3.19(2.6)	1.65(1.24, 1.94)	0	0
investigations	electrocardiogram qt prolonged	65	3.99(3.12, 5.11)	3.98(3.08, 5.14)	141.16	1.96(1.61)	3.9(3.17)	1.93(1.52, 2.23)	0	0
renal and urinary disorders	urinary retention	65	9.54(7.42, 12.27)	9.5(7.36, 12.26)	463.18	3.16(2.8)	8.96(7.26)	3.08(2.67, 3.37)	0	0
congenital, familial, and genetic disorders	patent ductus arteriosus	60	6.76(5.21, 8.76)	6.74(5.22, 8.7)	279.85	2.69(2.32)	6.47(5.21)	2.63(2.2, 2.94)	0	0
congenital, familial, and genetic disorders	ventricular septal defect	58	5.57(4.28, 7.25)	5.55(4.3, 7.16)	208.51	2.43(2.05)	5.38(4.32)	2.37(1.94, 2.69)	0	0
psychiatric disorders	depressed mood	56	3.58(2.74, 4.66)	3.57(2.71, 4.7)	100.93	1.81(1.43)	3.5(2.8)	1.78(1.33, 2.09)	0	0
injury, poisoning, and procedural complications	maternal drugs affecting the foetus	54	4.24(3.23, 5.56)	4.22(3.21, 5.55)	129.11	2.05(1.66)	4.13(3.29)	2.01(1.55, 2.33)	0	0

**Table 8:** Disproportionality Analysis of the Top 20 Adverse Events for sertraline Ranked by Case Report Frequency.

SOC	PT	**Sertraline**								
		**Case Reports**	**ROR(95% CI)**	**PRR(95% CI)**	**Chi sq**	**IC(IC025)**	**EBGM(EBGM05)**	**O/E Shrinkage**	***p*.value**	***p*.adj**
injury, poisoning, and procedural complications	foetal exposure during pregnancy	490	4.01(3.66, 4.39)	3.93(3.56, 4.33)	1040.88	1.94(1.81)	3.83(3.55)	1.93(1.78, 2.04)	0	0
injury, poisoning, and procedural complications	intentional overdose	439	6.48(5.88, 7.14)	6.35(5.76, 7)	1880.64	2.6(2.46)	6.06(5.59)	2.59(2.43, 2.71)	0	0
psychiatric disorders	intentional self-injury	340	12.45(11.12, 13.94)	12.24(10.88, 13.77)	3173.86	3.48(3.32)	11.15(10.14)	3.46(3.28, 3.59)	0	0
psychiatric disorders	suicide attempt	322	6.18(5.52, 6.92)	6.08(5.41, 6.84)	1302.94	2.54(2.38)	5.83(5.3)	2.53(2.35, 2.67)	0	0
psychiatric disorders	suicidal ideation	265	5.22(4.61, 5.91)	5.16(4.59, 5.8)	851.89	2.32(2.14)	4.98(4.49)	2.3(2.1, 2.45)	0	0
nervous system disorders	Tremor	217	4.67(4.08, 5.36)	4.63(4.04, 5.31)	594.76	2.17(1.97)	4.49(4)	2.15(1.93, 2.32)	0	0
congenital, familial, and genetic disorders	atrial septal defect	199	9.05(7.83, 10.46)	8.96(7.81, 10.28)	1306.67	3.07(2.86)	8.38(7.42)	3.04(2.81, 3.21)	0	0
psychiatric disorders	Agitation	184	3.41(2.94, 3.95)	3.39(2.96, 3.89)	301.49	1.73(1.52)	3.32(2.93)	1.72(1.48, 1.9)	0	0
nervous system disorders	sertraline serotonin syndrome	136	17.57(14.66, 21.06)	17.45(14.63, 20.82)	1830.26	3.93(3.67)	15.27(13.12)	3.86(3.58, 4.06)	0	0
injury, poisoning, and procedural complications	maternal exposure timing unspecified	136	126.18(98.88, 161.02)	125.24(98.99, 158.45)	7995.13	5.91(5.61)	60.25(49.13)	5.63(5.35, 5.83)	0	0
congenital, familial, and genetic disorders	ventricular septal defect	118	9.67(8.01, 11.67)	9.61(7.9, 11.69)	840.3	3.16(2.89)	8.94(7.64)	3.11(2.81, 3.33)	0	0
psychiatric disorders	completed suicide	110	3.98(3.29, 4.82)	3.96(3.26, 4.82)	235.95	1.95(1.68)	3.86(3.29)	1.93(1.62, 2.16)	0	0
injury, poisoning, and procedural complications	maternal exposure during pregnancy	110	6.27(5.17, 7.6)	6.23(5.12, 7.58)	458.8	2.58(2.3)	5.96(5.08)	2.54(2.23, 2.77)	0	0
pregnancy, puerperium, and perinatal conditions	small for dates baby	108	6.97(5.73, 8.47)	6.93(5.7, 8.43)	517.36	2.72(2.44)	6.59(5.6)	2.68(2.37, 2.91)	0	0
psychiatric disorders	drug abuse	106	3.22(2.65, 3.91)	3.21(2.64, 3.91)	156.91	1.65(1.38)	3.15(2.68)	1.64(1.32, 1.87)	0	0
congenital, familial, and genetic disorders	patent ductus arteriosus	102	9.72(7.93, 11.9)	9.67(7.95, 11.76)	731.09	3.17(2.88)	8.99(7.59)	3.11(2.79, 3.35)	0	0
respiratory, thoracic, and mediastinal disorders	respiratory disorder neonatal	94	12.96(10.47, 16.05)	12.9(10.4, 16)	927.66	3.55(3.24)	11.69(9.78)	3.47(3.13, 3.71)	0	0
psychiatric disorders	confusional state	93	3.29(2.67, 4.04)	3.28(2.7, 3.99)	143.16	1.68(1.39)	3.21(2.7)	1.67(1.32, 1.91)	0	0
nervous system disorders	agitation neonatal	80	24.33(19.11, 30.99)	24.23(19.15, 30.65)	1470.22	4.33(3.99)	20.16(16.47)	4.17(3.8, 4.44)	0	0
psychiatric disorders	Tic	78	4.42(3.52, 5.54)	4.4(3.48, 5.57)	197.87	2.1(1.77)	4.28(3.54)	2.07(1.69, 2.34)	0	0

**Table 9:** Disproportionality Analysis of the Top 20 Adverse Events for escitalopram Ranked by Case Report Frequency.

SOC	PT	**Escitalopram**								
		**Case Reports**	**ROR(95% CI)**	**PRR(95% CI)**	**Chi sq**	**IC(IC025)**	**EBGM(EBGM05)**	**O/E Shrinkage**	***p*.value**	***p*.adj**
injury, poisoning, and procedural complications	foetal exposure during pregnancy	337	9.68(8.66, 10.83)	9.1(8.25, 10.04)	2391.09	3.16(3)	8.91(8.12)	3.08(2.9, 3.21)	0	0
congenital, familial, and genetic disorders	atrial septal defect	125	19.15(15.97, 22.97)	18.7(15.68, 22.31)	2001.31	4.16(3.9)	17.89(15.37)	4.01(3.71, 4.22)	0	0
pregnancy, puerperium, and perinatal conditions	small for dates baby	119	26.84(22.24, 32.38)	26.22(21.98, 31.28)	2707.87	4.62(4.35)	24.63(21.05)	4.43(4.13, 4.65)	0	0
psychiatric disorders	suicide attempt	91	20.76(16.78, 25.67)	20.4(16.44, 25.31)	1596.77	4.28(3.98)	19.43(16.27)	2.47(2.13, 2.71)	0	0
injury, poisoning, and procedural complications	Maternal drugs affecting the foetus	79	5.63(4.5, 7.05)	5.56(4.48, 6.9)	292.14	2.46(2.14)	5.5(4.56)	4.08(3.73, 4.33)	0	0
psychiatric disorders	suicidal ideation	79	21.67(17.25, 27.23)	21.35(17.21, 26.49)	1453.83	4.34(4.02)	20.29(16.77)	2.39(2.04, 2.65)	0	0
congenital, familial, and genetic disorders	ventricular septal defect	73	5.28(4.19, 6.67)	5.22(4.13, 6.6)	246.55	2.37(2.04)	5.17(4.25)	4.13(3.75, 4.39)	0	0
injury, poisoning, and procedural complications	intentional overdose	62	3.21(2.49, 4.12)	3.18(2.46, 4.1)	92.22	1.66(1.3)	3.16(2.56)	1.73(1.35, 2.01)	0	0
psychiatric disorders	completed suicide	61	3.35(2.6, 4.31)	3.32(2.57, 4.28)	98.22	1.72(1.36)	3.3(2.67)	2.78(2.36, 3.08)	0	0
psychiatric disorders	depression	58	38.96(29.7, 51.09)	38.51(29.27, 50.67)	1929.73	5.14(4.75)	35.15(28.01)	1.62(1.2, 1.92)	0	0
pregnancy, puerperium, and perinatal conditions	premature baby	55	10.68(8.15, 13.97)	10.57(8.03, 13.91)	464.42	3.37(2.98)	10.32(8.24)	1.58(1.16, 1.88)	0	0
congenital, familial, and genetic disorders	congenital anomaly	50	7.43(5.61, 9.84)	7.36(5.59, 9.68)	270.16	2.86(2.46)	7.24(5.73)	4.72(4.28, 5.03)	0	0
general disorders and administration site conditions	drug withdrawal syndrome neonatal	49	3(2.26, 3.98)	2.98(2.26, 3.92)	64.31	1.57(1.17)	2.97(2.34)	3.19(2.75, 3.51)	0	0
nervous system disorders	tremor	45	25.84(19.09, 34.98)	25.61(19.09, 34.36)	999.21	4.59(4.16)	24.1(18.7)	1.72(1.25, 2.05)	0	0
congenital, familial, and genetic disorders	talipes	45	3.47(2.58, 4.66)	3.45(2.57, 4.63)	77.62	1.78(1.35)	3.42(2.68)	4.22(3.72, 4.57)	0	0
respiratory, thoracic, and mediastinal disorders	respiratory disorder neonatal	40	17.72(12.89, 24.36)	17.59(12.85, 24.07)	599.12	4.08(3.62)	16.87(12.93)	3.77(3.24, 4.14)	0	0
psychiatric disorders	intentional self-injury	38	11.77(8.52, 16.28)	11.69(8.54, 16)	361	3.51(3.05)	11.38(8.68)	1.96(1.42, 2.35)	0	0
congenital, familial, and genetic disorders	patent ductus arteriosus	30	3.98(2.77, 5.7)	3.96(2.78, 5.64)	65.77	1.97(1.46)	3.93(2.91)	3.27(2.73, 3.66)	0	0
investigations	electrocardiogram qt prolonged	28	5.08(3.49, 7.38)	5.05(3.48, 7.33)	89.94	2.32(1.79)	5(3.66)	2.07(1.44, 2.52)	0	0
congenital, familial, and genetic disorders	craniosynostosis	25	47.16(31.12, 71.48)	46.93(31.1, 70.83)	1003.34	5.39(4.81)	42(29.66)	4.51(3.84, 4.98)	0	0

**Table 10:** Disproportionality Analysis of the Top 20 Adverse Events for Fluvoxamine Ranked by Case Report Frequency.

SOC	PT	**Fluvoxamine**								
		**Case Reports**	**ROR(95% CI)**	**PRR(95% CI)**	**Chi sq**	**IC(IC025)**	**EBGM(EBGM05)**	**O/E Shrinkage**	***p*.value**	***p*.adj**
psychiatric disorders	suicide attempt	21	10.65(6.89, 16.47)	10.34(6.72, 15.91)	177	3.36(2.75)	10.3(7.15)	3.08(2.35, 3.59)	0	0
injury, poisoning, and procedural complications	intentional overdose	13	4.66(2.69, 8.07)	4.59(2.7, 7.79)	36.55	2.2(1.43)	4.58(2.89)	2.02(1.08, 2.66)	0	0
nervous system disorders	somnolence	12	3.38(1.91, 5.99)	3.34(1.89, 5.9)	19.76	1.74(0.95)	3.34(2.07)	1.61(0.63, 2.28)	0	0.001
psychiatric disorders	agitation	9	4.72(2.45, 9.13)	4.67(2.45, 8.92)	26.01	2.22(1.32)	4.67(2.69)	1.97(0.83, 2.73)	0	0.001
psychiatric disorders	suicidal ideation	8	4.19(2.09, 8.42)	4.15(2.09, 8.24)	19.17	2.05(1.1)	4.15(2.31)	1.81(0.59, 2.61)	0.001	0.002
general disorders and administration site conditions	drug interaction	8	4.01(2, 8.06)	3.98(2, 7.9)	17.84	1.99(1.04)	3.97(2.21)	1.76(0.54, 2.56)	0.001	0.003
musculoskeletal and connective tissue disorders	rhabdomyolysis	7	14.54(6.89, 30.7)	14.4(6.84, 30.33)	86.82	3.84(2.83)	14.32(7.66)	2.92(1.62, 3.78)	0	0
psychiatric disorders	disinhibition	7	125.31(58.39, 268.91)	123.97(57.72, 266.25)	812.12	6.88(5.85)	117.95(62.26)	3.75(2.44, 4.6)	0	0
investigations	Blood alkaline phosphatase increased	7	19.29(9.13, 40.75)	19.09(9.06, 40.2)	119.15	4.24(3.23)	18.95(10.14)	3.11(1.81, 3.96)	0	0
psychiatric disorders	intentional self-injury	6	4.75(2.12, 10.61)	4.71(2.11, 10.52)	17.54	2.23(1.16)	4.7(2.4)	1.87(0.46, 2.78)	0.002	0.004
investigations	electrocardiogram qt prolonged	6	7.79(3.48, 17.42)	7.72(3.46, 17.24)	35.05	2.95(1.87)	7.7(3.93)	2.35(0.93, 3.26)	0	0.001
investigations	Alanine aminotransferase increased	6	4.55(2.03, 10.16)	4.51(2.02, 10.07)	16.41	2.17(1.1)	4.51(2.3)	1.83(0.41, 2.74)	0.002	0.005
metabolism and nutrition disorders	metabolic acidosis	6	6.75(3.02, 15.1)	6.7(3, 14.96)	29.04	2.74(1.66)	6.68(3.41)	2.22(0.8, 3.13)	0	0.001
eye disorders	papilloedema	6	23.07(10.29, 51.74)	22.87(10.24, 51.08)	124.33	4.5(3.42)	22.66(11.53)	3.09(1.67, 4)	0	0
nervous system disorders	sertraline serotonin syndrome	5	15.28(6.32, 36.93)	15.17(6.28, 36.65)	65.79	3.91(2.75)	15.08(7.2)	2.73(1.16, 3.71)	0	0
psychiatric disorders	completed suicide	5	4.44(1.84, 10.72)	4.42(1.83, 10.68)	13.22	2.14(0.98)	4.41(2.11)	1.75(0.19, 2.74)	0.006	0.011
general disorders and administration site conditions	drug withdrawal syndrome neonatal	5	9.61(3.98, 23.2)	9.54(3.95, 23.05)	38.12	3.25(2.09)	9.51(4.55)	2.42(0.86, 3.41)	0	0.001
psychiatric disorders	impulsive behaviour	5	25.6(10.57, 61.99)	25.41(10.52, 61.38)	116.04	4.65(3.49)	25.15(12)	2.98(1.41, 3.96)	0	0
investigations	Aspartate aminotransferase increased	5	4.24(1.76, 10.24)	4.22(1.75, 10.19)	12.28	2.08(0.91)	4.21(2.02)	1.71(0.14, 2.69)	0.007	0.012
skin and subcutaneous tissue disorders	hyperhidrosis	5	5.85(2.42, 14.12)	5.81(2.41, 14.04)	19.9	2.54(1.37)	5.8(2.78)	2.01(0.45, 3)	0.002	0.004

**Table 11:** Comparative molecular, pharmacokinetic, and metabolic characteristics of selected SSRIs and their potential links to observed adverse event signals.

**Compound**	**MW (Da)**	**Key Functional Groups**	**log*P* (approx.)**	**Brain Penetration**	**Major CYPs**	**CYP Inhibition**	**Half-life**	**Active Metabolite(s)**	**Mechanistic Links to Observed AE Signals**
**Fluoxetine**	~309	Trifluoromethyl group; secondary amine; phenyl ring	High (~4.0)	High	CYP2D6 (major), CYP2C9	Strong CYP2D6 inhibitor	2-4 days / 7-15 days (norfluoxetine)	Norfluoxetine	Long half-life and active metabolite prolong serotonergic exposure → sustained activation, agitation, suicidality; CYP2D6 inhibition increases risk of serotonin syndrome and QT prolongation *via* drug- drug interactions 56, 57
**sertraline**	~306	Dichlorophenyl group; secondary amine	Moderate-high (~3.3)	High	CYP2B6, CYP2C19, CYP3A4	Weak-moderate CYP2D6 inhibition	~26 h / none	None	Moderate lipophilicity and brain penetration → CNS activation (tremor, serotonin syndrome); a relatively balanced PK profile explains broad but less extreme AE signals
**escitalopram**	~324	Isobenzofuran ring; tertiary amine	Moderate (~3.0)	High	CYP2C19 (major), CYP3A4, CYP2D6	Weak CYP inhibition	~27-32 h / none	None	CYP2C19-dependent clearance → exposure variability; high brain availability + cardiac ion channel sensitivity → enriched QT prolongation and severe psychiatric AE signals59
**Fluvoxamine**	~318	Methoxy group; oxime ether; aromatic rings	Moderate (~2.8)	Moderate	CYP1A2 (major), CYP2D6	Strong CYP1A2 inhibitor	~15-20 h / none	None	σ1 receptor affinity and CYP1A2 inhibition → altered cortical-limbic regulation and drug accumulation → impulsivity, disinhibition, rare but severe systemic AEs (rhabdomyolysis, metabolic acidosis)60, 67

**Note:** Molecular structures, pharmacokinetic parameters, CYP metabolism and inhibition profiles, and receptor-related properties were summarized from previously published pharmacological and clinical literature. The proposed mechanistic links to observed adverse event signals are intended to provide pharmacological context for signal interpretation rather than to imply causality.

## Data Availability

The dataset supporting the conclusions of this article is available *via* the public FDA Adverse Event Reporting System database found at https://www.fda.gov/drugs/questions-and-answers-fdas-adverse-event-reporting-system-faers/fda-adverse-event-reporting-system-faers-public-dashboard.The ‘FAERS Public Dashboard’ option was then selected, prompting to its home page. The ‘Search’ tab was then selected, and Additionally, our dataset comes from the public database, which can be found at https://fis.fda.gov/extensions/FPD-QDE-FAERS/FPD-QDE-FAERS.html. The rest of the information can be found within the Methods section of the manuscript. If readers require further information regarding the data analysis, they can email the corresponding author or the first author for inquiries.

## LIST OF ABBREVIATIONS

SSRIs: Serotonin Reuptake Inhibitors
SOC: System Organ Class
ROR: Reporting Odds Ratio
PRR: Proportional Reporting Ratio
BCPNN: Bayesian Confidence Propagation Neural Network
IC: Information Component

## STANDARDS OF REPORTING

STROBE guidelines were followed.
