# Supplementary material for: Pharmacovigilance of SSRIs in Children and Adolescents with Depression: Disproportionality Analysis of Serotonin Adverse Events Using the FAERS Database
Source: Curr Med Chem. 2026 May 22;33(22):4678–706. doi: 10.2174/0109298673440558260406211258 (PMC13684978; doi:10.2174/0109298673440558260406211258)
Supplement: Supplementary file 1 [file CMC-33-22-4678_SD1.zip › CMC-33-22-4678_SD1/CMC-33-22-11-Supply/BMS-CMC-2025-754-(S Table 1).pdf]

Supplementary Material

Pharmacovigilance of SSRIs in Children and Adolescents with Depression: Disproportionality Analysis of Serotonin Adverse Events Using the FAERS Database

Hongyi Du[1], Jiabei Liu[2], Ruihua Zhou[2], Chenghui Kuang[2] and Ke Ma[3,*]

[1]College of Traditional Chinese Medicine, Shandong University of Traditional Chinese Medicine, Jinan, 250355, P.R. China; [2]College of Acupuncture and Massage, Shandong University of Traditional Chinese Medicine, Jinan, 250355, P.R. China; [3]Shandong Co-Innovation Center of Classic TCM Formula, Shandong University of Traditional Chinese Medicine, Jinan, 250355, P.R. China

Table S1. The dictionary used for filtering Fluvoxamine was applied.

drugname(9220)	prod_ai	AE
luvox	fluvoxamine maleate	3092
fluvoxamine	fluvoxamine; fluvoxamine maleate	2192
fluvoxamine maleate	fluvoxamine maleate	1794
fluvoxamine maleate.	fluvoxamine maleate	505
depromel	fluvoxamine; fluvoxamine maleate	454
fevarin	fluvoxamine maleate	192
luvox cr	fluvoxamine maleate	162
floxyfral	fluvoxamine	155
relafin	fluvoxamine maleate	72
fluvoxamin	fluvoxamine maleate; fluvoxamine	39
fluvoxamine (non-specific)	fluvoxamine	38
dumirox	fluvoxamine maleate	35
fluvoxamine maleate tablets, usp	fluvoxamine maleate	24
faverin	fluvoxamine maleate	19
fluvoxamine maleate 50 mg	fluvoxamine maleate	17
maveral	fluvoxamine maleate	16
fluvoxamine (fluvoxamine)	fluvoxamine	13
fluvoxamine maleate 100 mg	fluvoxamine maleate	13
depromel (fluvoxamine maleate)	depromel (fluvoxamine maleate)	11
fluvoxamine (unknown)	fluvoxamine	11
apo-fluvoxamine	fluvoxamine maleate	11
dumyrox	fluvoxamine	10
fluvoxamine (watson laboratories)	fluvoxamine	9
fluvoxamine maleate 50 mg tablets	fluvoxamine maleate	9
fluoxamine	fluvoxamine	9
luvox [levofloxacin hemihydrate]	levofloxacin; fluvoxamine	9

luvox (fluvoxamine maleate)	fluvoxamine	8
fluvoxamina	fluvoxamine	8
fluvoxamine maleate 25 mg	fluvoxamine maleate	7
fluvoxaminemaleaat	fluvoxamine maleate	7
alti-fluvoxamine	fluvoxamine	7
movox	fluvoxamine maleate	7
voxam	fluvoxamine maleate	7
luvox (fluvoxamine maleate) tablet	fluvoxamine	6
fluvoxamine (ngx)	fluvoxamine	6
fluvoxamine maleate tablets	fluvoxamine maleate	5
fluvoxamina eg	fluvoxamine	5
luvox 100	fluvoxamine	5
suvoxamine	fluvoxamine	5
luvox ir (fluvoxamine maleate) (tablets) (fluvox-amine maleate)	; fluvoxamine maleate; fluvoxamine	4
fluvoxamine maleate (fluvoxamine)	fluvoxamine maleate	4
fluvoxamine maleate (aellc)	fluvoxamine maleate	4
fevarin 50 mg	fluvoxamine	4
fluvox	fluvoxamine maleate	4
luvox [fluvoxamine maleate]	fluvoxamine maleate; fluvoxamine	4
fluvoxamine maleate (fluvoxamine maleate) (fluvoxamine maleate)	fluvoxamine maleate	3
fluvoxaminemaleaat apotex, tabletten	fluvoxamine maleate	3
luvox cr (capsules) (fluvoxamine maleate)	; luvox cr	3
depromel (fluvoxamine) tablet	fluvoxamine	3
fevarin (fluvoxamine maleate)	fluvoxamine	3
depromel (fluvoxamine) tablets	fluvoxamine	3
fluvoxamine (ngx) (fluvoxamine)	fluvoxamine	3
depromel 25 (fluvoxamine maleate)	fluvoxamine maleate	3
fluvoxamine maleate 25 mg tablets	fluvoxamine maleate	3
fluvoxamine (fluvoxamine) tablets	fluvoxamine	3
fluvoxamine (maleate de)	fluvoxamine maleate	3
floxyfral m bruchrille	fluvoxamine maleate	3
depromel (fluvoxamine)	fluvoxamine	3
novo-fluvoxamine	novo-fluvoxamine	3
luvox (con.)	fluvoxamine	3
avoxin	fluvoxamine	3
novo?fluvoxamine	fluvoxamine	3
relafin (fluvoxamine maleate) (fluvoxamine maleate)	fluvoxamine maleate	2

floxyfral 50 mg, comprim? pellicul?	fluvoxamine maleate	2
fluvoxamine (generic) (fluvoxamine)	fluvoxamine	2
floxyfral 100 (fluvoxamine maleate)	fluvoxamine	2
apo-fluvoxamine (fluvoxamine maleate)	fluvoxamine	2
fluvoxamine (fluvoxamine) (50 milligram)	fluvoxamine	2
fluvoxamine maleate (fluvoxamine maleate)	fluvoxamine maleate	2
maveral (fluvoxamine maleate)	fluvoxamine maleate	2
faverin (fluvoxamine maleate)	fluvoxamine	2
fluvoxamin [fluvoxamine]	fluvoxamin [fluvoxamine]	2
fluvoxamine 100mg tablet	fluvoxamine	2
fluvoxamine maleate (con.)	fluvoxamine maleate	2
fluvoxamine 100 mg	fluvoxamine	2
fluvoxamine er	fluvoxamine	2
fluvoxamine eg	fluvoxamine	2
fluvoxamine 100mg	fluvoxamine	2
fluvoxamine maleate np	fluvoxamine maleate	2
fluvoxamine maleate (fluvoxamine maleate) tablet	fluvoxamine maleate (fluvoxamine maleate) tablet	1
fluvoxamina eg 50 mg compresse rivestite con film	fluvoxamine maleate	1
fluvoxamine er 150mg cap/generic for luvox cr 150mg	fluvoxamine	1
depromel 25 (fluvoxamine maleate immediate release)	fluvoxamine maleate	1
luvox ir (500 mg/ml, tablets) (fluvoxamine maleate)	fluvoxamine;	1
dumirox 100 mg comprimidos recubiertos con pel?cula.	fluvoxamine maleate	1
luvox ir (fluvoxamine maleate) (tablets) (fluvoxamine	fluvoxamine; fluvoxamine maleate	1
fluvoxamine (phenytoin sodium injection) (fluvoxamine)	fluvoxamine; phenytoin sodium	1
maveral - 50 mg compresse rivestite con film - abbott s.r.l	fluvoxamine maleate	1
luvox (fluvoxamine maleate) (tablets) (fluvoxamine maleate)	; fluvoxamine	1
floxyfral (floxyfral - fluvoxamine maleate) (not specified)	fluvoxamine maleate; fluvoxamine	1
fluvoxamine (watson laboratories)(fluvoxamine maleate) tablet	; fluvoxamine	1
fluvoxamine (watson laboratories) (fluvoxamine maleate) tablet	; fluvoxamine	1
luvox ir (fluvoxamine maleate) (tablets) (fluvoxamine maleate) (tablet	; fluvoxamine maleate; fluvoxamine	1

fluvoxamine maleate tablet 75mg tyk	fluvoxamine maleate	1
floxyfral 100 mg, comprim? pellicul?	fluvoxamine maleate	1
fluvoxamine maleate extended-release	fluvoxamine maleate	1
depromel (fluvoxamine) tablet, 25 mg	fluvoxamine	1
floxyfral 100 mg comprim?s pellicul?s	fluvoxamine maleate	1
fluvoxamine 100mg ani pharmaceuticals	fluvoxamine maleate	1
fluvoxamine unk, unknown manufacturer	fluvoxamine	1
floxyfral (fluvoxamine maleate) tablet	fluvoxamine	1
fluvoxamine maleate 50 mg ivax/ zenith	fluvoxamine	1
fluvoxamine (fluvoxamine) (fluvoxamine)	fluvoxamine	1
uvox ir (tablets) (fluvoxamine maleate)	uvox ir (tablets) (fluvoxamine maleate)	1
fluvoxamine maleate (fluvoxamne maleate)	maleate; fluvoxamine maleate	1
fluvoxamine (fluvoxamine) (150 milligram)	fluvoxamine	1
fevarin tablet 50 mg (fluvoxamine maleate)	; fluvoxamine	1
fluovoxamine maleate (fluvoxamine maleate)	maleate	1
fluvoxamine?drug use for unknown indication	fluvoxamine?drug use for unknown indication	1
dumirox 100 mg compresse rivestite con film	fluvoxamine maleate	1
amlodipine besilate and fluvoxamine maleate	amlodipine besylate; fluvoxamine maleate	1
fluvoxamine maleate 50 mg ani pharmaceuticals	fluvoxamine maleate	1
maveral - 100 mg compresse rivestite con film	fluvoxamine maleate	1
fluvoxamine maleate tablets, 100 mg (purepac)	fluvoxamine maleate tablets, 100 mg (purepac)	1
fluvoxamine (ngx) (fluvoxamine) tablet, 100mg	fluvoxamine	1
fluvoxamine maleate 50 md bid	fluvoxamine maleate 50 md bid	1
fluvoxamine maleate (unknown)	fluvoxamine maleate	1
unknown (fluvoxamine) unknown	fluvoxamine	1
fluvoxamine maleate (purepac)	fluvoxamine maleate	1
dumyrox (fluvoxamine maleate)	fluvoxamine maleate	1
luvox ir (fluvoxamine meleate)	fluvoxamine	1
fluvoxamine maleate fluvoxamine	fluvoxamine maleate	1
fluvoxamine (fluvoxamine) 200mg	fluvoxamine	1
fluvoxamine maleate 50 mg aptx2	fluvoxamine	1
fluvoxamine maleate 100 mg barr	fluvoxamine	1
floxyfral (fluvoxamine maleate)	fluvoxamine	1
fluvoxamin (fluvoxamine maleate)	fluvoxamine maleate	1
fluvoxamine [fluvoxamine maleate]	fluvoxamine [fluvoxamine maleate]	1
fluvoxamine maleate tablets 50 mg	fluvoxamine maleate tablets 50 mg	1
luvox (fluvoxamine maleate)tablet	fluvoxamine	1
fluvoxamine 50 mg oraal om de dag;	fluvoxamine 50 mg oraal om de dag;	1
fluvoxamine (100 mg) (fluvoxamine)	fluvoxamine	1

apo-fluvoxamine tablets	fluvoxamine maleate	1
fluvoxamine 150 mg -udl	fluvoxamine	1
fluvoxamine cap 100mg er	fluvoxamine cap 100mg er	1
fluvoxamine 50mg tablets	fluvoxamine 50mg tablets	1
fluxamine maleate, 50 mg	fluvoxamine maleate	1
fluvoxamine (fluoxamine)	fluvoxamine	1
fluvoxamin (fluvoxamine)	fluvoxamine	1
fluvoxamine maleate mylan	fluvoxamine maleate mylan	1
fluvoxamine er 150 mg cap	fluvoxamine	1
movox/fluvoxamine maleate	fluvoxamine maleate	1
fluvoxamine (flovoxamine)	fluvoxamine	1
flovoxamine (fluvoxamine)	fluvoxamine	1
fluvoxamine (non?specific)	fluvoxamine	1
fluvoxacmine (fluvoxamine)	fluvoxamine	1
luvox (fluoxetine maleate)	maleate; fluoxetine; fluvoxamine	1
luvox (fluoxamine maleate)	maleate; ; fluvoxamine	1
revoc (fluvoxamine maleate)	fluvoxamine maleate	1
fluvoxamine 150 mg po daily	fluvoxamine 150 mg po daily	1
fluvoxamine (no pref. name)	fluvoxamine	1
luvox (fluvoxamine maleate0	fluvoxamine	1
luvox (fluvoxamide maleate)	maleate; fluvoxamine	1
lovox (fluvoxamine maleate)	fluvoxamine maleate	1
fluvoxamine maleate (luvox)	fluvoxamine maleate	1
luvox 9fluvoxamine maleate)	maleate; fluvoxamine	1
fluvoxamine amleate	fluvoxamine amleate	1
luvox (fluvoxamine)	fluvoxamine	1
luvox -fluvoximine-	fluvoxamine	1
luvox (flovoxamine)	fluvoxamine	1
fluvoxamine (luvox)	fluvoxamine	1
floxyfral bruchrille	fluvoxamine maleate	1
luvox (50 milligram)	fluvoxamine	1
fluvoxamine malelate	fluvoxamine	1
fluvoxamine 150 mg	fluvoxamine	1
fevarin (fluvoxamine)	fluvoxamine	1
fluvoxamine (tablets)	fluvoxamine	1
flox-ex (fluvoxamine)	fluvoxamine	1
depromel [fluvoxamine]	fluvoxamine	1
fluvoxamine maleate er	fluvoxamine maleate er	1
fluvoxamine 100 mg eon	fluvoxamine	1

fluvoxamine ga	fluvoxamine ga	1
fevarin 100 mg	fluvoxamine	1
act-fluvoxamine	fluvoxamine	1
fluvoxamine 25m	fluvoxamine 25m	1
floxyfral 100 mg	fluvoxamine maleate	1
ratio-fluvoxamine	fluvoxamine	1
lavox/fluvoxamine	lavox/fluvoxamine	1
fluvoxamine, 100mg	fluvoxamine, 100mg	1
fluvoxamine tablet	fluvoxamine	1
fluvoxamina (159a)	fluvoxamine	1
luvox tablet	fluvoxamine	1
luvox sa	fluvoxamine	1
movox 100	fluvoxamine maleate	1
fluvoxaminemaleaat mylan 50 mg, tabletten	fluvoxamine maleate	1
fluvoxamine maleate 100mg tabs	fluvoxamine maleate	1
fluvoxamine 150mg	fluvoxamine	1
dumirox 50 mg comprimidos recubiertos con pel?cula.	fluvoxamine maleate	1
fluvoxamine maleate 150mg	fluvoxamine	1
fluvoxamine (fluvoxamine maleate)	fluvoxamine maleate	1
flvoxamine	fluvoxamine	1
fluvoxamine 25mg	fluvoxamine	1
fluvoxamine 50 mg po	fluvoxamine	1
fluvoxamine tab	fluvoxamine	1
fluvoxamine propionate	propionate; fluvoxamine	1
depromel [fluvoxamine maleate]	fluvoxamine maleate	1

Table S2. The dictionary used for filtering Sertraline was applied.

drugname(204085)	prod_ai	AE个数
zoloft	sertraline hydrochloride	95188
sertraline	sertraline hydrochloride	67873
sertraline hcl	sertraline hydrochloride	16668
sertraline hydrochloride	sertraline hcl	10507
sertraline hydrochloride.	sertraline hydrochloride	4417
jzoloft	sertraline hydrochloride	1031
lustral	sertraline hydrochloride	556
sertralin	sertraline hydrochloride	417
sertraline hydrochloride tablets	sertraline hydrochloride	389
sertraline (sertraline)	sertraline	368

zoloft (sertraline hydrochloride)	sertraline	281
sertraline hydrochloride (unknown)	sertraline hydrochloride	242
sertraline 50mg	sertraline hydrochloride	223
sertraline (sertraline) (sertraline)	sertraline	218
sertaline	sertraline hydrochloride	207
besitran	sertraline hydrochloride	169
serlain	sertraline hydrochloride	160
altruline	sertraline hydrochloride	153
sertraline (unknown)	sertraline	136
apo-sertraline	sertraline hydrochloride	128
sertralina	sertraline hydrochloride	120
gladem	sertraline hydrochloride	116
sertraline hydrochloride tablets usp, 50 mg	sertraline hydrochloride	95
sertraline hydrochloride tablets usp, 100 mg	sertraline hydrochloride	95
sertraline hydrochloride tablets 100mg	sertraline hydrochloride	90
asentra	sertraline hydrochloride	87
tresleen	sertraline hydrochloride	84
sertralin stada	sertraline hydrochloride	76
sertraline hydrochloride tablets 25mg	sertraline hydrochloride	71
sertraline (chlorhydrate de)	sertraline hydrochloride	68
serenata	sertraline hydrochloride	62
zoloft (sertraline)	sertraline	53
sertralina (2537a)	sertraline hydrochloride	48
sertraline accord	sertraline hydrochloride	48
zoloft p	sertraline hydrochloride	48
aremis	sertraline hydrochloride	48
sertraline hydrochloride tablets usp, 25 mg	sertraline hydrochloride	47
sertralin [sertraline]	sertraline hydrochloride	47
sertraline tablets usp 50 mg	sertraline hydrochloride	44
sertraline tablets usp 100 mg	sertraline hydrochloride	40
sertralin basics	sertraline hydrochloride	37
sertraline hcl (sertraline hydrochloride)	sertraline hcl (sertraline hydrochloride)	35
jzoloft 25mg	sertraline hydrochloride	35
sertraline hydrochloride (sertraline hydrochloride)	sertraline hydrochloride	34
tolrest	sertraline hydrochloride	34
sertraline 50mg tablets	sertraline hydrochloride	32
sertraline actavis	sertraline hydrochloride	32
sertraline (ngx)	sertraline hydrochloride	32

sertraline hydrochloride oral concentrate 20 mg/ml	sertraline hydrochloride	31
sertraline hydrochloride tablets 50mg	sertraline hydrochloride	31
lustral (sertraline)	sertraline	31
serdep	sertraline hydrochloride; fluoxetine hydrochloride	31
sertraline [sertraline hydrochloride]	sertraline hydrochloride	30
sertraline aurobindo	sertraline hydrochloride	30
daxid	sertraline hydrochloride	30
serlift	sertraline hydrochloride	30
zoloft (sertraline hydrochloride) (sertraline hydrochloride)	zoloft (sertraline hydrochloride) (sertraline hydrochloride)	29
zoloft (sertraline hydrochloride) (unknown)	zoloft (sertraline hydrochloride) (unknown)	29
gladem (sertraline)	gladem (sertraline)	29
seralin	sertraline hydrochloride	29
sertraline (sertraline hydrochloride)	sertraline hcl	28
sertraline tablets usp 25 mg	sertraline hydrochloride	28
retalin	sertraline hydrochloride	28
sertraline arrow	sertraline hydrochloride	27
sertraline oral concentrate	sertraline hydrochloride	26
seralin mepha	sertraline hydrochloride	26
sertraline hci	sertraline hci	26
serolux	sertraline hydrochloride	26
sertraline film-coated tablets 100mg	sertraline hydrochloride	25
adjuvin	sertraline hydrochloride	25
sertralina [sertraline]	sertraline hydrochloride	24
serlife	sertraline hydrochloride	24
sertraline (unknown) (sertraline)	sertraline	22
sertraline 50 mg	sertraline hydrochloride	22
serdep (sertraline hydrochloride)	sertraline hydrochloride	21
generic zoloft	sertraline hydrochloride	21
selectra	sertraline hydrochloride	21
setraline 100mg	sertraline hydrochloride	20
sertralin hexal	sertraline hydrochloride	19
sertralin pfizer	sertraline hydrochloride	19
sertrone	sertraline hydrochloride	19
sertra	sertraline hydrochloride	19
sertraline hydrochloride (watson laboratories)	sertraline hcl	17
zoloft (sertraline hydrochloride) (tablets)	sertraline	17
zoloft (sertraline) (sertraline)	sertraline	17

sertraline 50 mg tablets	sertraline hydrochloride	17
sertralin actavis	sertraline hydrochloride	17
eleva	sertraline hydrochloride	17
jzoloft (sertraline hydrochloride)	jzoloft (sertraline hydrochloride)	16
jzoloft 50mg	sertraline hydrochloride	16
sertraline/sertraline hydrochloride	sertraline hydrochloride	14
sertralin basics 50mg filmtabletten	sertraline hydrochloride	14
zoloft (con.)	sertraline	14
setralin	sertraline hydrochloride	14
zolotrin	sertraline hydrochloride	14
sertraline 50 mg film-coated tablets	sertraline hydrochloride	13
sertraline (sertraline) (unknown)	sertraline	13
sertraline hcl sertraline	sertraline hydrochloride	13
sertraline(sertraline)	sertraline	13
sertralina actavis	sertraline hydrochloride	13
serlain (sertraline)	sertraline	13
sertraline tab	sertraline hydrochloride	13
jzoloft od	sertraline hydrochloride	13
sertralinum	sertraline hydrochloride	13
sertraline (formulation unknown) (generic) (sertraline)	sertraline	11
sertralin aurobindo	sertraline hydrochloride	11
tresleen (sertraline)	sertraline	11
setralin sandoz	sertraline hydrochloride	11
sertralin hcl	sertraline hydrochloride	11
sertralina mylan	sertraline hydrochloride	11
sertraline tablet	sertraline hydrochloride	11
sertraline mylan	sertraline hydrochloride	11
sertraline (sertaline)	sertraline	10
blinded sertraline hcl	sertraline hcl	10
sertralin krka	sertraline hydrochloride	10
sertraline eg	sertraline hydrochloride	10
sertralin sandoz	sertraline hydrochloride	10
sertraline 100 mg	sertraline 100 mg	10
sertagen	sertraline hydrochloride	10
sertraline hcl (sertraline hydrochloride) (unknown)	sertraline hcl	9
sertaline hc1 tablets/ mfr unknown	sertraline hydrochloride	9
sertralin/sertraline hydrochloride	sertralin/sertraline hydrochloride	9

sertralin-hormosan 50 mg filmtabletten	sertraline hydrochloride	9
sertraline hydrochloride teva	sertraline hydrochloride	9
actavis group sertraline	sertraline hydrochloride	9
sertralina teva	sertraline hydrochloride	9
asertin	sertraline hydrochloride	9
sertraline (sertraline hcl 100mg tab)	sertraline hydrochloride	9
sertraline hydrochloride tablets, 100 mg (base) (aellc) (sertraline)	sertraline hcl; sertraline	8
sertraline film-coated tablets 50 mg	sertraline hydrochloride	8
sertraline arrow capsules 50mg	sertraline hydrochloride	8
sertraline 100mg tablet	sertraline hydrochloride	8
sertraline (zoloft	sertraline	8
sertralin/sertraline	sertralin/sertraline	8
seralin-mepha	sertraline hydrochloride	8
teva-sertraline	sertraline hydrochloride	8
sertraline (con.)	sertraline	8
oralin	sertraline hydrochloride	8
sertraline aurobindo filmcoated tablets 100mg	sertraline hydrochloride	7
accord healthcare sertraline	sertraline hydrochloride	7
sertraline (sertraline) unknown	sertraline	7
zoloft 9sertraline hydrochloride)	hydrochloride; sertraline	7
aremis (sertraline hydrochloride)	sertraline hydrochloride	7
sertraline biogaran	sertraline hydrochloride	7
norsertraline	desmethylsertraline	7
sertralin teva	sertraline hydrochloride	7
luxeta	sertraline hydrochloride	7
setaloft	sertraline hydrochloride	7
auro sertraline	sertraline; carbamide peroxide	7
sertraline aurobindo film-coated tablets 50 mg	sertraline hydrochloride	6
lustral (sertraline hydrochloride)	sertraline	6
besitran (sertraline hydrochloride)	besitran (sertraline hydrochloride)	6
sertraline 50 mg film?coated tablets	sertraline hydrochloride	6
zoloft (sertraline hydrochloride) tablet	sertraline	6
sertraline 50mg (sertraline) unknown, 50mg	sertraline hydrochloride	6
zoloft(sertraline hydrochloride)	zoloft(sertraline hydrochloride)	6
sertraline hydrochloride (aellc)	sertraline hydrochloride	6
sertralin (sertraline)	sertraline	6
sertraline 50mg camber p	sertraline	6
sertraline hcl tabs 100mg	sertraline hydrochloride	6

sertraline tablets	sertraline	6
sertraline (zoloft)	sertraline (zoloft)	6
sertraline (generic)	sertraline hydrochloride	6
serenata (sertraline)	sertraline	6
sertralina cloridrato	sertraline hydrochloride	6
pms-sertraline	pms-sertraline	6
zoloft generic	sertraline hydrochloride	6
pms sertraline	pms sertraline	6
teva sertraline	sertraline hydrochloride	6
sertralin accord	sertraline hydrochloride	6
sertraline apotex	sertraline hydrochloride	6
sertraline pfizer	sertraline hydrochloride	6
dieloft	sertraline hydrochloride	6
insertec	sertraline hydrochloride	6
lustral (sertraline hydrochloride) unknown	sertraline	5
cloridrato de setraline 50mg	sertraline hydrochloride	5
zoloft (sertraline hydrchloride)	sertraline	5
setraline (sertraline)	setraline (sertraline)	5
sertraline 25mg tablet amel	sertraline hydrochloride	5
sertralina alter	sertraline hydrochloride	5
sertraline sandoz	sertraline	5
sertraline h	sertraline h	5
sertraline a	sertraline hydrochloride	5
jazoloft	sertraline hydrochloride	5
zoloft (sertraline hydrochloride) tablet, 25 mg	sertraline	4
sertraline 50 ranbaxy, filmomhulde tabletten 50 mg	sertraline hydrochloride	4
sertraline hcl (sertraline hydrochloride) (tablets)	sertraline hcl	4
sertraline hcl (sertraline hydrochloride) (ser-traline hydrochloride)	sertraline hcl	4
sertralin dura 100 mg filmtabletten	sertraline hydrochloride	4
sertralin [sertraline hydrochloride]	sertralin [sertraline hydrochloride]	4
sertralin basics 100mg filmtabletten	sertraline hydrochloride	4
sertraline (ngx) (sertraline) unknown	sertraline	4
sertraline hydrochloride tablets, usp	sertraline hydrochloride	4
sertraline hydrochloride (base) (aellc)	sertraline hcl	4
sertralin ranbaxy 50 mg film-coated tablet	sertraline hydrochloride	4
sertraline 25 mg not available	sertraline	4
sertraline hcl (zoloft)	sertraline hcl (zoloft)	4

sertaline hydrochloride	sertraline hydrochloride	4
sertraline (sertraline)	sertraline	4
sertraline 100 mg tablets	sertraline hydrochloride	4
sertraline 50mg aurobindo	sertraline hydrochloride	4
sertralin krka/sertraline	sertraline hydrochloride	4
chlorhydrate de sertraline	sertraline hydrochloride	4
sertraline hcl 50mg tablet	sertraline hydrochloride	4
sertralin 1a farma	sertraline hydrochloride	4
sertraline hcl 50mg	sertraline hydrochloride	4
atenix (sertraline)	atenolol	4
sertralina ratiopharm	sertraline hydrochloride	4
sertraline tab 100 mg	sertraline tab 100 mg	4
sertraline (setraline)	sertraline (setraline)	4
sertraline (g)	sertraline hydrochloride	4
novo-sertraline	novo-sertraline	4
sertraline 100mg	sertraline	4
zoloft/sertraline	zoloft/sertraline	4
sertraline/zoloft	sertraline	4
sertralin ranbaxy	sertraline hydrochloride	4
sertralin 100	sertraline hydrochloride	4
zoloft /sch/	sertraline	4
altisben	sertraline hydrochloride	4
torin	sertraline hydrochloride	4
sertraline generichealth 100 mg tablets	sertraline hydrochloride	4
sertraline tablet 50mg / brand name not specified	sertraline hydrochloride	4
selectra (sertraline hydrochloride)	sertraline hydrochloride	4
sertraline (zoloft) 50 mg tablet	sertraline	4
sertaline hydrochloride (sertraline hydrochloride)	hydrochloride	3
zoloft (sertraline hydrochloride)(sertraline hydrochloride)	zoloft (sertraline hydrochloride)(sertraline hydrochloride)	3
sertralina mylan generics 50 mg compresse rivestite con film	sertraline hydrochloride	3
zoloft (sertraline hydrochloride) (sertraline hydrochloride)	sertraline hcl; sertraline	3
sertraline (formulation unknown) (sertraline)	sertraline	3
sertraline hydrochloride tablets, 50 mg (base) (aellc) (sertraline)	sertraline	3
zoloft (sertraline hydrochloride) (tablets) (sertraline hydrochloride)	sertraline hcl; sertraline	3
sertraline tablet, 100 mg (milligram)	sertraline hydrochloride	3

sertraline hydrochloride tablets 25 mg	sertraline hydrochloride	3
sertraline hydrochloride tablets 100 mg	sertraline	3
zoloft (sertraline hydrochloride) ongoing	zoloft (sertraline hydrochloride) ongoing	3
sertralin mylan 100 mg filmdragera tablett	sertraline hydrochloride	3
zoloft (sertraline) (tablets) (sertraline)	sertraline	3
sertraline hydrochloride [d]	sertraline hydrochloride	3
sertraline (ngx) (sertraline)	sertraline	3
sertraline capsule non-company	sertraline hydrochloride	3
sertralina mylan generics 50mg	sertraline hydrochloride	3
zoloft (sertraline) unspecified	sertraline	3
sertraline hydrochloride (con.)	sertraline hydrochloride (con.)	3
sertralina mylan generics 100mg	sertraline hydrochloride	3
sertraline(sertraline) (unknown)	sertraline(sertraline) (unknown)	3
blinded sertraline hydrochloride	sertraline hydrochloride	3
zolof (sertraline hydrochloride)	sertraline hcl	3
sertraline (sertraline) (tablets)	sertraline	3
sertraline camber pharmaceuticals	sertraline hydrochloride	3
zoloft (sertraline hydrohcloride)	sertraline	3
gladem (sertraline hydrochloride)	gladem (sertraline hydrochloride)	3
sertraline 100 mg tablet	sertraline hydrochloride	3
auro-sertraline capsules	sertraline hydrochloride	3
sertraline 100mg tablets	sertraline	3
sertraline hcl greenstone	sertraline hcl greenstone	3
sertralin ratiopharm 50 mg	sertraline hydrochloride	3
apo-sertraline (sertraline)	apo-sertraline (sertraline)	3
sertraline (sertraline hcl)	sertraline hcl	3
chloridrate sertraline	chloridrate sertraline	3
sertraline 150mg daily	sertraline 150mg daily	3
sertraline, 100 mg	sertraline hydrochloride	3
sertralin-hormosan	sertraline hydrochloride	3
aremis (sertraline)	aremis (sertraline)	3
sertraline tab 50mg	sertraline tab 50mg	3
sertralin 1a pharma	sertraline hydrochloride	3
sertraline tab 25mg	sertraline tab 25mg	3
sertraline hcl 100 mg	sertraline hydrochloride	3
sertraline 25mg daily	sertraline 25mg daily	3
zoloft (pfizer)	sertraline	3
jzoloft od 50mg	sertraline hydrochloride	3
sertraline 75mg	sertraline hydrochloride	3

sertralina normon	sertraline hydrochloride	3
jzoloft 100mg	sertraline hydrochloride	3
zoloft 50mg	sertraline	3
serlof	sertraline hydrochloride	3
sertraline 50mg film?coated tablets	sertraline hydrochloride	3
sertraline hci sertraline	sertraline hydrochloride	3
sertraline 50 mg po daily	sertraline	3
sonalia	sertraline hydrochloride	3
sertraline 25mg tablet	sertraline hydrochloride	3
sertraline 50 mg tablet	sertraline	3
zoloft tab 50mg	sertraline	3
sertralin?1 a pharma 50 mg filmtabletten	sertraline hydrochloride	3
sertraline tablet 50mg / brand name not speci-fiedsertraline tablet.	sertraline	3
selectra [sertraline hydrochloride]	sertraline hcl	3
sertraline hcl oral tablet	sertraline	3
sertraline (sertraline) (unknown) (sertraline)	sertraline (sertraline) (unknown) (sertraline)	2
sertraline (sertraline) (tablets) (sertraline)	sertraline	2
sertraline hydrochloride/s,s-reboxetine succinate	sertraline hcl; succinate; reboxetine	2
zoloft (sertraline hydrochloride) (100 milligram)	sertraline	2
sertraline hcl (sertraline hcl) film-coated tablet	sertraline hcl	2
sertraline hco (sertraline) tape (including poul-tice)	sertraline	2
zoloft(sertraline hydrochloride)(sertraline hydro-chloride)	sertraline hcl; sertraline	2
sertraline (sertraline) (50 milligram, tablets) (sertraline)	sertraline	2
sertralin mylan 100 mg filmdragerde tabletter	sertraline hydrochloride	2
tresleen (sertraline hydrochloride) (tablets) (ser-traline hydrochlorid	sertraline; sertraline hcl	2
sertralina bexal 50 mg comprimidos recubiertos con pelicula efg , 30 c	sertraline hydrochloride	2
sertraline hydrochloride tablets, 100 mg (base) (aellc) (sertraline hy	sertraline hcl; sertraline	2
zoloft (sertraline hydrochloride) unspecified	sertraline	2
sertralina aurobindo compresse rivestite con film 50 mg (sertaline) film-coated	sertraline hydrochloride	2
zoloft (sertraline hydrlochloride)	sertraline	2
zoloft (generic) 50 mg cvs pharmacy	sertraline hydrochloride	2
sertraline (sertraline) unspecified	sertraline	2
sertraline hydrochloride tablets usp	sertraline hydrochloride	2
sertralin heumann 50 mg filmtabletten	sertraline hydrochloride	2

sertraline hydrochloride (sertraline)	sertraline hcl	2
sertraline hydrochloride tablets 50m g	sertraline hydrochloride	2
sertraline(sertraline)(sertraline)	sertraline	2
sertraline od (sertraline hydrochloride)	sertraline hydrochloride	2
sertraline (sertraline) (sertraline-hcl)	sertraline	2
sertraline (sertraline hcl) 100 mg	sertraline	2
setraline hydrochloride tabs 100mg	sertraline hydrochloride	2
jzoloft (sertraline hydrochloride) tablet	sertraline hcl	2
zoloft 50 mg compresse rivestite con film	sertraline	2
sertraline 50mg (sertaline) unknown, 50mg	sertraline hydrochloride	2
lustral (sertraline hydrochloride) tablet	sertraline	2
novo-sertraline (sertraline hydrochloride)	novo-sertraline (sertraline hydrochloride)	2
sertraline hcl 100 mg lupine pharmaceutica l	sertraline hydrochloride	2
sertraline hydrochloride (sertraline) tablet	sertraline hydrochloride	2
sertraline actavis 100 mg film-coated tablet	sertraline hydrochloride	2
sertraline (ngx)(sertraline)	sertraline	2
sertraline 100 mg green stone	sertraline	2
sertraline hcl 100 mg tablets	sertraline hydrochloride	2
sertraline (hydrochloride of)	sertraline hydrochloride	2
zoloft (sertraline) (unknown)	sertraline	2
sertraline mylan 25 mg, g?lule	sertraline hydrochloride	2
sertraline hydrochloride (ngx)	sertraline hcl	2
sertraline (sertraline) tablet	sertraline hydrochloride	2
sertralin actavis (sertraline)	sertraline	2
sertraline(sertraline)(unknown)	sertraline(sertraline)(unknown)	2
oralin film-coated tablets 50mg	sertraline hydrochloride	2
sertralina 50 mg 30 comprimidos	sertraline hydrochloride	2
sertraline (sertraline) (3.1 g)	sertraline	2
sertraline 50 mg (no pref. name)	sertraline	2
sertraline (sertraline) (100 mg)	sertraline	2
zoloft (sertraline hydrochlride)	sertraline	2
zoloft (sertraline hydochloride)	sertraline	2
zoloft (srtraline hydrochloride)	hydrochloride; sertraline	2
chloroquine/sertraline/famotidine	famotidine; chloroquine / sertraline	2
zoloft (sertraline hydorchloride)	zoloft (sertraline hydorchloride)	2
sertraline (manufacturer unknown)	sertraline (manufacturer unknown)	2
deprax [sertraline hydrochloride]	sertraline hydrochloride	2
zoloft (sertrlaine hydrochloride)	hydrochloride; sertraline	2
sertraline hci film-coated tablet	sertraline	2

zoloft (sertraline hdyrochloride)	sertraline	2
zoloft (sertralien hydrochloride)	hydrochloride; sertraline	2
sertraline hydrocloride	sertraline hydrochloride	2
sertraline, 50 mg lupin	sertraline hydrochloride	2
sertraline (non-abbvie)	sertraline (non-abbvie)	2
zoloft (sertraline hcl)	zoloft (sertraline hcl)	2
sertraline hcl (tablets)	sertraline hcl	2
sertraline hcl tabs 50mg	sertraline	2
sertraline 100 mg pfizer	sertraline	2
sertraline hdyrochloride	sertraline	2
sertraline 25 mg po daily	sertraline 25 mg po daily	2
sertraline 50mg od tablet	sertraline hydrochloride	2
sertraline 25mg od tablet	sertraline hydrochloride	2
sertraline hcl tablets	sertraline hydrochloride	2
zoloft/sertraline 25mg	zoloft/sertraline 25mg	2
sertraline hci tablets	sertraline	2
aurobindo (sertraline)	sertraline hydrochloride	2
sertraline concentrate	sertraline	2
sertraline (no pref. name)	sertraline	2
sertrline (sertraline)	sertraline	2
sertraline hcl (sertraline)	sertraline hcl	2
sertraline hcl 25 mg tablet	sertraline hydrochloride	2
sertraline (generic zoloft)	sertraline hydrochloride	2
sertralin dura (sertraline)	sertraline	2
sertaline (sertraline)	sertraline	2
sertraline ranbaxy	sertraline hydrochloride	2
generic for zoloft	sertraline	2
sertraline/generics	sertraline hydrochloride	2
sertralina winthrop	sertraline hydrochloride	2
sertraline bluefish	sertraline bluefish	2
sertraline milpharm	sertraline hydrochloride	2
sertraline chloride	sertraline	2
sertralin helvepharm	sertraline hydrochloride	2
sertraline tab 50 mg	sertraline tab 50 mg	2
sertraline tab 100mg	sertraline tab 100mg	2
sertralin sandoz eco	sertraline hydrochloride	2
sertaline hcl 100 mg	sertraline hydrochloride	2
sertraline cloridrato	sertraline	2
zoloft/sertraline hcl	sertraline hcl	2

sertraline 50 mg daily	sertraline 50 mg daily	2
ropinirole, sertraline	ropinirole, sertraline	2
sertraline hct	sertraline	2
act sertraline	sertraline hydrochloride	2
sertraline 10g	sertraline hydrochloride	2
jzoloft od 25mg	sertraline hydrochloride	2
sertraline barr	sertraline hydrochloride	2
ariale 50 mg tab	sertraline hydrochloride	2
mylan-sertraline	mylan-sertraline	2
ratio-sertraline	ratio-sertraline	2
sertraline 200mg	sertraline	2
sertraline hc	sertraline hc	2
co sertraline	co sertraline	2
sertraline ab	sertraline ab	2
sertraline (pill)	sertraline	2
sertraline 200 mg	sertraline 200 mg	2
zoloft sertraline	zoloft sertraline	2
zoloft hcl	sertraline	2
(zoloft)	sertraline	2
halea	sertraline hydrochloride	2
sertraline aurobindo film?coated tablets 50 mg	sertraline hydrochloride	2
zoloft 50 mg daily	sertraline	2
sertraline hexal	sertraline hydrochloride	2
sertralina sandoz	sertraline hydrochloride	2
serta/sertraline	sertraline	2
sertraline mylan 50 mg, g?lule	sertraline hydrochloride	2
sertraline generichealth 50 mg tablets	sertraline hydrochloride	2
sertraline capsule	sertraline hydrochloride	2
sertraline 50mg qd	sertraline	2
auro?sertraline capsules	sertraline hydrochloride	2
zoloft (setraline)	; sertraline	2
sertraline alter	sertraline hydrochloride	2
sertraline greenstone	sertraline	2
sertraline hydrochloride 50mg	sertraline	2
serta [sertraline]	sertraline	2
zoloft 100mg daily	sertraline	2
sertraline 25mg tablets	sertraline	2
senta [sertraline hydrochloride]	sertraline hcl	2
zoloft 100mg tablet	sertraline	2

sertraline (misol)	sertraline	2
sertraline h tab 50mg	sertraline	2
sertraline hcl oral tablet 100 mg	sertraline	2
zoloft tablet 25mg	sertraline	2
sertraline hcl tabs 100 mg; generic for zoloft	sertraline hydrochloride	1
sertraline (unknown)(sertraline hydrochloride)	sertraline hcl; sertraline	1
sertraline (strength and manufacturer unknown)	sertraline	1
besitran (besitran - sertraline hydrochloride)	sertraline hcl;	1
sertraline chloride (sertraline hydrochloride)	sertraline hcl; sertraline	1
sertraline aurobindo filmomhulde tabletten 50mg	sertraline hydrochloride	1
sertraline hydrochloride usp generic for zoloft	sertraline hydrochloride	1
sertraline (unknown) (sertraline hydrochloride)	sertraline hcl; sertraline	1
zoloft (sertraline hydrochloride) tablet, 100mg	sertraline	1
sertraline (sertraline) (capsules) (sertraline)	sertraline	1
sertraline (sertraline)(100 milligram, tablets)	sertraline	1
sertraline (ngx)(sertraline) film-coated tablet	sertraline	1
sertraline (sertraline) (50 milligram, tablets)	sertraline	1
zoloft (sertraline hydrochloride) tablet, 50 mg	sertraline	1
leflunomide, sertraline, azelastine, fluticasone	leflunomide, sertraline, azelastine, fluticasone	1
sertraline tablet 50mg tablet, 50 mg (milligram)	sertraline hydrochloride	1
zoloft (sertraline hydrochloride) tablet, 100 mg	sertraline	1
sertraline (sertraline) (sertraline, sertraline)	sertraline	1
zoloft (sertraline hysrochloride) (50 milligram)	sertraline	1
sertraline hcl(sertraline hydrochloride)(unknown)	sertraline hcl	1
sertraline hydrochloide (sertaline hydrochloride)	hydrochloride; sertraline	1
besitran (sertralinehydrochloride) tablet, 300 mg	besitran (sertralinehydrochloride) tablet, 300 mg	1
zoloft (tablets) sertraline hydrochloride tablets	sertraline hcl; sertraline	1
sertraline 100mg (invagen cipla) g 214 oval tablet	sertraline hydrochloride	1
sertraline tablet 100mg tablet, 100 mg (milli-gram)	sertraline hydrochloride	1
sertraline hcl (sertraline hydrochloride), ongoing	sertraline hcl (sertraline hydrochloride), ongoing	1
jzoloft (sertraline hydrochloride) tablet; unknown	jzoloft (sertraline hydrochloride) tablet; unknown	1
jzoloft (sertraline hydrochloride) (hydrochloride)	hydrochloride; sertraline hcl	1
sertraline hydrochloride(sertraline hydrochloride)	sertraline hcl	1
zoloft / (zoloft-sertraline hydrochloride-capsule)	sertraline	1
zoloft (sertraline hydrochloride) (12.5 milligram)	sertraline	1
setraline hydrochloride (sertraline hydrochloride)	hydrochloride;	1
sertraline hydrochloride (sertraline hydrochoride)	sertraline; sertraline hcl	1

setraline hydrochloride (sertraline hydrochloride0	sertraline; hydrochloride;	1
sertraline hyrochloride (sertraline hydrochloride)	sertraline hcl; sertraline	1
zoloft 50mg compresse rivestite con film/sertraline	zoloft 50mg compresse rivestite con film/sertraline	1
sertraline hcl (sertraline hydrochloride)(tablets)	sertraline hcl	1
sertraline hydrochloride 9sertraline hydrochloride)	hydrochloride; sertraline hcl	1
sertraline (sertraline) (50 milligram) (sertraline)	sertraline	1
sertralin 1a farma (ngx) (sertraline) unknown, 50mg	sertraline	1
sertraline hydrochloride (sertraline hydrochloride0	sertraline; sertraline hcl	1
sertralins hydrochloride (sertraline hydrochloride)	hydrochloride	1
sertraline hydrochloride) (sertraline hydrochloride)	sertraline hcl	1
zoloft (sertraline hydrochloride) film-coated tablet	sertraline	1
sertraline (sertraline) (100 milligram) (sertraline)	sertraline	1
sertraline (sertraline) tablet 09/15/2011 to ongoing	sertraline	1
glyburide, muscle relaxer, zoloft (sertraline) 50 mg	glibenclamide; sertraline	1
sertralin hexal (ngx)(sertraline) film-coated tablet	sertraline	1
sertraline hydrochlorid (sertraline hydrochloride) ta	sertraline hcl; sertraline	1
sertralina aurobindo compresse rivestite con film 50mg	sertraline hydrochloride	1
sertraline (ngx)(sertraline) film-coated tablet, 100mg	sertraline	1
sertraline hydrochlorothiazide (sertraline hydrochloride)	hydrochlorothiazide; sertraline	1
zoloft (unspecified) sertraline hydrochloride	sertraline hcl; sertraline	1
sertralina mylan 50 mg comprimidos revestidos por pel?cula	sertraline hydrochloride	1
zoloft (sertraline hydrohcloride) (sertaline hydrochloride)	hydrochloride; sertraline	1
sertraline (sertraline) (25 milligram, tablet) (sertraline)	sertraline	1
zoloft (sertraline hydrchloride) (sertraline hydrochloride)	sertraline hcl; sertraline	1
zoloft (sertraline hydrochloride) (sertraline hydrochlorde)	sertraline	1
zoloft (sertraline hydrochoride) (sertraline hydrochloride)	sertraline hcl; sertraline	1
zoloft (sertraline hydrochloride) (setraline hydro-	hydrochloride; ; sertraline	1

chloride)		
sertraline hydrochloride (sertraline hydrochloride) (50 mg)	sertraline	1
zoloft (dertraline hydrochloride) (sertraline hydrochloride)	sertraline hcl; hydrochloride; sertraline	1
sertraline film-coated tablet (sertaline) film-coated tablet	sertraline hydrochloride	1
sertraline hydrochloride tablets, 100mg (atllc) (sertraline)	sertraline hcl; sertraline	1
assert (sertraline hydrochloride) (sertraline hydrochloride)	sertraline hcl	1
sertralina mylan generics 100 mg compresse rivestite con film	sertraline hydrochloride	1
jzoloft (sertraline hydrochloride) (sertraline hydrochloride)	sertraline hcl	1
zoloft (sertraline hydrochloride) (sertraline hiydrochloride)	sertraline	1
serbtraline hydrochloride (sertraline hydrochloride) (unknown)	serbtraline hydrochloride (sertraline hydrochloride) (unknown)	1
sertralina mylan 50 mg comprimidos recubiertos con pel?cula efg	sertraline hydrochloride	1
sertraline 50mg-generic for }zoloft for 9 days than 100mg 5days	sertraline	1
sertraline hcl(sertraline hydrochloride)(50 milligram, tablets)	sertraline	1
sertraline hydrochloride tablets 100 mg (sertraline tablets 100	sertraline; sertraline hcl	1
sertraline (sertraline hydrochloride) (sertraline hydrochloride)	sertraline (sertraline hydrochloride) (sertraline hydrochloride)	1
sertraline hydrochloride (sertraline hydrochloride) per oral nos	sertraline hcl	1
sertraline hydrochloride (unknown) (sertraline hydrochloride) unk	sertraline hcl	1
zoloft - 50 mg compresse rivestite con film - pfizer italia s.r.l.	sertraline	1
sertraline hydrochloride (sertraline hydrochloride) (50 milligram)	sertraline	1
sertraline hcl tab 100mg side 1: 100 mg; side 2: g 4910 greenstone	sertraline	1
zoloft (sertraline hydrochloride)(sertraline hydrochloride) tablet	sertraline hcl; sertraline	1
sertraline hydrochloride (sertraline hydrochloride) 50 mg; unknown	sertraline	1
sertraline hcl (sertraline hydrochloride) (100 milligram, tablets)	sertraline	1
sertraline hydrochloride tablets, 100 mg (base) (aelc) (sertraline	sertraline hcl; sertraline	1

sertraline hydrochloride tablets, 50 mg (base) (allec) (sertraline)	sertraline hcl; sertraline	1
sertraline hydrochloride tablets, 25 mg (base) (aellc) (sertraline)	sertraline hcl; sertraline	1
zoloft (sertraline hydrochloride) (sertraline hy-drochloride) tablet	sertraline hcl; sertraline	1
sertraline hydrochloride 100mg tablet manufac-turer: camber pharmacy	sertraline	1
zoloft (sertraline hydrochlorie) (unknown) (ser-traline hydrochloride)	zoloft (sertraline hydrochlorie) (unknown) (ser-traline hydrochloride)	1
zoloft (sertraline hydrochloride) (unknown) (sertraline hydrochloride)	zoloft (sertraline hydrochloride) (unknown) (sertraline hydrochloride)	1
sertraline (sertraline hydrochloride) (unknown) (sertraline hydrochlor	sertraline; sertraline hcl	1
zoloft (sertralien hydrochloride) (100 milligram) (sertraline hydrochl	sertraline; hydrochloride	1
sertraline hyrochloride (formulation unknown) (sertraline hydrochlorid	sertraline hyrochloride (formulation unknown) (sertraline hydrochlorid	1
sertraline hydrochloride (watson laborato-ries)(sertraline hydrochlorid	sertraline; sertraline hcl	1
sertraline hydrochloride (unknown) (sertraline hydrochoride) unk,unk,unk	sertraline; sertraline hcl	1
sertraline hydrochloride (unknown) (sertraline hydrochloride) unk, unkunk	sertraline hcl	1
sertraline hydrochloride (25 milligram, tablet) (sertraline hydrochloride)	sertraline; sertraline hcl	1
zoloft (sertraline hydrochloride)(50 milligram) (sertraline hydrochloride)	sertraline	1
sertraline hydrochloride (unknown) (sertraline hydrochloride) unk, unk unk	sertraline hcl	1
zoloft (sertraline hydrochloride) (50 milligram) (sertraline hydrochloride)	sertraline	1
sertraline hydrochloride(sertraline hydrochlo-ride)(sertraline hydrochloride)	sertraline hcl	1
zoloft (sertraline hydrochloride) (100 milligram, tablets) (sertraline hydrochlo	sertraline	1
zoloft(sertraline hydrochloride) (50 milligram, tablet) (sertraline hydrochlorid	sertraline	1
sertraline orion film-coated tablet	sertraline hydrochloride	1
sertraline 25 mg peg every 24 hours	sertraline 25 mg peg every 24 hours	1
sertralin beta 100 mg filmtabletten	sertraline hydrochloride	1
sertraline hydrochloride 7405288149	sertraline hydrochloride 7405288149	1
sertraline/manufacturer unspecified	sertraline/manufacturer unspecified	1
sertralin apotex potahovan? tablety	sertraline hydrochloride	1
resteral (sertraline hydrochloride)	resteral (sertraline hydrochloride)	1
sertraline 50mg film-coated tablets	sertraline hydrochloride	1

zolpidem (sertraline hydrochloride)	zolpidem	1
zoloft 100 mg tabs (sertraline hcl)	sertraline	1
jzoloft (sertraline hydrochloride)	sertraline hcl	1
sertraline hcl 100 mg 31722-0214-05	sertraline	1
sertraline (sertraline)(sertraline)	sertraline	1
altisben (sertraline hydrochloride)	sertraline hcl	1
sertraline (ngx) (sertraline) 25 mg	sertraline	1
decadron (sertraline hydrochloride)	dexamethasone	1
zoloft (sertraline hydrochloride) c	sertraline	1
sertraline (sertraline hcl 25mg tab)	sertraline hydrochloride	1
sertraline (sertraline hcl 50mg tab)	sertraline hydrochloride	1
sertraline film?coated tablets 100mg	sertraline hydrochloride	1
sertraline film?coated tablets 50 mg	sertraline hydrochloride	1
sertraline hydrochloride 25mg tablet	sertraline hydrochloride	1
sertraline hcl physicians total care	sertraline hydrochloride	1
sertraline (sertraline) (sertarline)	sertraline (sertraline) (sertarline)	1
5)?zoloft (sertraline hydrochloride)	5)?zoloft (sertraline hydrochloride)	1
perphenazine, quetiapine, sertraline	perphenazine; quetiapine; sertraline	1
sertraline hydrochloride concentrate	sertraline hcl	1
setralina (sertraline hydrochloride)	sertraline hcl	1
sertraline hydrochloride 50 mg mylan	sertraline	1
setraline (sertraline hydrochloride)	setraline (sertraline hydrochloride)	1
sertralin (sertraline hydrochloride)	sertraline hcl	1
sertraline 200 mg daily renewed 5/04	sertraline	1
synthroid (sertraline hydrochloride)	levothyroxine	1
sertralina [sertraline hydrochloride]	sertralina [sertraline hydrochloride]	1
sertraline, 50 mg magnet labs pvt ltd	sertraline hydrochloride	1
sertraline hcl 25 mg northstar rx llc	sertraline hydrochloride	1
sertraline hcl 25 mg / zoloft generic	sertraline hydrochloride	1
sertraline hydrochloride 50mg walmart	sertraline hydrochloride	1
zoloft (sertraline hydrochloride) unk	sertraline	1
clonazepam (sertraline hydrochloride)	clonazepam	1
sertraline hcl, 100 mg lupin pharmaceu	sertraline hydrochloride	1
weellbutrin, sertraline, mv, vitamin d	weellbutrin, sertraline, mv, vitamin d	1
zoloft/usa/ (sertraline hydrochloride)	sertraline hcl; sertraline	1
sertraline 100 mg/once daily northstar	sertraline	1
zoloft (sertraline hydrochloride) 50mg	zoloft (sertraline hydrochloride) 50mg	1
sertraline hcl 25mg generic for zoloft	sertraline; sertraline hcl	1
lustral/ire/(sertraline hydrochloride)	sertraline hcl; sertraline	1

aremis (sertraline hydrochloride, , 0)	sertraline hcl;	1
sertraline hcl 100 mg greenstone brand	sertraline	1
sertralin aurobindo filmtabletten 100mg	sertraline hydrochloride	1
sertraline hcl sertraline hydrochloride	sertraline hcl sertraline hydrochloride	1
xanax, tagamet, benadryl, paxil, zoloft	alprazolam; cimetidine; diphenhydramine; par-oxetine; sertraline	1
sertraline hydrochloride (mfr: unknown)	sertraline hcl	1
zoloft / sertraline 150mg pfizer dail\y	sertraline	1
sertraline hcl (sertraline hcl) unknown	sertraline hcl	1
zoloft /usa/ (sertraline hdyrochloride)	sertraline	1
sertraline (50 mg, tablet) (sertraline)	sertraline	1
sertraline hcl 100mg kaiser pharmacy nw	sertraline	1
gladem /sch/ (sertraline hydrochloride)	sertraline hcl;	1
ratio-sertraline 50mg zehrs drug stores	ratio-sertraline 50mg zehrs drug stores	1
zoloft (sertraline hydrochlioride)	sertraline	1
zolofrt (sertraline hydrochloride)	sertraline hcl	1
sertraline hcl 50 mg tablet (sertraline)	sertraline hydrochloride	1
zoloft (swertraline hydrochloride)	hydrochloride; sertraline	1
sertraline teva 50 mg film coated tablet	sertraline hydrochloride	1
sertraline eg (sertraline hydrochloride)	sertraline eg (sertraline hydrochloride)	1
sertraline - generic version 25mg camber	sertraline	1
sertralone hcl (sertraline) (sertraline)	sertraline	1
sertraline hcl (setraline hydrochloride)	sertraline hcl (setraline hydrochloride)	1
sertraline hcl(sertraline hydrochloride)	sertraline hcl(sertraline hydrochloride)	1
lustral (sertraline hydrochloride) /ire/	sertraline	1
sertraline hcl (sertraline hyrochloride)	sertraline; sertraline hcl	1
zoloft (sertraline hydrochloride) (pill)	sertraline	1
sertraline hydrochloride tablets (50 mg)	sertraline	1
zoloft (sertraline hydrochlodride)	sertraline	1
sertraline orion film-coated tablets 50mg	sertraline hydrochloride	1
zoloft (sertrailine hydrochloride)	hydrochloride; sertraline	1
sertraline, 50mg, generic for zoloft 50mg	sertraline hydrochloride	1
sertraline 25 mg tablet,kaiser permanente	sertraline hydrochloride	1
sertraline 100mg tablets, 100mg aurobindo	sertraline hydrochloride	1
zoloft (sertraline hydrochloiride) tablet	sertraline	1
generic zoloft (sertraline hydrochloride)	generic zoloft (sertraline hydrochloride)	1
sertraline (zoloft) 100 mg lupin pharmace	sertraline	1
zoloft(sertraline hydrochloride)(tablets)	sertraline	1
sertraline hcl (sertarline hydrochloride)	hydrochloride; sertraline hcl	1

zoloft(sertraline hydrochloride)(unknown)	sertraline	1
lustral(sertraline hydrochloride) unknown	sertraline	1
zoloft (unknown) sertraline hydrochloride	sertraline hcl; sertraline	1
zoloft (sertraline hydrochloride), 100 mg	sertraline	1
besitran 50 mg (sertraline hydrochloride)	sertraline;	1
desmethylsertraline (desmethylsertraline)	desmethylsertraline (desmethylsertraline)	1
apo-sertraline (sertraline hydrochloride)	apo-sertraline (sertraline hydrochloride)	1
sertraline hydrochloride tablets usp 25 mg	sertraline hydrochloride	1
sertraline, 25mg american health packaging	sertraline hydrochloride	1
sertraline hcl (sertaraline hydrochloride0	sertraline hcl (sertaraline hydrochloride0	1
sertralin mylan 50 mg filmdragerad tablett	sertraline hydrochloride	1
sertralin teva 100 mg filmdragerad tablett	sertraline hydrochloride	1
sertraline setraline 100mg northstar rx ll	sertraline hydrochloride	1
zoloft(sertraline hydrochloride) (unknown)	zoloft(sertraline hydrochloride) (unknown)	1
sertraline hcl (sertraline hydrlochloride)	sertraline hcl (sertraline hydrlochloride)	1
sertraline hcl tab 100mg generic for zoloft	sertraline hydrochloride	1
lustral (sertraline hydrochloride) (tablet)	lustral (sertraline hydrochloride) (tablet)	1
sertraline hydrochloride (sertraline hydroc	sertraline; sertraline hcl	1
sertraline hydrochloride (ngx) (sertraline)	sertraline; sertraline hcl	1
sertraline (sertraline hydrochloride) tablet	sertraline (sertraline hydrochloride) tablet	1
sertralin actavis 50 mg filmdragerad tablett	sertraline hydrochloride	1
zoloft (seertraline hydrochloride) (unknown)	hydrochloride; sertraline	1
sertralin actavis (sertraline hydrochloride)	sertralin actavis (sertraline hydrochloride)	1
sertraline (formulation unkown) (sertraline)	sertraline	1
sertraline ranbaxy film coated tablets 100mg	sertraline	1
zoloft (sertaline hydrochloride) unspecified	hydrochloride; sertraline	1
zoloft (capsules) (sertraline hydrochloride)	sertraline	1
sertraline generic: 20 mg/ml - generic tier 2	sertraline hydrochloride	1
sertraline tablets, usp 25mg ndc 76282-212-90	sertraline hydrochloride	1
sertraline 100 mg tab, 100 mg publix pharmacy	sertraline hydrochloride	1
sertraline hydrochloride tablet s usp, 100 mg	sertraline hydrochloride	1
sertraline - generic for zoloft 50 mg tablets	sertraline hydrochloride	1
zoloft - generic sertraline pizer? greenstone	sertraline	1
sertraline (sertraline) (tablet) (sertraline)	sertraline	1
sertraline (sertraline) (ta) (sertraline-hcl)	sertraline	1
sertraline hcl 50 mg tablets	sertraline hydrochloride	1
dr reddys labs uk sertraline	dr reddys labs uk sertraline	1
sertraline (sertraline) 25mg	sertraline	1
sertraline started on 100 mg	sertraline	1

sertraline (sertraline) (nr)	sertraline	1
zoloft (therapy unspecified)	sertraline	1
zoloft (quetiapine fumarate)	sertraline	1
sertraline hydrochlorid 100mg	sertraline hydrochlorid 100mg	1
sertralin 50 mg filmtabletten	sertraline hydrochloride	1
sertraline generic for zoloft	sertraline generic for zoloft	1
sertraline hcl 50 mg tab nort	sertraline hydrochloride	1
sertraline teva 50 mg, g?lule	sertraline	1
sertralin pfizer (sertraline)	sertralin pfizer (sertraline)	1
sertraline hcl 50mg aurobindo	sertraline hydrochloride	1
sertraline tablets, usp 100mg	sertraline	1
sertraline film-coated tablets	sertraline hydrochloride	1
sertraline lupin healthcare uk	sertraline hydrochloride	1
aurobindo mfg sertraline 100mg	sertraline hydrochloride	1
sertraline tablets, usp, 25 mg	sertraline hydrochloride	1
sertralin (trade name unknown)	sertraline hydrochloride	1
sertraline 50mg 2 pills 1x day	sertraline hydrochloride	1
sertraline, toprol xl, tylenol	sertraline, toprol xl, tylenol	1
sertraline mylan 25 mg, gelule	sertraline hydrochloride	1
sertraline hcl 100mg aurobindo	sertraline	1
sertraline, 100 mg, greenstone	sertraline	1
sertraline/desmethylsertraline	sertraline	1
sertraline (sertraline) talbet	sertraline	1
sertraline (sertraline), 50 mg	sertraline	1
sertraline 100 mgs. - westward	sertraline	1
sertraline 100 mgs - northstar	sertraline	1
sertraline tablets (wockhardt)	sertraline	1
apo-sertraline (no pref. name)	apo-sertraline (no pref. name)	1
sertraline hydrochloride 50 mg	sertraline	1
sertraline (sertraline) (con.)	sertraline	1
sertraline family (sertraline)	sertraline	1
topalgic (tramadol sertraline)	sertraline; tramadol;	1
zoloft 100 mg unknown- generic	sertraline	1
sertraline (ngx) (sertraline)	sertraline	1
sertraline (generic for zoloft)	sertraline (generic for zoloft)	1
sertraline tab sodium chlor neb	sertraline tab sodium chlor neb	1
sertraline sub for zoloft 50 mg	sertraline hydrochloride	1
sertraline 50mg 1 tablet po qhs	sertraline 50mg 1 tablet po qhs	1
sertraline 50 mg 1 tablet daily	sertraline 50 mg 1 tablet daily	1

sertraline 20 mg/ml concentrate	sertraline hydrochloride	1
sertal/sertraline hydrochloride	sertal/sertraline hydrochloride	1
serdep/sertraline hydrochloride	serdep/sertraline hydrochloride	1
sertraline (sertraline) (50mcg)	sertraline	1
sertraline 50 mg (mfr: unknown)	sertraline	1
sertraline -generic for zoloft-	sertraline	1
sertraline(unknown)(sertraline)	sertraline	1
sertraline hcl 100 mg northstar	sertraline	1
zolof (sertraline) (sertraline)	sertraline	1
loft (sertraline hydrochloride)	sertraline hcl	1
sertraline hcl 100 mg teva, usa	sertraline	1
sertraline (sertraline) (50 mg)	sertraline	1
sertraline (20 mg) (sertraline)	sertraline	1
zoloft (sertraline hydrocodone)	hydrocodone; sertraline	1
zoloft(sertraline hydrochloril)	sertraline	1
zolf (sertraline hydrochloride)	sertraline hcl	1
zoloft (sertaline hydrochoride)	sertraline	1
sertraline hcl oral tablet 100mg	sertraline hcl oral tablet 100mg	1
sertraline hydrochloride 10mg/ml	sertraline hydrochloride	1
sertraline 100 mg tablet, 100 mg	sertraline hydrochloride	1
sertraline hcl 25mg, 25 mg lupin	sertraline hydrochloride	1
sertralin-hormosan filmtabletten	sertraline hydrochloride	1
sertraline hcl 100 mg 90 tablets	sertraline hydrochloride	1
zolft (sertraline hydrochloride)	sertraline hcl	1
sertralina teva pharma bv 100 mg	sertraline hydrochloride	1
sertraline, 50 mg (mfr: unknown)	sertraline	1
zooft (sertraline hydrochloride)	sertraline hcl	1
sertraline (sertaline) (unknown)	sertraline	1
zocor (sertraline hydrochloride)	simvastatin	1
zoloft (sertraline hydrochlorie)	sertraline	1
sertraline (sertraline) 100mg hs	sertraline	1
zoloft (sertraline hdyrchloride)	sertraline	1
zoloft (sertraline hydrochoride)	sertraline	1
oloft (sertraline hydrochloride)	sertraline hcl	1
zoloft (sertraline hdrochloride)	sertraline	1
zoloft 50 mgs pfizer / walgreens	sertraline	1
sertraline hcl tablets 50 mg	sertraline hydrochloride	1
sertraline biogaran 50 mg, gelule	sertraline hydrochloride	1
serdep [sertraline hydrochloride]	serdep [sertraline hydrochloride]	1

aremis [sertraline hydrochloride]	aremis [sertraline hydrochloride]	1
sertraline biogaran 50 mg, g?lule	sertraline hydrochloride	1
sertraline hcl cf 100mg tabletten	sertraline hydrochloride	1
jzoloft (sertraline hydrocloride)	sertraline	1
zoloft generic brand 100 mg daily	sertraline hydrochloride	1
zoloft (sertraline hydrochlorine)	sertraline	1
sertraline hci (sertraline) 50 mg	sertraline hydrochloride	1
zoloft - generic not brand - name	sertraline hydrochloride	1
generic zoloft (for twelve years)	sertraline	1
sertraline (unknown) (sertrailne)	sertraline	1
sertraline hydrochloride tablets,	sertraline hcl	1
zoloft (dertraline hydrochloride)	hydrochloride; sertraline	1
sertraline generic form of zoloft	sertraline	1
sertraline hcl (zoloft) (unknown)	sertraline hcl (zoloft) (unknown)	1
zoloft (sertraline hyodrhcloride0	sertraline	1
jzoloft (sertraline hydrochloride	sertraline hcl	1
zoloft (sertarline hydrochloride)	hydrochloride; sertraline	1
zolort (sertraline hydrochloride)	sertraline hcl	1
zolofr (sertraline hydrochloride)	sertraline hcl	1
zoloft (sertralinr hydrochloride)	hydrochloride; sertraline	1
zoloft (sertraline eydrochloride)	sertraline	1
zolfot (sertraline hydrochloride)	sertraline hcl	1
zoloft (sertraline hydrcohloride)	sertraline	1
aranes (sertraline hydrochloride)	sertraline hcl	1
zoloft (sertraline hydeochloride)	sertraline	1
sertraline (lexapro)(zoloft)	sertraline (lexapro)(zoloft)	1
sertrailine oral concentrate	sertraline hydrochloride	1
omeprazole, bumetanide, sertraline	omeprazole, bumetanide, sertraline	1
atralin [sertraline hydrochloride]	atralin [sertraline hydrochloride]	1
sertraline (unknown) (sertraline)?	sertraline	1
sertraline hci (sertraline) 100 mg	sertraline hydrochloride	1
zoloft (sertraline hydrochloride)?	zoloft (sertraline hydrochloride)?	1
zoloft (sertraline hydrochloride)	sertraline	1
probably zoloft - maybe sertraline	sertraline	1
serolux (ngx) (sertraline) unknown	sertraline	1
sertraline hcl/ sertraline/ zoloft	sertraline; sertraline hcl	1
zoloft (serteraline hydrochloride)	hydrochloride; sertraline	1
sertraline hydrochloride (tablets)	sertraline hcl	1
sertraline (50 milligram, tablets)	sertraline	1

zoloft (seritraline hydrochloride)	hydrochloride; sertraline	1
zoloft (ser4traline hydrochloride)	hydrochloride; sertraline	1
zoloft (sertraline hydrodhcloride)	sertraline	1
zoloft (sertrelaine hydrochloride)	hydrochloride; sertraline	1
zoloft (serttraline hydrochloride)	hydrochloride; sertraline	1
zoloft (sertralinie hydrochloride)	hydrochloride; sertraline	1
zoloft (sertraliine hydrochloride)	hydrochloride; sertraline	1
zoloft (sertyraline hydrochloride)	hydrochloride; sertraline	1
sertraline (greenstone)	sertraline (greenstone)	1
sertraline 150 mg daily	sertraline 150 mg daily	1
sertraline 100 mg daily	sertraline 100 mg daily	1
sertraline, spironolact	sertraline, spironolact	1
sertraline chlorhydrate	sertraline chlorhydrate	1
sertraline (stimuloton)	sertraline (stimuloton)	1
sertraline 200mg tablet	sertraline 200mg tablet	1
sertraline hcl 50mg tab	sertraline hcl 50mg tab	1
sertraline tablet 100mg	sertraline tablet 100mg	1
sertraline hcl -zoloft-	sertraline; sertraline hcl	1
sertraline (non-abbott)	sertraline	1
sertraline (sertarline)	sertraline	1
certraline (sertraline)	sertraline	1
sertraline (sertaline)	sertraline	1
gladem (sertraline hcl)	gladem (sertraline hcl)	1
sertraline 100mg po q24h	sertraline 100mg po q24h	1
sertraline, 100 mg lupin	sertraline hydrochloride	1
methotrexate, sertraline	methotrexate, sertraline	1
sertraline actavis group	sertraline actavis group	1
sertraline 50mg po daily	sertraline 50mg po daily	1
sertraline (sertaraline)	sertraline (sertaraline)	1
sertrtaline (sertraline)	sertraline	1
sertraliine (sertraline)	sertraline	1
serttraline (sertraline)	sertraline	1
sertraline hyrdochloride	sertraline	1
sertraline (isertraline)	sertraline	1
sertraline hcl 100mg tabs	sertraline hydrochloride	1
sertraline 100 mb tablets	sertraline hydrochloride	1
sertraline 200 codydramol	sertraline 200 codydramol	1
sertraline/sertraline hcl	sertraline hydrochloride	1
sertraline - (neoquimica)	sertraline hydrochloride	1

sertralini hydrochloridum	sertraline hydrochloride	1
sertraline hydrochlorixde	sertraline hydrochloride	1
sertraline 20mg/ml apotex	sertraline	1
zoloft 100 mg *nm* tablet	sertraline	1
sertraline (mfr: unknown)	sertraline	1
antidepressant sertraline	sertraline; antidepressant	1
zoloft /usa/ (sertraline)	sertraline	1
transdermal system zoloft	sertraline	1
sertraline (75 milligram)	sertraline	1
sertraline 100 mg po daily	sertraline hydrochloride	1
sertraline (hydrochloride)	sertraline hydrochloride	1
sertraline hcl 50 mg po hs	sertraline hcl 50 mg po hs	1
sertraline (chlorohydrate)	sertraline hydrochloride	1
sertraline 100mg aurobindo	sertraline hydrochloride	1
sertraline, 25 mg greensto	sertraline hydrochloride	1
zoloft tablet (sertraline)	zoloft tablet (sertraline)	1
sertraline 50 mg tabletten	sertraline hydrochloride	1
sertraline, generic zoloft	sertraline, generic zoloft	1
sertraline hcl 25mg tablet	sertraline hcl 25mg tablet	1
sertraline(ngx)(sertraline	sertraline	1
sertraline hc (sertraline)	sertraline	1
sertraline hydrochloride t	sertraline hcl	1
sertraline hydrochlororide	sertraline	1
sertraline 50 mg 30 tablets	sertraline 50 mg 30 tablets	1
sertraline hcl 50 mg tablet	sertraline hydrochloride	1
sertraline 200mg once daily	sertraline 200mg once daily	1
generic zoloft (sertraline)	sertraline hydrochloride	1
sertraline hcl 100 (zoloft)	sertraline hcl 100 (zoloft)	1
sertraline 100 mg northstar	sertraline	1
zoloft (sertraline) unknown	sertraline	1
zoloft 75 milligrams pfizer	sertraline	1
sertraline hcl (zoloft) tab	sertraline	1
eleva (sertraline)	sertraline	1
sertraline unknown	sertraline	1
daxid (sertraline)	sertraline	1
zoloft (steraline)	sertraline	1
blinded sertraline	sertraline	1
sertraline cinfa g	sertraline	1
sertraline 50mg va	sertraline	1

lustral 100 pfizer	sertraline	1
sertraline, 50mg an	sertraline hydrochloride	1
sertraline cristers	sertraline cristers	1
sertraline (aremis)	sertraline (aremis)	1
sertraline(lustral)	sertraline(lustral)	1
desmethylsertraline	desmethylsertraline	1
sertalin ratiopharm	sertraline hydrochloride	1
sertraline {zoloft}	sertraline	1
sertraline - zoloft	sertraline	1
sertraline (luxeta)	sertraline	1
zoloft / 010011402/	sertraline	1
zoloft (sretraline)	sertraline	1
zoloft (interferon)	sertraline	1
sertralina aurovitas	sertraline hydrochloride	1
sertralin ratiopharm	sertraline hydrochloride	1
serlain [sertraline]	serlain [sertraline]	1
sertraline 150mg tab	sertraline 150mg tab	1
zoloft generic 50 mg	sertraline hydrochloride	1
zoloft (sertraline)	zoloft (sertraline)	1
sertraline (zoloft)	sertraline (zoloft)	1
zoloft 50 mg capsule	sertraline	1
sertraline hydroxide	sertraline hydroxide	1
sertraline 100 lupin	sertraline	1
zoloft (sertratline)	sertraline	1
zoloft (sertralinei)	sertraline	1
zoloft, pfizer 25 mg	sertraline	1
sertraline cefazolin	cefazolin; sertraline	1
zoloft (sertraliine)	sertraline	1
sertraline 150 mg qd	sertraline	1
any generic zoloft	sertraline hydrochloride	1
serta (sertraline)	sertraline	1
sertraline hcl accord	sertraline hydrochloride	1
zoloft (generic form)	sertraline hydrochloride	1
ranbaxy uk sertraline	sertraline hydrochloride	1
sertraline, 100 mg mg	sertraline hydrochloride	1
generic zoloft, 100mg	sertraline hydrochloride	1
sertraline hcl zoloft	sertraline hcl zoloft	1
sertraline ssri 100mg	sertraline hydrochloride	1
sertraline/alprazolam	sertraline/alprazolam	1

zolpidem (sertraline)	zolpidem	1
sertraline 50mg mylan	sertraline	1
insertec (sertraline)	sertraline	1
besitran (sertraline)	besitran (sertraline)	1
zoloft (100 miligram)	sertraline	1
sertra-q (sertraline)	sertraline	1
lustral (sertraline)	sertraline	1
zoloft (singulair)	sertraline	1
dyna sertraline tablet	sertraline hydrochloride	1
sertraline, 25mg, 50mg	sertraline, 25mg, 50mg	1
sertraline 200mg po qd	sertraline 200mg po qd	1
sertraline 100mg daily	sertraline 100mg daily	1
sertraline/hydrocodone	sertraline/hydrocodone	1
sertraline / synthroid	sertraline / synthroid	1
sertraline hcl bacofen	sertraline hcl	1
sertraline hcl/ zoloft	sertraline hcl/ zoloft	1
setriline (sertraline)	sertraline	1
sertraline (sertraline	sertraline	1
sertraline non bi drug	sertraline	1
sertralne (sertraline)	sertralne (sertraline)	1
sertaline 50mg	sertraline hydrochloride	1
sertraline prn	sertraline prn	1
sertralin base	sertraline hydrochloride	1
apo?sertraline	sertraline hydrochloride	1
sertraline hc1	sertraline hc1	1
sertraline ncl	sertraline	1
zoloft pfize5r	sertraline	1
dyna sertraline	dyna sertraline	1
sertraline auro	sertraline hydrochloride	1
auro-sertraline	sertraline hydrochloride	1
sertalaline hcl	sertraline hydrochloride	1
mint-sertraline	sertraline hydrochloride	1
oxynorm lustral	sertraline; oxycodone	1
sertraline 150mg	sertraline hydrochloride	1
sivem-sertraline	sivem-sertraline	1
sertraline - 100	sertraline - 100	1
sertraline 50mg.	sertraline 50mg.	1
setraline hcl 50	sertraline hydrochloride	1
sertraline 25 mg	sertraline 25 mg	1

200mg sertraline	200mg sertraline	1
sertraline almus	sertraline almus	1
sertraline zydus	sertraline	1
nadsertralinehcl	nadsertralinehcl	1
sertraline 75 mg	sertraline 75 mg	1
arrow-sertraline	sertraline hydrochloride	1
sertraline lupin	sertraline hydrochloride	1
zoloft 100mg/day	sertraline	1
zoloft - generic	sertraline	1
zoloft 200mg/day	sertraline	1
sertraline 150mgs	sertraline hydrochloride	1
sertraline neurax	sertraline neurax	1
sertraline acd us	sertraline hydrochloride	1
sertraline nisone	sertraline nisone	1
ic sertraline hci	ic sertraline hci	1
sertraline-dp 100	sertraline-dp 100	1
sertraline- ciplo	sertraline- ciplo	1
sertralina pfizer	sertraline hydrochloride	1
sertralina 100 mg	sertraline hydrochloride	1
sertraline 150 mg	sertraline hydrochloride	1
sertraline liquid	sertraline liquid	1
sertraline zoloft	sertraline	1
sertraline (none)	sertraline	1
zoloft v. placebo	sertraline	1
sertraline effer-k	sertraline effer-k	1
sertraline teva uk	sertraline hydrochloride	1
accords sertraline	sertraline hydrochloride	1
sertraline(zoloft)	sertraline(zoloft)	1
sertraline hcl ssd	sertraline hcl ssd	1
generic sertraline	generic sertraline	1
zoloft(sertraline)	zoloft(sertraline)	1
sertralin winthrop	sertraline hydrochloride	1
zoloft 50g	sertraline	1
gen zoloft	sertraline	1
oralin 50mg	sertraline hydrochloride	1
sertralin e	sertraline hydrochloride	1
*sertraline	*sertraline	1
sertraline,	sertraline	1
sertraline)	sertraline	1

zoloft ssri	ssri; sertraline	1
sertralin ct	sertraline hydrochloride	1
zoloft 100mg	sertraline	1
(sertraline)	sertraline	1
lasertraline	lasertraline	1
sertraline od	sertraline od	1
sertralina 50	sertraline hydrochloride	1
sertraline xl	sertraline	1
sertraline 50	sertraline	1
aserta	sertraline hydrochloride	1
zert-od	sertraline hydrochloride	1
concorz	sertraline hydrochloride	1
restaline	sertraline hydrochloride	1
serdep (sertraline)	sertraline hydrochloride	1
zoloft (sertraline hydrochlo-ride)(unknown)(sertraline hydrochloride)	sertraline hcl; sertraline	1
sertraline potassium	potassium; sertraline	1
sertraline orion film?coated tablets 50mg	sertraline hydrochloride	1
zoloft??	sertraline	1
sertraline.		1
deprax (sertraline hydrochloride)	sertraline hydrochloride	1
sertralin puren 100 mg film coated tablets	sertraline hydrochloride	1
sertraline 50 mg qday	sertraline	1
sertraline 10 + 20 mg	sertraline hydrochloride	1
zoloft 100mcg	sertraline	1
milpharm sertraline	sertraline hydrochloride	1
sertraline hcl oral	sertraline hcl	1
generic zoloft 75mg	sertraline	1
sertraline 100 mg film coated tablets	sertraline hydrochloride	1
zoloft 50mg oral	sertraline	1
sertraline hcl 100mg, oral	sertraline	1
sertraline hcl 50mg, oral	sertraline	1
sertraline 25mg tab	sertraline	1
leyuan	sertraline hydrochloride	1
sertraline np	sertraline	1
sertraline sun	sertraline hydrochloride	1
pantoprazole, eliquis, sertraline, sotalol, lat-anprost	pantoprazole; apixaban; sertraline; sotalol	1
pl 08553/0246 sertraline film?coated tablets	sertraline hydrochloride	1

seronex [sertraline hydrochloride]	sertraline hcl	1
sertraline tablets, usp, 50 mg	sertraline hydrochloride	1
ciprofloxacin, sertraline, promacta, valacyclovir	ciprofloxacin; sertraline; eltrombopag; valaciclo-vir	1
sertraline; trazodone	sertraline; trazodone	1
sertraline 50mg, tylenol 325mg	sertraline; paracetamol	1
sertraline hcl tab 50mg	sertraline hydrochloride	1
sastium	sertraline hydrochloride	1
sertraline arrow 50 mg, gelule	sertraline hydrochloride	1
sertraline sandoz 50 mg gelule	sertraline hydrochloride	1
sertraline 500mg	sertraline	1
sertraline acyclovir	acyclovir; sertraline	1
teva?sertraline	sertraline hydrochloride	1
ic sertraline hcl 100 mg tablet	sertraline hydrochloride	1
zoloft; junel fe; b12; omeprazole	sertraline; junel fe; cyanocobalamin (vitamin b12); omeprazole	1
sertraline hhydrochloride	sertraline	1
generic zoloft 100mg	sertraline hydrochloride	1
zoloft 50 mg tab	sertraline	1
sertraline hydrochloride 100mg	sertraline hydrochloride	1
gabapentin, lorazepam, stimulant, levetiracetam, sertraline	gabapentin; lorazepam; stimulant; levetiracetam; sertraline	1
sertralin?mepha	sertraline hydrochloride	1
potassium, lyrica, calcium gluconate, vicodin, sertraline, probiotic	potassium; pregabalin; calcium gluconate; para-cetamol; sertraline	1
sertraline quetiapine	quetiapine; sertraline	1
sertraline 100mg qd	sertraline	1
sertraline 50 mg tab	sertraline	1
sertraline 50 mg tab cipl	sertraline hydrochloride	1
sertraline 200mg qd	sertraline	1
zoloft (sertaline)	sertraline	1
sertraline film?coated tablets 50mg	sertraline hydrochloride	1
sertraline hcl tabs	sertraline hydrochloride	1
sertraline vit d 3	penicillamine; sertraline	1
sertraline hcl 100	sertraline hydrochloride	1
sertraline hcl 50	sertraline hydrochloride	1
sertraline arrow lab 25 mg capsules	sertraline hydrochloride	1
sertraline (teva)	sertraline	1
sertraline hydrochloride 100 mg	sertraline hydrochloride	1
sertraline hcl 50mg tablets	sertraline hydrochloride	1

sertraline accord healthcare	sertraline hydrochloride	1
zoloft, copaxone	sertraline; glatiramer (copolymer 1)	1
sertralin 100 mg film coated tablets	sertraline hydrochloride	1
sertraline film coated tablets 50mg	sertraline hydrochloride	1
sertraline hydrocholoride	sertraline hydrochloride	1
sertraline hydrochloride / brand name not specifiedsertraline hydro.	sertraline hcl	1
unspecified authorized generic zoloft	sertraline	1
unspecified generic zoloft	sertraline	1
zoloft 100 mg po	sertraline	1
sertralin(sertraline)	sertraline	1
sertraline succinate	succinate; sertraline	1
sertraline(for trigeminal neuropathy)	sertraline	1
sertraline 100mg po nightly	sertraline	1
sertraline 75mg po daily	sertraline	1
zoloft 150mg	sertraline	1
zoloft 50mg 1 tab po qday	sertraline	1
zoloft 100mg 1 tab po qday	sertraline	1
zoloft 150 mg po	sertraline	1
sertraline sanis	sertraline	1
sertraline (zoloft) 100 mg tablet	sertraline	1
sertraline oral solution	sertraline	1
ic sertraline hcl 50 mg	sertraline	1
adult sertraline	sertraline	1
sertraline 25 mg tablets	sertraline	1
zoloft 50x1	sertraline	1
sertraline 100 mg tab	sertraline	1
sertraline con 20mg/ml	sertraline	1
sertraline (different brand)	sertraline	1
sertraline pow	sertraline	1
sertraline 75mg every morning	sertraline	1
sertraline 50 mg - tablet	sertraline	1
sertraline krka	sertraline	1
zoloft (consumer unsure if this is the name)	sertraline	1
sertraline 12.5 mg po qd	sertraline	1
sertraline 100mg/ atomoxetine 80mg	atomoxetine; sertraline	1
zoloft 25 mg taken from ovulation to end of menstrual cycle	sertraline	1
sertraline 200 mg/day	sertraline	1

zoloft calcium	calcium; sertraline	1
zoloft 100 mg daily	sertraline	1
sertraline 100mg oral tablet	sertraline	1
50 mg sertraline, tablets	sertraline	1
sertraline hcl 150 mg capsule	sertraline	1
sertraline h cap 200mg	sertraline	1
zoloft,	sertraline	1
sertraline hydrochloride gabapentin	gabapentin; sertraline hcl	1
sertraline hcl 100mg	sertraline	1
sertraline mcl	sertraline	1
sertraline 100 mg po qd	sertraline	1
sertraline h tab 100 mg	sertraline	1
sertraline h tab 100mg	sertraline	1
zoloft 100 milligram	sertraline	1
sertraline (zoloft) tablet	sertraline	1
1 6-28-21 sertraline 25 mg 1xday	sertraline	1
sertraline beyaz	beyaz; sertraline	1
sertraline h tab 25mg	sertraline	1
sertraline albuterol inhaler	salbutamol; sertraline	1
lustral 50mg	sertraline	1
zoloft after discontinuing vyvanse	lisdexamfetamine; sertraline	1
mirena iud zoloft	sertraline; levonorgestrel	1
sertralin krka [sertraline]	sertraline	1
sertraline (zoloft) 50 mg	sertraline	1
sertraline 50 mg qd	sertraline	1
sertraline: sertraline		1
sertraline 50 mg (1xday)	sertraline	1
gladem / sertraline hydrochloride	sertraline hcl	1
sertraline tab50mg	sertraline	1

Table S3. The dictionary used for filtering Escitalopram was applied.

**drugname(136445)**	**prod_ai**	**AE**
lexapro	escitalopram oxalate	70681
escitalopram	escitalopram oxalate	37598
cipralex	escitalopram oxalate	6891
escitalopram oxalate.	escitalopram oxalate	3972
escitalopram oxalate	escitalopram oxalate	3834
seroplex	escitalopram oxalate	3761

escitalopram oxalate - bp	escitalopram oxalate	2070
seropram	citalopram hydrobromide; escitalopram oxalate	1176
cipralex (escitalopram)	cipralex (escitalopram)	720
sipralexa	escitalopram oxalate	357
lexapro (escitalopram oxalate)	escitalopram	317
entact	escitalopram oxalate	289
seroplex (escitalopram)	seroplex (escitalopram)	283
escitalopram oxalate unk	escitalopram oxalate	281
escitalopram (escitalopram)	escitalopram	258
exodus	escitalopram oxalate; nicotine	186
escitalopram oxalate ? bp	escitalopram oxalate	185
lexapro /usa/ (escitalopram)	escitalopram	92
reconter	escitalopram oxalate	90
elicea	escitalopram oxalate	81
apo-escitalopram	escitalopram oxalate	77
lexapro (escitalopram oxalate) (escitalopram oxalate)	lexapro (escitalopram oxalate) (escitalopram oxalate)	69
escitalopram tablets usp 10mg	escitalopram oxalate	62
escitalopram accord	escitalopram oxalate	59
seroplex film-coated tablets	escitalopram oxalate	50
escitalopram arrow	escitalopram oxalate	46
espran	escitalopram oxalate	46
escitalopram 20 mg	escitalopram oxalate	45
generic lexapro	escitalopram oxalate	43
lexapril	escitalopram oxalate	43
escitalopram film-coated tablets	escitalopram oxalate	41
seroplex (escitalopram oxalate)	escitalopram oxalate	40
escitalopram oxalate (escitalopram oxalate)	escitalopram oxalate	38
escitalopram stada	escitalopram oxalate	37
escitalopram tablets usp 20mg	escitalopram oxalate	35
escitalopram 10mg	escitalopram oxalate	35
escitalopram hexal	escitalopram oxalate	34
escitalopram teva	escitalopram oxalate	34
zytomil	escitalopram oxalate	34
lexapro (all other therapeutic products)	escitalopram	33
escitalopram oral solution	escitalopram	32
exodus/escitalopram oxalate	escitalopram oxalate	32
escitalopram oxalate (lexapro)	escitalopram oxalate (lexapro)	31
cipralex (escitalopram oxalate)	cipralex (escitalopram oxalate)	31

lexapro (con.)	escitalopram	30
lexapro (escitalopram oxalate) (unknown)	lexapro (escitalopram oxalate) (unknown)	27
sipralexa (escitalopram)	escitalopram	25
escitalopram (escitalopram oxalate) (tablets)	escitalopram	24
teva escitalopram	escitalopram oxalate	23
lexapro (escitalopram)	lexapro (escitalopram)	22
citoles	escitalopram oxalate	21
escitalopram [escitalopram oxalate]	escitalopram oxalate	20
escitalopram 10mg tablet	escitalopram oxalate	20
lexapro /usa/(escitalopram)	escitalopram	20
escitalopram oxalate tablets usp, 10 mg	escitalopram oxalate	19
escitalopram (unknown)	escitalopram	18
escitalopram 10 mg	escitalopram	18
escitalopram 20mg	escitalopram	17
lexapro (escitalopram oxalate) (tablets)	escitalopram	16
escitalopram/escitalopram oxalate	escitalopram/escitalopram oxalate	16
esc	escitalopram oxalate	16
loxalate	escitalopram oxalate	15
escitalopram tablets usp 5mg	escitalopram oxalate	14
teva-escitalopram	escitalopram oxalate	14
escitalopram (unblinded) (escitalopram)	escitalopram	13
escitalopram oxalate tablets usp, 20 mg	escitalopram oxalate	13
lexapro (ssri)	escitalopram	13
exodus (escitalopram oxalate)	escitalopram oxalate	12
escitalopram lundbeck	escitalopram oxalate	12
escitalopram (lexapro)	escitalopram (lexapro)	12
heipram	escitalopram oxalate	12
cipralex [escitalopram]	cipralex [escitalopram]	11
escitalopram apotex	escitalopram oxalate	11
seropam	escitalopram oxalate	11
escitalopram oxalate tablets usp, 5 mg	escitalopram oxalate	10
seroplex 10 mg, comprim? pellicul? s?cable	escitalopram oxalate	10
seroplex 5 mg, comprime pellicule	escitalopram oxalate	10
lexapro 20mg - generic	escitalopram oxalate	10
escitalopram alter	escitalopram alter	10
zytomil tablets	escitalopram oxalate	10
lexapro generic	escitalopram oxalate	10
seroplex tablets	escitalopram oxalate	10
ipran	escitalopram oxalate	10

lexapro (escitalopram oxalate) (20 milligram)	escitalopram	9
escitalopram (escitalopram oxalate)	escitalopram (escitalopram oxalate)	9
cipralex(escitalopram)	escitalopram	9
cipralex/escitalopram	escitalopram	9
ecitalopram	escitalopram oxalate	9
citraz	escitalopram oxalate	9
citram	escitalopram oxalate	9
stalopam	escitalopram oxalate	9
escitalopram krka	escitalopram	9
lexapro(escitalopram oxalate)(escitalopram oxa-late)	escitalopram oxalate; escitalopram	8
escitalopram (open label) (escitalopram)	escitalopram	8
seroplex 10 mg, comprime pellicule secable	escitalopram oxalate	8
escitalopram (double-blind) (escitalopram)	escitalopram	8
lexapro(escitalopram oxalate)	lexapro(escitalopram oxalate)	8
lexapro film-coated tablets	escitalopram	8
lexapro/escitalopram	lexapro/escitalopram	8
entact (escitalopram)	escitalopram	8
ran escitalopram	ran escitalopram	8
serpentil	escitalopram oxalate	8
escitalopram oxalate tablets	escitalopram oxalate	8
jamp escitalopram	escitalopram	8
seroplex (escitalopram oxalate) (escitalopram oxalate)	escitalopram oxalate;	7
seroplex (escitalopram) (escitalopram)	escitalopram;	7
seroplex 20 mg, comprim? pellicul? s?cable	escitalopram oxalate	7
lexapro (escitalopram oxalate0	escitalopram	7
escitalopram film?coated tablets	escitalopram oxalate	7
seroplex 5 mg, comprim? pellicul?	escitalopram oxalate	7
escitalopram oral drops	escitalopram oral drops	7
escitalopram (citalopram)	escitalopram (citalopram)	7
lexapro/usa/(escitalopram)	escitalopram	7
escitalopram glenmark	escitalopram oxalate	7
cipralex meltz	escitalopram oxalate	7
sipralexa tablet	escitalopram oxalate	7
escitalopram biogaran	escitalopram oxalate	7
escitalopram (escitalopram oxalate) (10 milli-gram, tablets)	escitalopram	6
cipralex meltz [escitalopram oxalate]	cipralex meltz [escitalopram oxalate]	6
seroplex 15 mg, comprime pellicule secable	escitalopram oxalate	6

cipralex/escitalopram oxalate	cipralex/escitalopram oxalate	6
exodus [escitalopram oxalate]	exodus [escitalopram oxalate]	6
escitalopram oxalate tablet	escitalopram oxalate	6
serpentil [escitalopram oxalate]	escitalopram oxalate	6
esertia (escitalopram)	escitalopram	6
escitalopram (oxalate d)	escitalopram oxalate	6
escitalopram 20mg tablet	escitalopram oxalate	6
escitalopram normon	escitalopram normon	6
excitalopram oxalate	escitalopram oxalate	6
escitalopram genedec	escitalopram oxalate	6
escitalopram-tablets	escitalopram-tablets	6
escitalopramum	escitalopramum	6
apo escitalopram	escitalopram oxalate	6
seropram nos	citalopram hydrobromide or citalopram hydrochloride or escitalopram oxalate	6
esoprex [escitalopram oxalate]	escitalopram oxalate	6
lexapro (escitalopram oxalate) (10 milligram)	escitalopram	5
escitalopram oxalate- escitalopram oxalate tablet	escitalopram oxalate	5
lexapro (escitalopram oxalate)(escitalopram oxalate)	escitalopram oxalate; escitalopram	5
lexapro film-coated tablets (escitalopram-tablets) (escitalopram)	escitalopram	5
escitalopram oxalate 5mg tablet prescription f 53 = [aurobindo pharma limited	escitalopram oxalate	5
escitalopram 10mg aurobiudo pharma	escitalopram	5
escitalopram (escitalopram) unknown	escitalopram	5
escitalopram 5 mg film-coated tablets	escitalopram oxalate	5
blinded escitalopram oxalate	escitalopram oxalate	5
lexapro (escitalopram oxalate)	lexapro (escitalopram oxalate)	5
escitalopram oxalate w/etizolam	escitalopram\etizolam	5
lexpro (interpreted as lexapro)	escitalopram	5
escitalopram oxalate escitalopram	escitalopram oxalate	5
escitalopram orion	escitalopram oxalate	5
escitalopram mepha	escitalopram oxalate	5
escitalopram almus	escitalopram oxalate	5
accord escitalopram	escitalopram oxalate	5
escitalopram (con.)	escitalopram	5
lexapro generic 10mg	escitalopram oxalate	5
escitalopram tecnigen	escitalopram oxalate	5
escitalopram an	escitalopram oxalate	5

seroplex 10mg	escitalopram oxalate	5
escitaopram	escitalopram oxalate	5
lexam	escitalopram oxalate	5
escitalopram.		5
escitalopram (escitalopram oxalate) (escital-opram oxalate)	escitalopram oxalate; escitalopram	4
lexapro (escitalopram oxalate) (tablet) (escital-opram oxalate)	escitalopram oxalate; escitalopram	4
scitalax 10 mg com rev ct bl al plas inc x 28	escitalopram oxalate	4
escitalopram abz 5 mg filmtabletten	escitalopram oxalate	4
lexapro (escitalopram oxalate) tablet	escitalopram	4
lexapro(escitalopram oxalate)(unknown)	lexapro(escitalopram oxalate)(unknown)	4
lexapro(escitalopram oxalate) (unknown)	lexapro(escitalopram oxalate) (unknown)	4
escitalopram arrow film-coated tablet 5mg	escitalopram oxalate	4
escitalopram oxalate 10mg accord hlthcare	escitalopram oxalate	4
escitalopram (escitalopram) (escitalopram)	escitalopram	4
escitalopram arrow film-coated tablet 10mg	escitalopram oxalate	4
seroplex 20 mg, comprime pellicule secable	escitalopram oxalate	4
escitalopram 10 mg film-coated tablets efg	escitalopram oxalate	4
cipralex /den/ (escitalopram)	escitalopram	4
lexapro (escitalopram) tablets	escitalopram	4
cipralex [escitalopram oxalate]	cipralex [escitalopram oxalate]	4
lexapril (escitalopram oxalate)	escitalopram oxalate	4
escitalopram oxalate (citalopram)	escitalopram oxalate	4
lexapro 10mg (generic)	escitalopram	4
escitalopram (seroplex)	escitalopram	4
entact - h. lundbeck a/s	escitalopram oxalate	4
escitalopram oxalate teva	escitalopram oxalate	4
lexapro (antidepressants)	escitalopram	4
escitalopram(escitalopram)	escitalopram(escitalopram)	4
seroplex(escitalopram)	seroplex(escitalopram)	4
escitalopram (double-blind)	escitalopram	4
esto (escitalopram oxalate)	escitalopram oxalate	4
escitalopram cinfa	escitalopram oxalate	4
escitalopram, 10 mg	escitalopram oxalate	4
escitalopram (ssri)	escitalopram	4
escitalopram oxylate	escitalopram oxalate	4
escitalopram tab 20mg	escitalopram tab 20mg	4
escitalopram tab	escitalopram tab	4

ran- escitalopram	ran- escitalopram	4
zytomil 20 mg	escitalopram oxalate	4
lexatin [escitalopram oxalate]	escitalopram oxalate;	4
lexapro 10 mg tablet	escitalopram	4
escitalopram actavis tab 10 mg	escitalopram oxalate	4
oxalate of escitalopram	escitalopram; oxalate	4
escitalopram oxalate\flupentixol dihydrochloride	escitalopram oxalate\flupentixol dihydrochloride	4
escitalopram mylan generiques 5 mg, comprim? pellicul?	escitalopram oxalate	3
lexapro (escitalopram oxalate) (10 milligram, tablets)	escitalopram	3
lexapro (escitalopram oxalate) (20 milligram, tablets)	escitalopram	3
seroplex (seroplex - escitalopram oxalate) (not specified)	escitalopram oxalate;	3
lexapro (escitalopram oxalate) (10 milligram, tablets) (escitalopram o	escitalopram	3
escitalopram (generic for lexapro)	escitalopram oxalate	3
escitalopram (escitalopram) (unknown)	escitalopram (escitalopram) (unknown)	3
lexapro (escitalopram oxalate) tablets	escitalopram	3
cipralex (escitalopram) (escitalopram)	escitalopram	3
escitalopram arrow film-coated tablets	escitalopram oxalate	3
escitalopram basics 10 mg filmtabletten	escitalopram oxalate	3
escitalopram (open-label) (escitalopram)	escitalopram	3
escitalopram oxalate (escitalopram) tablet	escitalopram oxalate	3
seroplex 15 mg, comprim? pellicul? s?cable	escitalopram oxalate	3
escitalopram (escitalopram) tablet	escitalopram	3
lexapro (escitalopram oxalate) tablet, 10 mg	escitalopram	3
lexapro (esctalopram oxalate)	oxalate; escitalopram	3
lexpro (escitalopram oxalate)	escitalopram oxalate	3
entact (escitalopram oxalate)	entact (escitalopram oxalate)	3
lexapro (escitalopram oxylate)	lexapro (escitalopram oxylate)	3
escitalopram tablets usp 20 mg	escitalopram	3
lexapro (esictalopram oxalate)	oxalate; escitalopram	3
lexapro 9escitalopram oxalate)	oxalate; escitalopram	3
serpentil (escitalopram oxalate)	escitalopram oxalate	3
escitalopram oxalate 10mg tablet	escitalopram oxalate	3
escitalopram tab 20 mg	escitalopram oxalate	3
sipralexa(escitalopram)	escitalopram	3
escitalopram tablet 10mg	escitalopram tablet 10mg	3

escitalopram 10 mg daily	escitalopram 10 mg daily	3
escitalopram 20 mg mylan	escitalopram	3
escitalopram tablet 20mg	escitalopram oxalate	3
auro-escitalopram tablets	escitalopram oxalate	3
escitalopram oxalate 10 mg	escitalopram oxalate	3
escitalopram tab 10 mg	escitalopram tab 10 mg	3
cipralex[escitalopram]	cipralex[escitalopram]	3
escitalopram basics	escitalopram oxalate	3
sandoz escitalopram	sandoz escitalopram	3
lexapro(escitalopram)	lexapro(escitalopram)	3
exodus (escitalopram)	pyrantel	3
mylan escitalopram 10	escitalopram oxalate	3
escitalopram (tablets)	escitalopram (tablets)	3
(escitalopram)	escitalopram	3
ran-escitalopram	ran-escitalopram	3
entact oral drops	escitalopram oxalate	3
entact 10 mg	escitalopram oxalate	3
escitaloprame	escitaloprame	3
esopram	escitalopram oxalate	3
lexapro tablets (escitalopram oxalate)	escitalopram oxalate; escitalopram	3
escitalopram oxlate	escitalopram	3
escitalopram torrent	escitalopram	3
escitalopram 20 mg tablet	escitalopram	3
escitalopram bluefish	bluefish; escitalopram	3
escitalopram tab 5mg	escitalopram	3
seroplex (escitalopram oxalate) (escitalopram)	escitalopram;	2
escitalopram 10 mg teva, filmomhulde tabletten	escitalopram oxalate	2
escitalopram 20 mg (escitalopram) unknown, 20mg	escitalopram oxalate	2
escitalopram teva ritecol may have -301-267-2955	escitalopram	2
lexapro (all other therapeutic products) tablets	escitalopram	2
escitalopram oxalate (escitalopram oxalate) unknown	escitalopram oxalate	2
lexapro (escitalopram oxlate) (escitalopram oxalate)	escitalopram oxalate; escitalopram	2
escitalopram oxalate (all other therapeutic products)	escitalopram oxalate	2
cipralex (escitalopram oxalate) (escitalopram oxalate)	escitalopram oxalate	2
escitalopram generics 10mg film-coated tablet	escitalopram oxalate	2

escitalopram mylan g?n?riques 5 mg, comprim? pellicul? s?cable	escitalopram oxalate	2
seroplex (escitalopram oxalate) (tablets) (escital-opram oxalate)	escitalopram oxalate;	2
escitalopram (escitalopram oxalate) (tablets) (escitalopram oxalate)	escitalopram oxalate; escitalopram	2
lexapro (escitalopram oxalate) (20 milligram, tablets) (escitalopram o	escitalopram	2
lexapro (escitalopram oxalate) (escitalopram oxalate) (escitalopram oxalate)	escitalopram oxalate; escitalopram	2
escitalopram (generic lexapro) 20mg	escitalopram oxalate	2
escitalopram oxalate 10mg tab (tbd)	escitalopram oxalate	2
escitalopram oxalate 20mg tab (tbd)	escitalopram oxalate	2
escitalopram (lexapro) (escitalopram)	escitalopram (lexapro) (escitalopram)	2
lexapro (escitalopram oxalate), 600 mg	lexapro (escitalopram oxalate), 600 mg	2
escitalopram oxalate - unknown - 10 mg	escitalopram	2
escitalopram (unblinded)(escitalopram)	escitalopram	2
entact oral drops (escitalopram drops)	escitalopram	2
escitalopram oxalate tablets 10 mg	escitalopram oxalate	2
escitalopram arrow film-coated tablet 20mg	escitalopram oxalate	2
escitalopram (double blind) (escitalopram)	escitalopram	2
lexapro (escitalopram oxalate) tablet, 10mg	escitalopram	2
escitalopram arrow 5 mg, comprim? pellicul?	escitalopram oxalate	2
lexapro (escitalopram oxalate) tablet, 20mg	escitalopram	2
seroplex film-coated tablets (escitalopram)	escitalopram;	2
escitalopram (escitalopram oxalate 20mg tab)	escitalopram oxalate	2
escitalopram (escitalopram oxalate 10mg tab)	escitalopram oxalate	2
escitalopram teva 10 mg filmdragerad tablett	escitalopram oxalate	2
escitalopram teva 20 mg filmdragerad tablett	escitalopram oxalate	2
escitalopram/lexapro generic	escitalopram	2
seroplex 5 mg, coated tablet	escitalopram oxalate	2
escitalopram oxalate 5mg tab	escitalopram oxalate	2
cipralex (escitalopram, , 0)	escitalopram	2
lexapro escitalopram oxalate)	escitalopram oxalate; escitalopram	2
lexapro (scitalopram oxalate)	oxalate; escitalopram	2
escitalopram 20 mg comprimido	escitalopram 20 mg comprimido	2
escitalopram oxalato (2858ob)	escitalopram oxalate	2
lexapro (escitalpram oxalate)	oxalate; escitalopram	2
lexaro (escitalopram oxalate)	escitalopram oxalate	2
lexapro (escitaloprm oxalate)	oxalate; escitalopram	2

seroplex(escitalopram oxalate)	seroplex(escitalopram oxalate)	2
zavesca [escitalopram oxalate]	zavesca [escitalopram oxalate]	2
escitalopram oxalate (unknown)	escitalopram oxalate	2
aciprex [escitalopram oxalate]	aciprex [escitalopram oxalate]	2
lexapro (escitalopral oxalate)	oxalate; escitalopram	2
levapro (escitalopram oxalate)	escitalopram oxalate	2
lexapro (excitalopram oxalate)	oxalate; escitalopram	2
lexapor (escitalopram oxalate)	escitalopram oxalate	2
lexapro (escitalopram ocalate)	escitalopram	2
seropram [escitalopram oxalate]	seropram [escitalopram oxalate]	2
seropram (escitalopram oxalate)	escitalopram oxalate	2
escitalopram oxalate (seroplex)	escitalopram oxalate (seroplex)	2
lexapro (escitralopram oxalate)	oxalate; escitalopram	2
sipralexa (escitalopram oxalate)	sipralexa (escitalopram oxalate)	2
escitalopram oxalate 20mg, 20 mg	escitalopram oxalate	2
lexapro (escitalopram) (tablets)	escitalopram	2
espran (inn:escitalopram oxalate)	escitalopram oxalate	2
escitalopram 20 mg 28 comprimidos	escitalopram 20 mg 28 comprimidos	2
cipralex (escitalopram) (tablets)	escitalopram	2
reconter (escitalopram)	reconter (escitalopram)	2
escitalopram 5mg amneal	escitalopram oxalate	2
lexapril (escitalopram)	escitalopram	2
escitalopram 10mg daily	escitalopram 10mg daily	2
escitalopram 20mg daily	escitalopram oxalate	2
escitalopram (cipralex)	escitalopram	2
escitalopram ratiopharm	escitalopram oxalate	2
lexapro (not specified)	escitalopram	2
escitalopram 10mg camber	escitalopram oxalate	2
escitalopram 20 mg daily	escitalopram oxalate	2
escitalopram 20mg unknown	escitalopram oxalate	2
escitalopram-form-unknown	escitalopram-form-unknown	2
escitalopram 20 mg camber	escitalopram oxalate	2
escitalopram 20mg generic	escitalopram	2
escitalopram spirig hc	escitalopram spirig hc	2
cipralex (escitalopram, 0)	escitalopram	2
lexparo (escitalopram)	escitalopram	2
escitalopram 9escitalopram)	escitalopram	2
escitalopram (escitalopram0	escitalopram	2
lexapro/usa/ (escitalopram)	escitalopram	2

escitalopram, 10mg	escitalopram oxalate	2
escitalopram clamed	escitalopram clamed	2
escitalopram arcana	escitalopram arcana	2
escitalopram sandoz	escitalopram sandoz	2
escitalopram scippa	escitalopram scippa	2
citoles (drop form)	escitalopram oxalate	2
escitalopram amneal	escitalopram oxalate	2
escitalopram zentiva	escitalopram oxalate	2
escitalopram +pharma	escitalopram +pharma	2
generic escitalopram	escitalopram oxalate	2
external-escitalopram	escitalopram oxalate	2
escitalopram 20 mg/ml	escitalopram oxalate	2
escitalopram generics	escitalopram generics	2
lexapro (escitaloprim)	escitalopram	2
escitalopram 10mg teva	escitalopram oxalate	2
cipralex 20 mg	escitalopram oxalate	2
seroplex 10 mg	escitalopram oxalate	2
lexapro (prev.)	escitalopram	2
act escitalopram	escitalopram oxalate	2
lexapro 10 mg	escitalopram	2
escitalopram 15mg	escitalopram 15mg	2
escitalopram drla	escitalopram drla	2
lexapro (lexapro)	escitalopram	2
escitolapram	escitalopram oxalate	2
cipra pro	escitalopram oxalate	2
espram	escitalopram oxalate	2
prolide	escitalopram oxalate	2
escitalopram?mepha	escitalopram oxalate	2
escitalopram 10 mg po daily	escitalopram	2
b lexapro	escitalopram	2
escitalopram mylan 10 mg filmtabletten	escitalopram oxalate	2
escitalopram arrow film?coated tablet 10mg	escitalopram oxalate	2
escitalopram,	escitalopram	2
co escitalopram	escitalopram	2
escitalopram arrow film?coated tablets	escitalopram oxalate	2
escitalopram mylan generics	escitalopram oxalate	2
auro?escitalopram tablets	escitalopram oxalate	2
lextor	escitalopram oxalate	2
ex3	escitalopram oxalate	2

stalopam film c.tabs 10 mg	escitalopram oxalate	2
espran 10	escitalopram oxalate	2
seroplex 15 mg	escitalopram oxalate	2
teva?escitalopram	escitalopram oxalate	2
clonazepam + escitalopram	escitalopram; clonazepam	2
lexapro 10mg daily	escitalopram	2
auro-escitalopram	escitalopram; carbamide peroxide	2
cital (escitalopram oxalate)	escitalopram oxalate	2
lexapro 20mg (once per day	escitalopram	2
folcres [escitalopram]	escitalopram	2
escitalopram (g)	escitalopram	2
escitalopram cinfa 20mg gd film-coated tablets, 56tablets	escitalopram	2
escitalopram (escitaloppram oxalate) (tablets)	oxalate; escitalopram	1
cipralex (escitalopram oxalate) coated tabltes	escitalopram oxalate	1
lexapro (estcitalopram oxalate) (20 milligram)	oxalate; escitalopram	1
ferosul, pantoprazole, escitalopram, budesonide	ferosul, pantoprazole, escitalopram, budesonide	1
esertia (escitalopram) (tablets) (escitalopram)	escitalopram	1
escitalopram 10 mg tablet - oval aurobindo pharm	escitalopram oxalate	1
escitalopram (lexapro) 10 mg po tablet [5215077]	escitalopram	1
escitalopram 20 mg intas pharmaceuticals limited	escitalopram oxalate	1
lexapro (escitalopram oxalate) 11/22/2008 to unk	lexapro (escitalopram oxalate) 11/22/2008 to unk	1
nitroglycerin, xifaxan, climara pro, escitalopram	nitroglycerin, xifaxan, climara pro, escitalopram	1
escitalopram aurobindo filmomhulde tabletten 10mg	escitalopram oxalate	1
lexapro generic 20mg generic---i do not know more	escitalopram oxalate	1
escitalopram oral solution (escitalopram oxalate)	escitalopram	1
escitalopram tablet 10mg tablet, 10 mg (milli-gram)	escitalopram oxalate	1
lexapro (escitalopram oxalate) formulation un-known	escitalopram	1
escitalopram (escitalopram oxalate) (5 mg, tab-lets)	escitalopram	1
etalopro 5mg escitalopram oxalate clonmel healthcare	escitalopram oxalate	1
escitalopram arrow 10 mg, comprim? pellicul? s?cable	escitalopram oxalate	1
escitalopram arrow 20 mg, comprim? pellicul? s?cable	escitalopram oxalate	1
seroplex(escitalopram oxalate)(escitalopram	seroplex(escitalopram oxalate)(escitalopram	1

oxalate)	oxalate)	
lexapro 5 mg. escitalopram oxalate q23624 tablet tev	escitalopram; escitalopram oxalate	1
lexapro (escitalopram oxalate (escitalopram oxalate)	escitalopram oxalate; escitalopram	1
lexopro (escitalopram oxalate) (escitaloprm oxalate)	oxalate; escitalopram oxalate	1
escitalopram (escitalopram) (unknown) (escital-opram)	escitalopram	1
exapro (escitalopram oxalate) (escitalopram oxalate)	escitalopram oxalate	1
heipram 10 mg tablets film?coated tablets, 56 tablets	escitalopram oxalate	1
escitalopram 5 mg substitute for lexapro 5. mg tablet	escitalopram oxalate	1
escitalopram oxalate (escitalopram oxalate) (un-known)	escitalopram oxalate (escitalopram oxalate) (un-known)	1
lexapro (escitalopram calcium) (escitalopram calcium)	calcium; escitalopram	1
cipralex (escitalopram oxalate)(escitalopram oxalate)	escitalopram oxalate	1
cipralex (escitalopram oxalate (escitalopram oxalate)	escitalopram oxalate	1
bupropion vs escitalopram (bupropion hydro-chloride xl)	bupropion hydrochloride	1
lexapro (escitalopram oxalate) (escitalopram oxalate)?	escitalopram oxalate; escitalopram	1
lexapro (escitalopram oxalate) 20 milligram, tableets)	lexapro (escitalopram oxalate) 20 milligram, tableets)	1
seroplex (escitalopram oxalate) (esoitalopram oxalate)	seroplex (escitalopram oxalate) (esoitalopram oxalate)	1
lexapro (escitalopram oxalate) (tablet) (escital-opram)	escitalopram	1
entact - compresse rivestite con film - h. lundbeck a/s	escitalopram oxalate	1
lexapro (escitalopram oxalate) (unknown) (es-citalopram)	escitalopram	1
lexapro (escitalopram oxalate) (10 milligram, unknown)?	escitalopram	1
cipralex (escitalopram oxalate) (being queried) (10 mg)	escitalopram	1
lexapro film-coated tablets (escitalopram) (es-citalopram)	escitalopram	1
escitalopram oxalate (escitalopram oxalate) (10 milligram)	escitalopram	1
escitalopram(escitalopram oxalate) (10 milli-gram, tablets)	escitalopram	1

escitalopram (escitalopram oxalate) (20 milli-gram, talbets)	escitalopram	1
escitalopram (escitalopram oxalate) (20 milli-gram, tablets)	escitalopram	1
escitalopram oxalate oral solution 5 mg5ml batch no. e162342	escitalopram oxalate	1
escitalopram oral solution (escitalopram oxalate) (solution)	escitalopram oxalate; escitalopram	1
escitalopram 20 mg tab - shape round, color white, side 1 p20	escitalopram oxalate	1
entact - 10 mg compresse rivestite con film - h. lundbeck a/s	escitalopram oxalate	1
lexapro (escitalopram oxa-late)(tablets)(escitalopram oxalate)	escitalopram oxalate; escitalopram	1
seroplex (escitalopram oxa-late)(tablets)(escitalopram oxalate)	escitalopram oxalate;	1
lexapro (escitalopram oxalate) (10 milligram, tablets) tablets	escitalopram	1
nitroglycerin, lexapro, escitalopram, atorvastatin, fluticasone	nitroglycerin, lexapro, escitalopram, atorvastatin, fluticasone	1
lexapro (escitalopram oxalate) (tablets) (escital-opram oxalate)	escitalopram oxalate; escitalopram	1
lexapro (escitalopram oxalate) (unknwon) (es-citalopram oxalate)	escitalopram oxalate; escitalopram	1
lexapro (escitalopram oxalate) (unknown) (es-citalopram oxalate)	escitalopram oxalate; escitalopram	1
escitalopram oxalate oral soln, 5 mg camber pharmaceuticals, inc	escitalopram oxalate	1
hydrochlorothiazide, advair, atorvastatin, zolpidem, escitalopram	hydrochlorothiazide, advair, atorvastatin, zolpidem, escitalopram	1
lexapro(escitalopram oxalate)(escitalopram oxa-late) :1) - ongoing	lexapro(escitalopram oxalate)(escitalopram oxa-late) :1) - ongoing	1
escitalopram(escitalopram) (10 milligram, un-known) (escitalopram)	escitalopram	1
seroplex (escitalopram oxalate)(10 mg, coated tablet)(escitalopram)	; escitalopram	1
escitalopram flas cinfa 10 mg comprimidos bu-codispersables efg , 28.	escitalopram oxalate	1
lexapro (escitalopram oxalate) (40 milligram) (escitalopram oxalate)	escitalopram oxalate; escitalopram	1
escitalopram (escitalopram oxalate) (tablets)-(escitalopram oxalate)	escitalopram oxalate; escitalopram	1
escitalopram (escitalopram oxalate) (5 milligram, tablets) (5 mg,once	escitalopram	1
escitalopram ratio 10 mg comprimidos re-cubiertos con pelicula efg , 28	escitalopram oxalate	1
animaxen 20 mg tabletas recubiertas - escital-	escitalopram oxalate	1

opram 20 mg tabletas recu		
heipram 15 mg comprimidos recubiertos con pelicula efg, 56 comprimidos	escitalopram oxalate	1
escitalopram sandoz 10 mg comprimidos bu-codispersables efg, 28 comprim	escitalopram sandoz 10 mg comprimidos bu-codispersables efg, 28 comprim	1
escitalopram flas cinfa 10 mg tablets bucodis-persables efg, 28 tablets	escitalopram oxalate	1
seroplex (escitalopram oxalate) (10 mg, coated tablet) (escitalopram o	; escitalopram	1
escitalopram (escitalopram oxalate) (tablets) - (escitalopram oxalate)	escitalopram oxalate; escitalopram	1
lexapro (escitalopram oxalate) (10 milligram, tablet) (escitalopram ox	escitalopram	1
escitalopram (generic for lexapro) 1st presciption 10mg. 7/15/15 doubled to 20 m	escitalopram oxalate	1
seroplex (escitalopram oxalate) (20 mg, film-coated tablet) (escitalopram oxalat	; escitalopram	1
escitalopram 20mg accord healthcare	escitalopram oxalate	1
escitalopram 20 mg, generic lexapro	escitalopram oxalate	1
escitalopram oxalate tablets, 20 mg	escitalopram	1
lexapro (escitalopram oxalate) 10mg	escitalopram	1
excitalopram (escitalopram oxalate)	escitalopram oxalate	1
escitalopram oxalate (escitalopram)	escitalopram oxalate	1
escitalopram, 10mg accord healthcare	escitalopram oxalate	1
escitalopram abz 10 mg filmtabletten	escitalopram oxalate	1
escitalopram oxalate (lexapro) 20 mg	escitalopram oxalate (lexapro) 20 mg	1
lexapro (escitalopram)(escitalopram)	lexapro (escitalopram)(escitalopram)	1
lexapro (escitalopram oxalate)tablet	escitalopram	1
escitalopram (escitaloopram) unknown	escitalopram	1
lexapro (escitalopram oxalate), 5 mg	escitalopram	1
lexapro - generic escitalopram 20 mg	escitalopram	1
escitalopram (escitablopram oxalate)	oxalate; escitalopram	1
entact (escitalopram) (escitalopram)	escitalopram	1
seropram (escitalopram hydrobromide)	escitalopram;	1
cipralex/gfr/ (escitalopram oxalate)	escitalopram oxalate	1
lexapro (escitalopram oxalate) 20 mg	escitalopram	1
lexapro (escitalopram oxalat) tablet	escitalopram	1
lexapro oral solution (escitalopram)	escitalopram	1
lexapro (escitalopram) tablets 10 mg	escitalopram	1
cipralex /gr/ (escitalopram oxalate)	escitalopram oxalate	1
escitalopram film-coated tablets 15mg	escitalopram oxalate	1
escitalopram film coated tablets 15mg	escitalopram oxalate	1

seroplex 10 mg, seared skinted tablet	escitalopram oxalate	1
esertia (escitalopram) (escitalopram)	escitalopram	1
cipralex (escitalopram oxalate) /gfr/	escitalopram oxalate	1
escitalopram (escitalopram) (tablets)	escitalopram	1
seroplex (escitalopram)(escitalopram)	escitalopram;	1
cipralex /gfr/ (escitalopram oxalate)	escitalopram oxalate	1
lexapro (all other therapeutic drugs)	escitalopram	1
escitalopram tablet, 15 mg (milligram)	escitalopram oxalate	1
escitalopram oxalate oral tablet 20 mg	escitalopram oxalate oral tablet 20 mg	1
furosemide, escitalopram, levothyroxin	furosemide, escitalopram, levothyroxin	1
lexapro (escitalopram oxalate) unknown	escitalopram	1
citalopram hydrobromide (escitalopram)	citalopram hydrobromide	1
lexapro seroquel (quetiapine fumarate)	quetiapine; escitalopram	1
escitalopram: oral tablet (10mg), 10 mg	escitalopram oxalate	1
spiralexa (escitalopram) (escitalopram)	escitalopram	1
lexapro(escitalopram oxalate) (tablets)	escitalopram	1
escitalopram generic health 20mg tablet	escitalopram oxalate	1
lexapro 10mg tablet aurobindo (generic)	escitalopram oxalate	1
escitalopram 10 mg richter gedeon nyrt.	escitalopram oxalate	1
lexapro (escitalpram oxalate) (unknown)	oxalate; escitalopram	1
lexapro (escitalopram oxalte) (tablets)	escitalopram	1
escitalopram (escitalpram hydrobromide)	escitalopram	1
escitalopram (sipralexa) (escitalopram)	escitalopram	1
lexapro (all othr therapeutic products)	escitalopram	1
escitalopram generichealth 10 mg tablets	escitalopram oxalate	1
escitalopram oxalate generic for lexapro	escitalopram oxalate	1
escitalopram 10 mg, 10 mg lupin pharmacy	escitalopram oxalate	1
escitalopram heumann 10 mg filmtabletten	escitalopram oxalate	1
escitalopram (escitalopram oxate) tablet	escitalopram	1
cipralex active substances: escitalopram	escitalopram	1
escitalopram (escitalopram) tablet, 10mg	escitalopram	1
lexapro (all other therapeutic vitamins)	therapeutic vitamins; escitalopram	1
lexapro (all other therapetuic products)	escitalopram	1
escitalopram arrow film?coated tablet 5mg	escitalopram oxalate	1
lexapro 20 mg tabs (escitalopram oxalate)	escitalopram	1
lexapro (escitalopram oxalate) (unknown)?	escitalopram	1
seroplex (escitalopram oxalate) (tablets)	seroplex (escitalopram oxalate) (tablets)	1
escitalopram(escitalopram) (escitalopram)	escitalopram	1
lexapro (all other therapeutic products)	escitalopram	1

escitalopram teva 5 mg, comprim? pellicul?	escitalopram oxalate	1
escitalopram 10mg, 1 tablet by mouth daily	escitalopram 10mg, 1 tablet by mouth daily	1
escitalopram (escitalopram0 (escitalopram)	escitalopram	1
lexapro (escitalopram oxalate) tablet 20mg	escitalopram	1
seroplex (escitalopram oxalate) : from not	seroplex (escitalopram oxalate) : from not	1
lexapro (escitalopram oxalate) unspecified	escitalopram	1
escitalopram (escitalopram oxalate) (10mg)	escitalopram	1
lexapro film-coated tablets (escitalopram)	escitalopram	1
escitalopram oxalate (escitaloram oxalate)	oxalate; escitalopram oxalate	1
escitalopram (esctalopram oxalate 20mg tab)	escitalopram oxalate	1
escitalopram krka 5 mg filmdragerad tablett	escitalopram krka 5 mg filmdragerad tablett	1
escitalopram(escitalopram oxalate) oxalate)	escitalopram(escitalopram oxalate) oxalate)	1
escitalopram (unknown until not continuing)	escitalopram	1
amoxicillin w/clavulanate potassium lexapro	escitalopram; amoxicillin w / clavulanate potassium	1
lexapro(escitalopram oxalate) (5 milligram)	escitalopram	1
escitolopram oxalate (escitalopram oxalate)	oxalate	1
escitalopram (escitalopram oxalate) (talets)	escitalopram	1
escitaopram (escitalopram oxalate) (tablets)	escitalopram oxalate	1
escitalopram (esctalopram oxalate) (tablets)	oxalate; escitalopram	1
escitalopram (esitalopram oxalate) (tablets)	oxalate; escitalopram	1
lexapro oral soultion (escitalopram oxalate)	escitalopram oxalate; escitalopram	1
lexapro (escitalopram oxalate) tablet, 20 mg	escitalopram	1
lexapro (escitalopram oxalate (20 milligram)	escitalopram	1
escitaloptam (escitalopram oxalate) (tablets)	escitalopram oxalate	1
escitalopram (escitalopram ocalate) (tablets)	escitalopram	1
escitalopram (escitalopram oxatate) (tablets)	escitalopram	1
lexapro (escitalopram oxalate) (2o milligram)	escitalopram	1
escttalopram (escitalopram oxalate) (tablets)	escitalopram oxalate	1
generic escitalopram oxalate	escitalopram oxalate	1
lexapro escitalopram oxalate	escitalopram oxalate; escitalopram	1
escitalopram 5mg/5ml generic	escitalopram	1
lexapro (citalopram oxalate)	oxalate; citalopram; escitalopram	1
escitaloipram (escitalopram)	escitalopram	1
cipralex /den/(escitalopram)	escitalopram	1
escitalopram (escitalopram,)	escitalopram	1
escitalopram (eswcitalopram)	escitalopram	1
escitalopram (escilatorpoam)	escitalopram	1
escritalopram (escitalopram)	escitalopram	1

escitalopram 20 mg once daily	escitalopram 20 mg once daily	1
escitalopram oxlate (lexapro)	escitalopram oxlate (lexapro)	1
cipralex meltz [escitalopram]	escitalopram oxalate	1
escitalopram oxalate tab 20mg	escitalopram oxalate tab 20mg	1
escitalopram oxalate 20mg tab	escitalopram oxalate	1
lexapro (escitalopram oxalate	escitalopram oxalate; escitalopram	1
external-escitalopram oxalate	escitalopram oxalate	1
lexapro (escitalopramoxalate)	escitalopram	1
lexapro (esitalopram oxalate)	oxalate; escitalopram	1
lexapro (escitablopram oxate)	escitalopram	1
lexapro (escitalopram oxlate)	lexapro (escitalopram oxlate)	1
lexapro (escitalopram) tablet	escitalopram	1
lexapro /usa/) (escitalopram)	escitalopram	1
lexapro (ecitalopram oxalate)	oxalate; escitalopram	1
escitalopram 15 efg comprimido	escitalopram oxalate	1
escitalopram oxalate (escilex)	escitalopram oxalate (escilex)	1
seroplex (escitaloprm oxalate)	escitalopram oxalate	1
escitalopram tablets, usp teva	escitalopram oxalate	1
escitalopram-mepha lactab 10mg	escitalopram oxalate	1
escitalopram 10 mg generic/cvs	escitalopram oxalate	1
escitalopram (generic lexipro)	escitalopram oxalate	1
glenmark generics escitalopram	glenmark generics escitalopram	1
optiser [escitalopram oxalate]	optiser [escitalopram oxalate]	1
escitalopram (generic lexapro)	escitalopram oxalate	1
escitalopram (lexapro generic)	escitalopram (lexapro generic)	1
lexapro / escitalopram oxalate	lexapro / escitalopram oxalate	1
lexapro (escitalo[ram oxalate)	oxalate; escitalopram	1
lexapro (escitalopram oxilate)	lexapro (escitalopram oxilate)	1
lexapro tablet filmomhuld 20mg	escitalopram	1
escitalopram (lexapro) 10mg qd	escitalopram (lexapro) 10mg qd	1
lexapro 10 forest laboratories	escitalopram	1
escitalopram tab 10 mg torrent	escitalopram oxalate	1
lexipro (escitalopram oxalate)	escitalopram oxalate	1
lexapro (escitalopram hormone)	escitalopram	1
lexapro(10 milligram, tablets)	escitalopram	1
laxapro (escitalopram oxalate)	escitalopram oxalate	1
lexapro (escitalipram oxalate)	oxalate; escitalopram	1
lexapro (escitalopram olatate)	lexapro (escitalopram olatate)	1
lexprao (escitalopram oxalate)	lexprao (escitalopram oxalate)	1

cipralex(escitalopram oxalate)	escitalopram oxalate	1
lexapro (escitalorpam oxalate)	oxalate; escitalopram	1
lexapro (escitaloprma oxalate)	oxalate; escitalopram	1
meridian (escitalopram oxalate)	escitalopram oxalate	1
selectra [escitalopram oxalate]	selectra [escitalopram oxalate]	1
escitalopram 10mg tablet accord	escitalopram oxalate	1
escitalopram oxalate 5mg tablet	escitalopram oxalate	1
lexapro (escitalofpram oxalate)	oxalate; escitalopram	1
lexapro (escitalopram oxallate)	lexapro (escitalopram oxallate)	1
lexapro (escitalopram, oxalate)	escitalopram; oxalate	1
lexapro (escitalopramc oxalate)	oxalate; escitalopram	1
seroplex (escitalopram oxalale)	escitalopram;	1
lexapro (escitalo0pram oxalate)	oxalate; escitalopram	1
lexapro l(escitalopram oxalate)	escitalopram oxalate; escitalopram	1
lexapro (escitablopram oxalate)	oxalate; escitalopram	1
escitalopram 10mg forest pharm.	escitalopram	1
lkexapro (escitalopram oxalate)	escitalopram oxalate	1
lexpapro (escitalopram oxalate)	escitalopram oxalate	1
cital [escitalopram oxalate]	cital [escitalopram oxalate]	1
escitalopram generic for lexapro	escitalopram oxalate	1
escitalopram oxalate 5 mg tab ac	escitalopram oxalate	1
escitalopram 5 mg by mouth daily	escitalopram 5 mg by mouth daily	1
lexapro (escitalopram) (unknown)	escitalopram	1
seroplex film?coated tablets	escitalopram oxalate	1
escitalopram tab 20mg, 20 mg	escitalopram oxalate	1
escitalopram 15 mg 56 comprimidos	escitalopram oxalate	1
escitalopram, generic for lexapro	escitalopram oxalate	1
cipralex (unknown) (escitalopram)	cipralex (unknown) (escitalopram)	1
escitalopram 1mg/ml oral solution	escitalopram	1
escitalopram (cipralex) (unknown)	escitalopram	1
seroplex (escitalopram) (tablets)	seroplex (escitalopram) (tablets)	1
entact(escitalopram oxalate)	escitalopram oxalate	1
escitalopram (lexapro) 10 mg	escitalopram (lexapro) 10 mg	1
escitalopram, 20 mg teva usa	escitalopram oxalate	1
escitalopram 10mg aurobindo pharma	escitalopram oxalate	1
escitalopram; generic for: lexapro	escitalopram oxalate	1
escitalopram 20 mg accord healthca	escitalopram oxalate	1
seroplex 20 (escitalopram oxalate)	escitalopram;	1
escalipram -lexapro generic- 20 mf	escitalopram	1

escitaopram (escitalopram oxalate)	escitalopram oxalate	1
escitalopram (escitalpram oxalate)	oxalate; escitalopram	1
escitalpram (escitalopram oxalate)	escitalopram oxalate	1
escitalopram(escitalopram oxalate)	escitalopram	1
escitaloprm (escitalopram oxalate)	escitalopram oxalate	1
escitalopram mepha 5 mg	escitalopram oxalate	1
lisinopril,escitalopram	lisinopril,escitalopram	1
escitalopram (lexapro);	escitalopram (lexapro);	1
escitalopram teva 10 mg	escitalopram oxalate	1
escitaopram 10mg teva p	escitalopram oxalate	1
escitalopram (non teva)	escitalopram (non teva)	1
escitalopram (serodeps)	escitalopram (serodeps)	1
elicea (escitalopramum)	elicea (escitalopramum)	1
oxalato de escitalopram	oxalato de escitalopram	1
escitalopram 10mg mylan	escitalopram oxalate	1
maleato de escitalopram	escitalopram	1
lexapro or escitalopram	escitalopram	1
escitalopram 5 mg daily	escitalopram	1
cypralex (escitalopram)	cypralex (escitalopram)	1
escitalopram 20 mg teva	escitalopram	1
lexapro (escitalopram)q	escitalopram	1
lexapro (escitralopram)	escitalopram	1
lexaprol (escitalopram)	escitalopram	1
lexapro (escitalopramo)	escitalopram	1
ciplalex (escitalopram)	escitalopram	1
cirpalex (escitalopram)	escitalopram	1
escitalopram 10 mg, oral	escitalopram 10 mg, oral	1
escitalopram 40 mg daily	escitalopram 40 mg daily	1
escitalopram neuraxpharm	escitalopram oxalate	1
escitalopram tablet 15mg	escitalopram tablet 15mg	1
escitalopram (sipralexa)	escitalopram (sipralexa)	1
escitalopram drops 20 mg	escitalopram drops 20 mg	1
escitalopram, blue point	escitalopram oxalate	1
escitalopram 5mg walmart	escitalopram oxalate	1
escitalopram 10 mg lupin	escitalopram oxalate	1
escitalopram 20mg accord	escitalopram oxalate	1
cipralex (escitalopram)	escitalopram	1
escitalopram oxalate 3 d	escitalopram oxalate	1
cipralexv (escitalopram)	escitalopram	1

siraplexa (escitalopram)	escitalopram	1
spiralexa (escitalopram)	escitalopram	1
cipraxlex (escitalopram)	escitalopram	1
cipraflex (escitalopram)	escitalopram	1
serpoplex (escitalopram)	escitalopram	1
seroplex (escitalopram)	seroplex (escitalopram)	1
escitalopram 10mg forest	escitalopram	1
escitalopram 20mg tablets	escitalopram oxalate	1
escitalopram oxalate 10mg	escitalopram oxalate	1
escitalopram 10mg tablets	escitalopram 10mg tablets	1
stalopam film c.tabs 5 mg	escitalopram oxalate	1
escitalopram oxalate, 5mg	escitalopram oxalate	1
generic escitalopram 10.5	escitalopram oxalate	1
escitalopram tablets teva	escitalopram oxalate	1
escitalopram 10 mg tablet	escitalopram 10 mg tablet	1
escitalopram 10mg unknown	escitalopram oxalate	1
escitalopram-form_unknown	escitalopram oxalate	1
escitalopram 20mg torrent	escitalopram oxalate	1
lexapro (esopram oxalate)	oxalate; escitalopram	1
escitalopram 20 mg forest	escitalopram	1
lexapro unknown - unknown	escitalopram	1
lexapro / forest pharmace	escitalopram	1
escitalopram, 10 mg, teva	escitalopram	1
humalog (insulin lexapro)	escitalopram; insulin lispro	1
cipralex (escitalopram,)	escitalopram	1
escitalopram -lexapro(r)-	escitalopram	1
escitalopram 10 mg forest	escitalopram	1
escitalopram 20 mg po qday	escitalopram 20 mg po qday	1
escitalopram 10mg po daily	escitalopram 10mg po daily	1
escitalopram oxalate, 20mg	escitalopram oxalate	1
escitalopram, 10mg walmart	escitalopram oxalate	1
escitalopram 10 mg unknown	escitalopram oxalate	1
escitalopram 10 mg tablets	escitalopram 10 mg tablets	1
escitalopram, but/apap/caf	escitalopram, but/apap/caf	1
escitalopram (esitalopram)	escitalopram	1
escitalopam (escitalopram)	escitalopam (escitalopram)	1
escitalopram (escitalpram)	escitalopram	1
escitalopra (escitalopram)	escitalopram	1
escitaloram (escitalopram)	escitalopram	1

escitalpram (escitalopram)	escitalopram	1
escitalopram (escitalopram	escitalopram	1
esctalopram (escitalopram)	escitalopram	1
lexapro /usa/ escitalopram	escitalopram	1
escitalopream oxalate 10 mg	escitalopram oxalate	1
escitalopram genericon 20mg	escitalopram genericon 20mg	1
escitalopram bluefish 15 mg	escitalopram bluefish 15 mg	1
escitalopram oxalate accord	escitalopram oxalate	1
escitalopram(escitalop ram)	escitalopram	1
elicea/escitalopram oxalate	elicea/escitalopram oxalate	1
seroplex nos (escitalopram)	escitalopram;	1
lexapro (escitalpram oxate)	escitalopram	1
escitalopram oxalate (ssri)	escitalopram oxalate	1
escitalopram and clonazepam	escitalopram; clonazepam	1
cipralex (escitalopram, ,0)	escitalopram	1
escitalopram oxalate (con.)	escitalopram oxalate	1
esctialopram (escitalopram)	escitalopram	1
escitalopram oxaate	escitalopram oxaate	1
escitalopram, xanax	escitalopram, xanax	1
escitalopram tablet	escitalopram	1
lexapro (lorazepam)	escitalopram	1
escitalopram (5 mg)	escitalopram	1
oxalate escitalopram	escitalopram oxalate	1
act escitalopram odt	act escitalopram odt	1
escitalopram?tablets	escitalopram?tablets	1
lexapro escitalopram	lexapro escitalopram	1
lexopro escitalopram	escitalopram	1
escitalopram actavis	escitalopram	1
escitalopram heumann	escitalopram heumann	1
escitalopram beta 20	escitalopram beta 20	1
escitalopram (xanax)	escitalopram	1
lexapro 20 mg tablet	escitalopram	1
blinded escitalopram	escitalopram	1
xanax (escitalopram)	alprazolam	1
escitalopram tablets	escitalopram	1
lexapro (escitalop	escitalopram	1
escitalopram 5 mg tab	escitalopram 5 mg tab	1
escitalopram 20mg tab	escitalopram 20mg tab	1
levemir, escitalopram	levemir, escitalopram	1

scippa (escitalopram)	escitalopram oxalate	1
escitalopram 10mg tab	escitalopram 10mg tab	1
lexapro, escitalopram	lexapro, escitalopram	1
escitalopram (nexium)	escitalopram	1
lexapro oral solution	escitalopram	1
escitalopram 20mg aur	escitalopram oxalate	1
escitalopram oxaltate	escitalopram oxaltate	1
escitalopram(lexapro)	escitalopram(lexapro)	1
escitalopram winthrop	escitalopram winthrop	1
escitalopram tab 10mg	escitalopram	1
blinded: escitalopram	escitalopram	1
lexapro (escitalopam)	escitalopram	1
lexapro (escialopram)	escitalopram	1
lexapro (scitalopram)	escitalopram	1
lexapro (escitalpram)	escitalopram	1
lexaro (escitalopram)	escitalopram	1
lexapro (esitalopram)	escitalopram	1
lexpro (escitalopram)	escitalopram	1
escitalopram (ngx)	escitalopram	1
escitalopram 15 mg	escitalopram	1
lexyprol (lexapro)	escitalopram	1
escitalopram (invagen)	escitalopram oxalate	1
escitalopram 5 nightly	escitalopram 5 nightly	1
escitalopram (lexipro)	escitalopram (lexipro)	1
lexopro (escitalopram)	lexopro (escitalopram)	1
escitalopram (generic)	escitalopram oxalate	1
alprazolamescitalopram	alprazolamescitalopram	1
escitalopram abz 20 mg	escitalopram oxalate	1
lexapro (oscitalopram)	escitalopram	1
generic lexapro 2.5 mg	escitalopram oxalate	1
lexipro (escitalopram)	lexipro (escitalopram)	1
lexapro and bupropiron	escitalopram	1
escitalopram 20mg teva	escitalopram oxalate	1
nexito 10mg sun pharma	escitalopram oxalate	1
unknown (escitalopram)	escitalopram	1
escitalopram 5 mg teva	escitalopram	1
escitalopram - lexapro	escitalopram	1
escitalopram magnesium	magnesium; escitalopram	1
cipralex escitalopram)	escitalopram	1

escitalopram ({null}.)	escitalopram	1
lexapro (ssri) tablets	escitalopram	1
alopram (escitalopram)	escitalopram	1
lexapro (20 milligram)	escitalopram	1
cipralex (escitalopram	escitalopram	1
lexapro (con.0	escitalopram	1
escitalopramil	escitalopramil	1
escitalopram 20	escitalopram 20	1
thyroid lexapro	escitalopram	1
lexapro (40 mg)	escitalopram	1
ic-escitalopram	escitalopram	1
insulin lexapro	escitalopram	1
lexapro tablets	escitalopram	1
ach-escitolopram	escitalopram oxalate	1
escitalopram tam	escitalopram tam	1
escitalopram sol	escitalopram sol	1
ran?escitalopram	ran?escitalopram	1
escitalopram alm	escitalopram alm	1
escitalopram abc	escitalopram abc	1
escitalopram doc	escitalopram oxalate	1
pms escitalopram	escitalopram oxalate	1
apo?escitalopram	escitalopram oxalate	1
escitalopram 20g	escitalopram oxalate	1
lexapro 10mg bid	escitalopram	1
escitalopram-teva	escitalopram-teva	1
eccitalopram 10mg	escitalopram oxalate	1
ran? escitalopram	ran? escitalopram	1
escitalopram oxal	escitalopram oxal	1
escitalopram beta	escitalopram beta	1
lexapro (unknown)	escitalopram	1
escitalopram oral	escitalopram	1
escitalopram-20mg	escitalopram	1
escitalopram 5 mg	escitalopram	1
escitalopram, 20mg	escitalopram, 20mg	1
escitalopram mylan	escitalopram mylan	1
mylan escitalopram	mylan escitalopram	1
lexapro ni	escitalopram	1
stalopam 10	escitalopram oxalate	1
4-lexapro	escitalopram	1

lexapro 5 mg	escitalopram	1
heipram 10 mg	escitalopram oxalate	1
seroplex 5 mg	escitalopram oxalate	1
zytomil 10 mg	escitalopram oxalate	1
citanew	escitalopram oxalate	1
bivadin	escitalopram oxalate	1
escittab	escitalopram oxalate	1
saropram	escitalopram oxalate	1
s citadep	escitalopram oxalate	1
cipralex (escitalopram oxalate), unknown	escitalopram oxalate	1
escitalopram oxelate	escitalopram	1
escitalopram 20mg (1.5 tablets qd)	escitalopram	1
escitalopram 15 mg comprimido	escitalopram	1
lexapro 20mg tablets	escitalopram	1
esc (escitalopram oxalate)	escitalopram oxalate	1
escitalopram, fosamax, lasix, tikosyn, lipitor, nitrostat, proventil	escitalopram; alendronate; furosemide; dofetilide; atorvastatin; glyceryl trinitrate; salbutamol	1
escitalopram actavis 10 mg filmdragerad tablett	escitalopram oxalate	1
ach escitalopram	escitalopram	1
escitalopram ranbaxy	escitalopram oxalate	1
warfarin, lamictal, lexapro, lisinopril, potassium chloride, klonopin	warfarin; lamotrigine; escitalopram; lisinopril; potassium chloride; clonazepam	1
escitalopram almus 20 mg	escitalopram	1
escitalopram, hydromet	escitalopram; hydrocodone	1
escitalopram (lexapro) 20mg	escitalopram	1
escitalopram-mepha	escitalopram oxalate	1
lexapro, synthroid, mycophenolate, oxaprozin, aspirin low dose	escitalopram; levothyroxine; mycophenolate; oxaprozin; aspirin low dose	1
cipralex [escitalopram	escitalopram	1
escitalopram oxalate 20 mg oral at bedtime	escitalopram	1
escitalopram/sandoz	escitalopram	1
giachela	escitalopram oxalate	1
escitalopram 20mg qd	escitalopram	1
escitalopram sandoz 10 mg, filmdragerade tabletter	escitalopram oxalate	1
escitalopram 2.5mg	escitalopram	1
escitalopram sandoz 10 mg filmdragerad tablett	escitalopram oxalate	1
heipram 10 mg comprimidos recubiertos con pelicula efg, 28 comprimidos	escitalopram oxalate	1
escitalopram oxalate 10mg, oral	escitalopram	1

lexapro 20 mg, oral	escitalopram	1
escitalopram-mepha 10mg	escitalopram oxalate	1
clonazepam;escitalopram oxalate	clonazepam; escitalopram oxalate	1
escitalopram?mepha 10mg	escitalopram oxalate	1
escitalopram 20 mg tab	escitalopram	1
escitalopram, pantoprazole, levothyroxine, clopidogrel, divalproex	escitalopram; pantoprazole; levothyroxine; clopidogrel; valproic acid	1
escitalopram tablet 20mg / brand name not specified	escitalopram	1
lexapro ssri	ssri; escitalopram	1
escitalopram tablet 15mg / brand name not specifiedescitalopram tab.	escitalopram	1
lexapro (non?abbvie)	escitalopram	1
super b, vistaril, garlic, wellbutrin, fish oil, lexapro, escitalopram	super b; hydroxyzine; bupropion; fish oil; escitalopram	1
lexapro 10mg po daily	escitalopram	1
escitalopram?generic	escitalopram oxalate	1
escitalopram 10mg tablets, mfg aurobindo	escitalopram oxalate	1
lexapro 5mg qhs	escitalopram	1
escitalopram 10 mg generic for: lexapro10 mg	escitalopram oxalate	1
escitalopram?ratiopharm 10mg filmtabletten	escitalopram oxalate	1
escitalopram 10mg (generic lexapro)	escitalopram oxalate	1
escitalopram kariva	kariva; escitalopram	1
lexapro 10mg tablet	escitalopram	1
hydrochlorothiazide, atorvastatin, gabapentin, escitalopram, etodolac	hydrochlorothiazide; atorvastatin; gabapentin; escitalopram; etodolac	1
escitalopram oxalate 5mg	escitalopram oxalate	1
multivitamin, synthroid, lexapro, probiotic, plavix, pantoprazole	multiple vitamins; levothyroxine; escitalopram; clopidogrel; pantoprazole	1
escitalopram oxalate 10 mg tab sub for lexapro	escitalopram oxalate	1
lexapro 20 mg tablets	escitalopram	1
escitalopram oxalate 20 mg tablet sub for lexapro	escitalopram oxalate	1
escitalopram?mepha tropfen	escitalopram oxalate	1
escitalopram tablet 10mg / lexapro tablet filmomhuld 10mg	escitalopram	1
lexipro/escitalopram	escitalopram	1
escitalopram biogaran 15 mg, comprime pellicule secable	escitalopram oxalate	1
escitalopram teva 10 mg, comprime pellicule secable	escitalopram oxalate	1
escitalopram heumann 15 mg filmtabletten	escitalopram oxalate	1
ecitalopram (escitalopram oxalate 5mg tab)	escitalopram oxalate	1

escitalopram tablet 20 mg	escitalopram oxalate	1
lexapro 15 mg	escitalopram	1
escitalopram arrow film?coated tablet 20mg	escitalopram oxalate	1
escitalopram oxalate 5 mg/5 ml	escitalopram	1
escitalopram azevedos	escitalopram	1
lexapro seroquel	quetiapine; escitalopram	1
lexapro 20 mg po	escitalopram	1
escitalopram oxalate (lexapro) 20 mg: take 1 tablet po qd	escitalopram	1
escitalopram hb	escitalopram	1
escitalopram 20mg po qam	escitalopram	1
escitalopram - 10 mg	escitalopram	1
escitalopram 20mg po daily	escitalopram	1
escitalopram / lexapro	escitalopram	1
escitalopram 20 mg po	escitalopram	1
escitalopram (non-abbvie)	escitalopram	1
lexapro sol	escitalopram	1
lexapro tab 10 mg	escitalopram	1
escitalopram (lexapro) 10 mg tablet	escitalopram	1
escitalopram 10mg, rosuvastatin 20mg	escitalopram; rosuvastatin	1
ssri lexapro	escitalopram; ssri	1
lexapro and xolair	escitalopram; omalizumab	1
escitalopram oxalate oral	escitalopram oxalate	1
amlodipine hydrochlorithiazide lexapro	escitalopram; amlodipine	1
exodus (oxalate of escitalopram)	escitalopram; oxalate; pyrantel	1
lexapro 30mg daily	escitalopram	1
lexapro 5mg once daily	escitalopram	1
escitalopram 5 mg po qd	escitalopram	1
escitalopram meiji	escitalopram	1
lexapro 10mg po qam	escitalopram	1
lexapro 10mg po qday	escitalopram	1
escitalopram leugim	escitalopram	1
escitalopram 10 mg id	escitalopram	1
esitopam [escitalopram]	escitalopram	1
lexapro 10mg tablets	escitalopram	1
escitalopram 5mg po qd	escitalopram	1
escitalopram (10mg)	escitalopram	1
escitalopram almus 5 mg, comprime pellicule	escitalopram	1
escitalopram (lupin)	escitalopram	1

escitalopram 1a pharma	escitalopram	1
lexapro zomg tablets	escitalopram	1
norvasc escitalopram	escitalopram; amlodipine	1
lexapro loratadine	loratadine, micronized; escitalopram	1
lexapro oral tablet 10 mg	escitalopram	1
lexapro 25 mg	escitalopram	1
escitalopram oxalate 10 mg tablet	escitalopram	1
escitalopram oxalate escitalopram 20mg	escitalopram; escitalopram oxalate	1
estalo (escitalopram)	escitalopram	1
lexapro 5 mg tablet	escitalopram	1
lexapro 20 mg daily	escitalopram	1
lexapro gabapentin	gabapentin; escitalopram	1
lexapro 5	escitalopram	1
escitalopram oxalate 20 mg	escitalopram	1
5mg lexapro	escitalopram	1
10 mg escitalopram oxalate	escitalopram	1
escitalopram x 10 mg	escitalopram	1
escitalopram 30 mg	escitalopram	1
escitalopram oxalate 10 mc tablet	escitalopram	1
10 mg lexapro	escitalopram	1
lexapro 5 mg daily	escitalopram	1
escitalopram oxalate 20mg	escitalopram	1
celopram [escitalopram oxalate]	escitalopram oxalate	1
lexapro- 5 mg	escitalopram	1
lexapro (10mg)	escitalopram	1
lexapro for anxiety	escitalopram	1
talopram [escitalopram oxalate]	escitalopram oxalate	1
escitalopram oxalate (10 mg)	escitalopram	1
escitalopram abz	escitalopram	1
np thyroid lexapro wellbutrin linzess	linaclotide; bupropion; escitalopram; np thyroid	1
lexapro, 10mg	escitalopram	1
10 mg sandoz escitalopram	escitalopram	1
seroplex 20 mg, scored film-coated tablet [es-citalopram oxalate]	; escitalopram oxalate	1
escitalopram heipram	escitalopram	1
lexapram [escitalopram oxalate]	escitalopram oxalate	1
escitalopram oxalate 20mg po daily	escitalopram	1
escitalopram (lexapro) 20 mg tablet	escitalopram	1
escitalopram flas cinfa	escitalopram	1

escitalopram [escitalopram]	escitalopram	1
escitalopram oxalate [escitalopram oxalate]	escitalopram oxalate	1
escitalopram 20 mg po daily	escitalopram	1
escitalopramescitalopram		1
escitaloprams		1
escitalopram10mg/day		1
kescitalopram		1

Table S4. The dictionary used for filtering Fluoxetine was applied.

**drugname(129979)**	**prod_ai**	**AE**
prozac	fluoxetine hydrochloride	55637
fluoxetine	fluoxetine\fluoxetine hydrochloride; fluoxetine hydrochloride	53265
fluoxetine hcl	fluoxetine hydrochloride	6786
fluoxetine hydrochloride.	fluoxetine hydrochloride	3283
fluoxetine hydrochloride	fluoxetine hydrochloride	2304
symbyax	fluoxetine hydrochloride\olanzapine	1362
fluoxetin	fluoxetine hydrochloride	856
fluctine	fluoxetine hydrochloride	386
fluoxetine (fluoxetine)	fluoxetine hydrochloride	379
deprax	trazodone hydrochloride; fluoxetine hydrochlo-ride	324
sarafem	fluoxetine hydrochloride	301
fluoxetine hcl unk	fluoxetine hydrochloride	273
fluoxetine oral solution usp	fluoxetine\fluoxetine hydrochloride; fluoxetine hydrochloride	223
prozac (fluoxetine hydrochloride)	prozac (fluoxetine hydrochloride)	181
lovan	fluoxetine hydrochloride	181
fluoxetina	fluoxetine hydrochloride	176
prozac weekly	fluoxetine hydrochloride	130
daforin	fluoxetine hydrochloride	113
fluoxetine (unknown)	fluoxetine	105
fluoxetine (chlorhydrate de)	fluoxetine hydrochloride	101
fluoxetine capsules, usp	fluoxetine hydrochloride	96
fontex	fluoxetine hydrochloride	92
ladose	fluoxetine hydrochloride	92
fluoxetine hydrochloride (unknown)	fluoxetine hydrochloride	88
fluoxetine (watson laboratories)	fluoxetine hydrochloride	86
seronil	fluoxetine hydrochloride	67

zyprexa/symbyax	fluoxetine; olanzapine	65
fluctin	fluoxetine hydrochloride	55
nuzak	fluoxetine hydrochloride	54
fluoxetine capsules usp 20 mg	fluoxetine hydrochloride	50
fluoxeren	fluoxetine hydrochloride	50
fluoxetine arrow	fluoxetine hydrochloride	49
apo-fluoxetine	fluoxetine hydrochloride	47
deprexetin	fluoxetine hydrochloride	46
zyprexa/symbyax (olanzapine)	fluoxetine; olanzapine	42
floxetine	fluoxetine hydrochloride	40
olanzapine + fluoxetine hcl	olanzapine+fluoxetine	39
fluoxetine /n/a/	fluoxetine /n/a/	39
fluoxetine hydrochloride (non-specific)	fluoxetine hydrochloride	38
fluxotine	fluoxetine hydrochloride	36
fluoxetine (fluoxetine hydrochloride)	fluoxetine	35
fluoxetine teva	fluoxetine hydrochloride	34
fluoxetine hydrochloride (fluoxetine hydrochloride)	fluoxetine hydrochloride	32
fluoxetine (fluoxetine) (fluoxetine)	fluoxetine	32
prozac (fluoxetine)	prozac (fluoxetine)	31
norfluoxetine	norfluoxetine	31
serdep	sertraline hydrochloride; fluoxetine hydrochloride	31
lorien	fluoxetine hydrochloride	27
prozac (fluoxetine hydrochloride) (fluoxetine hydrochloride)	fluoxetine hydrochloride; fluoxetine	25
fluoxetine 40mg	fluoxetine hydrochloride	25
flouxetine	fluoxetine hydrochloride	25
fluxene	fluoxetine hydrochloride	25
fluoxetine (fluoxetine	fluoxetine	22
generic prozac	fluoxetine hydrochloride	22
fluoxetine hydrochloride (watson laboratories)	fluoxetine hydrochloride	20
fluoxetine dr	fluoxetine dr	20
fluoxetine mylan	fluoxetine hydrochloride	20
fluoxetine 10 mg	fluoxetine hydrochloride	20
fluoxetine hci	fluoxetine hydrochloride	19
sarafem (fluoxetine)	fluoxetine	18
bioxetin	fluoxetine hydrochloride	18
fluoxetine 20mg capsules	fluoxetine hydrochloride	17
olanzapine and fluoxetine	fluoxetine hydrochloride\olanzapine	17

fluoxetin 1a pharma	fluoxetine hydrochloride	17
fluoxetine hcl (fluoxetine hydrochloride)	fluoxetine hcl	16
fluoxetine hydrochloride 20mg	fluoxetine hydrochloride	16
adofen	fluoxetine hydrochloride	16
fluoxetine (prozac)	fluoxetine (prozac)	15
fluoxetine/fluoxetine hydrochloride	fluoxetine hydrochloride	14
fluoxetine hydrochloride oral solution	fluoxetine	14
fluoxetine (fluoxetine) unknown	fluoxetine hydrochloride	14
fluoxetina [fluoxetine]	fluoxetine hydrochloride	14
fluoxetine [fluoxetine hydrochloride]	fluoxetine hydrochloride	13
lovan (fluoxetine hydrochloride)	fluoxetine hydrochloride	12
prozac (fluoxetine hcl)	prozac (fluoxetine hcl)	12
chlorhydrate de fluoxetine	fluoxetine hydrochloride	12
fluoxetine (fluoxetine) capsule	fluoxetine	11
fluoxetine chlorhydrate	fluoxetine chlorhydrate	11
fluoxetina (2331a)	fluoxetine hydrochloride	11
ranflocs	fluoxetine hydrochloride	11
fluoxetin (fluoxetine hydrochloride)	fluoxetin (fluoxetine hydrochloride)	10
fluoxetine arrow capsule, hard 20 mg	fluoxetine hydrochloride	10
fluoxetine hydrochloride tablets	fluoxetine hydrochloride tablets	10
fluoxetine w/olanzapine	fluoxetine w / olanzapine	10
fluoxetine (r-fluoxetine)	fluoxetine hydrochloride	10
fluoxetine (ngx)	fluoxetine hydrochloride	10
fluoxetine hydrochloride (unknown) (fluoxetine hydrochloride) unk, unkunk	fluoxetine hydrochloride	9
prozac (fluoxetine hydrochloride) (unknown)	fluoxetine	9
olanzipine/fluoxetine capsule	fluoxetine	9
fluoxetine /n/a/ (fluoxetine)	fluoxetine	9
serdep (fluoxetine hydrochloride)	fluoxetine hydrochloride	9
fluoxetin axapharm	fluoxetine hydrochloride	9
fluoxetine oral solution usp, 20mg/5ml (alpharma) (fluoxetine)	fluoxetine	8
fluoxetine tablet, 20 mg (milligram)	fluoxetine hydrochloride	8
fluoxetine hydrochloride 20 mg capsule	fluoxetine hydrochloride	8
prozac (con.)	fluoxetine	8
pms-fluoxetine	fluoxetine hydrochloride	8
fluoxetine 60mg	fluoxetine hydrochloride	8
flux (fluoxetine)	flux (fluoxetine)	8
fluoxetin actavis	fluoxetine hydrochloride	8

fluctine 20mg	fluoxetine hydrochloride	8
lovan (fluoxetine hydrochloride) (20 mg)	fluoxetine	7
prozac (fluoxetine hydrochloride) tablets	fluoxetine	7
prozac (fluoxetine hydrochloride) (capsules)	fluoxetine	7
symbyax (fluoxetine, olanzapine)	fluoxetine; olanzapine	7
depress/fluoxetine hydrochloride	depress/fluoxetine hydrochloride	7
deprax (fluoxetine hydrochloride)	fluoxetine hydrochloride	7
deprax [fluoxetine hydrochloride]	fluoxetine hydrochloride	7
fluoxetine 40mg capsules	fluoxetine hydrochloride	7
fluoxetin teva	fluoxetine hydrochloride	7
fluoxetin mepha	fluoxetine hydrochloride	7
ladose 20mg	fluoxetine hydrochloride	7
fluoxetine (formulation unknown) (generic) (fluoxetine)	fluoxetine	6
fluoxetine hydrochloride teva	fluoxetine hydrochloride	6
prozac (fluoxetine) (fluoxetine)	fluoxetine	6
bristol labs fluoxetine	bristol labs fluoxetine	6
fluoxetine capsules	fluoxetine	6
olanzapine/fluoxetine	fluoxetine hydrochloride\olanzapine	6
fluoxetine eg	fluoxetine eg	6
pro-fluoxetine	pro-fluoxetine	6
fluoxetine (con.)	fluoxetine	6
portal	fluoxetine hydrochloride	6
fluoxetine oral solution usp, 20mg/5ml (alpharma)	fluoxetine	5
prednisone, famotidine, fluoxetine, acetaminophen (formula)	prednisone; famotidine; fluoxetine; acetaminophen	5
fluoxetina combix 20 mg capsulas duras efg	fluoxetine hydrochloride	5
fluoxetine molecule from uk market	fluoxetine hydrochloride	5
accord healthcare fluoxetine	fluoxetine hydrochloride	5
fluoxetine capsule 10mg,20mg	fluoxetine	5
zyprexa /symbyax (olanzapine)	fluoxetine; olanzapine	5
fluoxetine hydrochloride sandoz	fluoxetine hydrochloride	5
fluoxetine (fluoxetine) (unknown)	fluoxetine (fluoxetine) (unknown)	5
fluoxetine oral solution	fluoxetine hydrochloride	5
fluoxetin biochemie	fluoxetine\fluoxetine hydrochloride; fluoxetine hydrochloride	5
fluoxetine/olanzapine	fluoxetine hydrochloride\olanzapine	5
fluoxetine(fluoxetine)	fluoxetine(fluoxetine)	5
fluoxetin-mepha	fluoxetine hydrochloride	5
hydroxyzyna	fluoxetine hydrochloride	5

prohexal	fluoxetine hydrochloride	5
pms?fluoxetine	fluoxetine hydrochloride	5
act fluoxetine	fluoxetine hydrochloride	5
accel fluoxetine	fluoxetine	5
fluoxetine hydrochloride (fluoxetine hydrochloride unknown formulation	fluoxetine hydrochloride	4
fluctine (fluoxetine hydrochloride)	fluoxetine hydrochloride	4
12mg/50mg olanzipine/fluoxetine capsule	fluoxetine	4
olanzapine/fluoxetine capsule	fluoxetine	4
fluoxetine mylan 20 mg, g?lule	fluoxetine hydrochloride	4
prozac (fluoxetin hydrochloride)	hydrochloride; fluoxetine	4
fontex (fluoxetine hydrochloride)	fluoxetine hydrochloride	4
fluoxetin 10 - 1 a pharma	fluoxetine hydrochloride	4
fluoxetin 1a pharma (ngx)	fluoxetine	4
seronil (fluoxetine) 20 mg	fluoxetine hydrochloride	4
generic fluoxetine	fluoxetine hydrochloride	4
fluoxetin hexal	fluoxetine	4
flouxetine 20mg	fluoxetine hydrochloride	4
fluoxetine 20mg	fluoxetine	4
fluoxetina davur	fluoxetine hydrochloride	4
ratio-fluoxetine	fluoxetine	4
fluoxetine mepha	fluoxetine hydrochloride	4
ratio fluoxetine	fluoxetine	4
sandoz fluoxetine	fluoxetine hydrochloride	4
fluoxetine sandoz	fluoxetine	4
fluoxetine h	fluoxetine h	4
fluoxetin 20	fluoxetine hydrochloride	4
fluoxetin ratipharm	fluoxetine hydrochloride	4
fluoxetine capsule 20mg / brand name not specified	fluoxetine hydrochloride	4
fluoxetine dispertablet 20mg / brand name not speci-fied	fluoxetine hydrochloride	4
fluoxetine chloridate	fluoxetine	4
fluoxetine hcl (fluoxetine hydrochloride) (unknown)	fluoxetine hcl	3
fluoxetine+ unknown drugs (magistral formula)	fluoxetine	3
fluoxetine /n/a/ (fluoxetine /n/a/)	fluoxetine	3
fluoxetine (ngx) (fluoxetine) tablet	fluoxetine	3
fluoxetina (fluoxetine hydrochloride)	fluoxetina (fluoxetine hydrochloride)	3
fluoxetine hcl(fluoxetine hydrochloride)	fluoxetine hcl(fluoxetine hydrochloride)	3
prozac (fluoxetine hydrochloride) capsule	fluoxetine	3

meloxicam/deflazacort/famotidine/fluoxetine	famotidine/ibuprofen; meloxicam	3
fluoxetine hydrochloride 90mg	fluoxetine hydrochloride	3
fluoxeren 20 mg capsule rigide	fluoxetine hydrochloride	3
luoxetine (presumed fluoxetine)	fluoxetine	3
fluoxetine hydrochloride (trizac)	fluoxetine hydrochloride (trizac)	3
proxac (fluoxetine hydrochloride)	fluoxetine hydrochloride	3
fluoxetine hcl (prozac)	fluoxetine hcl (prozac)	3
fluoxetine hcl 20mg caps	fluoxetine hydrochloride	3
cloridrato de fluoxetina	fluoxetine hydrochloride	3
fluoxetine 10mg capsules	fluoxetine hydrochloride	3
fluoxetine 20 mg tablets	fluoxetine	3
fluoxetine 40 mg capsules	fluoxetine hydrochloride	3
fluoxetin ratiopharm 20 mg	fluoxetine hydrochloride	3
fluoxetin 1a farma	fluoxetine hydrochloride	3
fluoxetine hcl 20mg	fluoxetine hydrochloride	3
fluoxetine winthrop	fluoxetine hydrochloride	3
fluoxetine cap 20mg	fluoxetine cap 20mg	3
fluoxetine dr reddys	fluoxetine dr reddys	3
seronil (fluoxetine)	fluoxetine hydrochloride	3
fluoxetine 40mg teva	fluoxetine	3
fluoxetine hcl (pmdd)	fluoxetine hcl (pmdd)	3
fluoxetine 20mg daily	fluoxetine 20mg daily	3
olanzipine/fluoxetine	fluoxetine	3
fluoxetine 20 mg, cap	fluoxetine	3
fluoxetine cap	fluoxetine cap	3
zyprexa symbyax	fluoxetine; olanzapine	3
fluoxetin 20 mg	fluoxetine hydrochloride	3
novo-fluoxetine	fluoxetine hydrochloride	3
teva fluoxetine	fluoxetine hydrochloride	3
mylan-fluoxetine	fluoxetine hydrochloride	3
fluoxetina 20 mg	fluoxetine hydrochloride	3
fluoxetin sandoz	fluoxetine hydrochloride	3
prozac/fluoxetine	prozac/fluoxetine	3
fluoxetin ?mylan?	fluoxetine hydrochloride	3
prozac(fluoxetine)	prozac(fluoxetine)	3
fluxantin	fluoxetine hydrochloride	3
prozac 20mg	fluoxetine	3
prozep	fluoxetine hydrochloride	3
prizma	fluoxetine hydrochloride	3

deprex [fluoxetine hydrochloride]	fluoxetine hydrochloride	3
fluoxetin 20mg	fluoxetine hydrochloride	3
alzac [fluoxetine]	fluoxetine	3
prozac 20mg daily	fluoxetine	3
fluoxetin(fluoxetine hydrochloride)	fluoxetine	3
fluoxetine tab	fluoxetine	3
prozac (fluoxetine hydrochloride) capsule, 20mg	fluoxetine	2
sarafem (fluoxetine hydrochloride) tablet, 20mg	fluoxetine	2
fluoxetine hcl capsules usp, 10mg, 20 mg, 40 mg	fluoxetine hydrochloride	2
prozac (fluoxetine hydrochloride) capsule, 10 mg	fluoxetine	2
prozac (fluoxetine hydrochloride) (10 milligram)	fluoxetine	2
fluoxetine hydrochloride(fluoxetine hydrochloride)	fluoxetine hydrochloride(fluoxetine hydrochloride)	2
fluctin-oral (fluoxetine) (fluoxetine hydrochloride	fluoxetine hydrochloride; fluoxetine	2
prozac (fluoxetine hydrochloride unknown formulation)	fluoxetine hydrochloride; fluoxetine	2
fluoxetine (r-fluoxetine) (fluoxetine (r-fluoxetine))	fluoxetine	2
fluoxetine (fluoxetine hydrochloride unknown manufacturer)	fluoxetine hydrochloride; fluoxetine	2
prozac (fluoxetine hydrochloride) (fluoxetine hydrochloride0	fluoxetine	2
prozac (fluoxetine hydrochloride) (fluoxetine hydrohcloride)	fluoxetine	2
olanzapine/fluoxetine hydrochloride (fluoxetine hydrochloride, olanzap	fluoxetine hydrochloride; olanzapine / fluoxetine hydrochloride	2
fluoxetine 10mg cap generic for, 10 mg 68645-0131-54 fluoxetine 10mg cap	fluoxetine hydrochloride	2
fluoxetine hydrochloride (unknown) (fluoxetine hydrochloride) unk, unk unk	fluoxetine hydrochloride	2
fluoxetin-ratiopharm 20 mg tabletten	fluoxetine hydrochloride	2
fluoxetine sandoz (ngx) (fluoxetine)	fluoxetine	2
fluoxetine hcl 40 mg capsule teva usa	fluoxetine hydrochloride	2
fluoxibene (fluoxetine hydrochloride)	fluoxetine hydrochloride	2
fluoxetine (fluoxetine hydrochloride)	fluoxetine (fluoxetine hydrochloride)	2
fluoxetine 20mg generic through walmart	fluoxetine hydrochloride	2
olanzapine and fluoxetine hydrochloride	fluoxetine hydrochloride\olanzapine	2
fluoxetina ratiopharm - ratiopharm gmbh	fluoxetine hydrochloride	2
lovan (60 mg) (fluoxetine hydrochloride)	fluoxetine	2
oxetine (fluoxetine hydrochloride)	fluoxetine hydrochloride	2
fluoxetine aurobindo 20 mg capsules, hard	fluoxetine hydrochloride	2
fluoxetina cinfa 20 mg capsulas duras efg	fluoxetine hydrochloride	2
fluoxetine arrow 20 mg dispersible tablet	fluoxetine hydrochloride	2

fluctin (fluoxetine hydrochloride) capsule	fluctin (fluoxetine hydrochloride) capsule	2
fluctine (fluoxetine hydrochloride) tablet	fluctine (fluoxetine hydrochloride) tablet	2
prozac (fluoxetine hydrochloride) capsules	fluoxetine	2
ladose (fluoxetine hydrochloride) solution	ladose (fluoxetine hydrochloride) solution	2
fluoxetin beta 40, tabletten	fluoxetine	2
fluoxetine hci oral solution	fluoxetine hydrochloride	2
fluoxetine /n/a/(fluoxetine)	fluoxetine	2
sarafem (fluoxetine) capsules	fluoxetine	2
fluoxetine oral solution usp,	fluoxetine	2
fluoxetine capsule, hard 20 mg	fluoxetine hydrochloride	2
prozac (40 milligram, tablets)	fluoxetine	2
prozac fluoxetine hydrochloride	fluoxetine hydrochloride; fluoxetine	2
flux [fluoxetine hydrochloride]	fluoxetine hydrochloride	2
olanzapine/fluoxetine [symbyax]	fluoxetine; olanzapine / fluoxetine	2
fluoxetine hydrochloride(trizac)	fluoxetine hydrochloride(trizac)	2
serdep [fluoxetine hydrochloride]	serdep [fluoxetine hydrochloride]	2
portal [fluoxetine hydrochloride]	fluoxetine hydrochloride	2
zactin (fluoxetine hydrochloride)	fluoxetine hydrochloride	2
fluoxetine (fluoxetine 00724401/)	fluoxetine	2
prozac 9fluoxetine hydrochloride)	hydrochloride; fluoxetine	2
prozac (flouxetine hydrochloride)	hydrochloride; fluoxetine	2
fluctin [fluoxetine hydrochloride]	fluoxetine hydrochloride	2
fluoxetine sol 20mg/5ml	fluoxetine sol 20mg/5ml	2
fluoxetine 20mg tablets	fluoxetine 20mg tablets	2
fluoxetine (fluoxetine)	fluoxetine (fluoxetine)	2
auro-fluoxetine capsules	fluoxetine hydrochloride	2
generic prozac fluoxetine	generic prozac fluoxetine	2
fluoxetine (40 milligram)	fluoxetine	2
fluoxetine tablets, 20 mg	fluoxetine hydrochloride	2
fluoxetine hcl teva pharm	fluoxetine hydrochloride	2
fluoxetini hydrochloridum	fluoxetine hydrochloride	2
fluoxetine capsules (ngx)	fluoxetine	2
fluoxetine chloridrate	fluoxetine	2
teva pharma fluoxetine	fluoxetine hydrochloride	2
fluctine 20mg capsules	fluoxetine hydrochloride	2
fluoxetine (fluxetine)	fluoxetine	2
fluoxetine chlorhydrate de	fluoxetine hydrochloride	2
fluoxetine 20 mg aurobindo	fluoxetine hydrochloride	2
gerozac 20mg capsules hard	fluoxetine\fluoxetine hydrochloride	2

fluoxetin (fluoxetine)	fluoxetine	2
fluoxetine hcl 10 mg tablet	fluoxetine hydrochloride	2
fuoxetine (fluoxetine)	fluoxetine	2
blinded fluoxetine	fluoxetine hydrochloride	2
fluoxetine solution	fluoxetine hydrochloride	2
ladose (fluoxetine)	fluoxetine	2
fluoxetine tab 20 mg	fluoxetine tab 20 mg	2
fluoxetine cap 20 mg	fluoxetine cap 20 mg	2
fluoxetine cap 40 mg	fluoxetine cap 40 mg	2
fluoxetine hcl 20 mg	fluoxetine hydrochloride	2
fluoxetine usp - ipi	fluoxetine	2
fluoxetine hci (pmdd)	fluoxetine hci (pmdd)	2
fluoxetine hcl/prozac	fluoxetine; fluoxetine hcl	2
actavis uk fluoxetine	actavis uk fluoxetine	2
fluoxetine 40mg daily	fluoxetine 40mg daily	2
fluoxetine 10mg, oral	fluoxetine 10mg, oral	2
fluoxetina ratiopharm	fluoxetine\fluoxetine hydrochloride; fluoxetine hydrochloride	2
fluoxetin, 20mg, tabl	fluoxetine	2
fluoxetine hcl (pmdd)m	fluoxetine hcl (pmdd)m	2
fluoxetine hcl/ prozac	fluoxetine; fluoxetine hcl	2
prozac/ fluoxetine hcl	fluoxetine hcl; fluoxetine	2
fluoxetin beta	fluoxetine hydrochloride	2
fluoxitine 20mg	fluoxetine hydrochloride	2
fluoxetine-auro	fluoxetine hydrochloride	2
fluoxetine 40 mg	fluoxetine	2
fluoxetine alter	fluoxetine	2
fluoxetine 80 mg	fluoxetine 80 mg	2
fluoxetine 60 mg	fluoxetine 60 mg	2
genrx fluoxetine	fluoxetine	2
fluoxetine lofen	fluoxetine	2
fluoxetine stada	fluoxetine	2
fluoxetine 20 ml	fluoxetine	2
fluoxetine/prozac	fluoxetine/prozac	2
fluoxetina tablet	fluoxetine hydrochloride	2
gaboxetine	choline\fluoxetine hydrochloride	2
prozac 10mg	fluoxetine	2
prozac 20 mg	fluoxetine	2
fluval	fluoxetine hydrochloride	2

fluxet	fluoxetine hydrochloride	2
fluoxetine 80mg	fluoxetine	2
fluoxetin?mepha	fluoxetine hydrochloride	2
fluoxetine hcl 10 mg		2
prazac [fluoxetine hydrochloride]	fluoxetine hydrochloride	2
prozac 30mg	fluoxetine	2
olanzapine?fluoxetine hcl	fluoxetine hcl; olanzapine	2
fluoxetine oral solution usp, af df	fluoxetine hydrochloride	2
fluoxetine vitabalans	fluoxetine	2
pro?fluoxetine	fluoxetine	2
fluoxeren 20 mg/5 ml soluzione orale	fluoxetine hydrochloride	2
fluoxetine tablets usp, 60 mg	fluoxetine hydrochloride	2
reneuron [fluoxetine hydrochloride]	fluoxetine hydrochloride	2
fluoxetine hydrochloride (non?specific)	fluoxetine hydrochloride	2
fluoxetine hydrochloride;olanzapine	fluoxetine hydrochloride; olanzapine	2
prozen [fluoxetine hydrochloride]	fluoxetine hydrochloride	2
fluoxetine/fluoxetine hcl	fluoxetine hcl; fluoxetine	2
luminal [fluoxetine hydrochloride]	fluoxetine hydrochloride	2
fluoxetine [fluoxetine]		2
fluoxetinee hcl. 10 mg capsule		2
fluoxetine (fluoxetine) (unknown) (fluoxetine)	fluoxetine	1
seronil (fluoxetine) 20 mg (fluoxetine) uknown	fluoxetine hydrochloride	1
prozac (fluoxetine hydrochloride) (fluoxetine)	prozac (fluoxetine hydrochloride) (fluoxetine)	1
fluoxetine capsules (fluoxetine) capsule, 10mg	fluoxetine	1
symbyax (fluoxetine hydrochloride, olanzapine)	fluoxetine hydrochloride; fluoxetine; olanzap-ine	1
fluoxetin biochemie (fluoxetine hydrochloride)	fluoxetine hydrochloride; fluoxetine	1
sarafem-pmdd (premenstrual dysphoric disorder)	fluoxetine	1
fluoxetine (fluoxetine) (40 milligram, tablets)	fluoxetine	1
fluoxeteine (fluoxetine) (unknown) (fluoxetine)	fluoxetine	1
prozac (fluoxetine hydrochloride) capsule, 10mg	fluoxetine	1
symbyax (fluoxetine hydrochloride w/olanzapine)	fluoxetine	1
fluoxetine (fluoxetine) (40 milligram, unknown)	fluoxetine	1
fluoxetin ratiopharm (fluoxetine hydrochloride)	fluoxetine hydrochloride; fluoxetine	1
symbax-olanzapine 12mg/fluoxetine 25mg(olanzapi	fluoxetine; olanzapine	1
fluoxetine (fluoxetine hydrochloride) (capsules)	fluoxetine	1
sarafem (fluoxetine hydrochloride) tablet, unkmg	fluoxetine	1
prozac weekly (fluoxetine hydrochloride) capsule	fluoxetine	1
fluoxetine acetate oral solution, 20 mg/5ml, par	acetate salt; fluoxetine	1

prozac (fluoxetine hydrochloride) (20 milligram)	fluoxetine	1
symbyax-olanzapine 6mg/fluoxetine 25mg(olanzapin	fluoxetine; olanzapine	1
fluoxetine hcl (fluoxetine hydrochloride) capsule	fluoxetine hcl (fluoxetine hydrochloride) cap-sule	1
symbyak (olanzapine and fluoxetine hydrochloride)	olanzapine; fluoxetine hydrochloride	1
symbyax (olanzapine and fluoxetine hydrochloride)	olanzapine; fluoxetine; fluoxetine hydrochlo-ride	1
fluoxetine hydrobromide (fluoxetine hydrobromide)	fluoxetine	1
symbax-olanzapine 6mg / fluoxetine 25mg(olanzapin	fluoxetine; olanzapine	1
fluoxetine hcl(fluoxetine hydrochloride)(capsules)	fluoxetine hcl(fluoxetine hydrochlo-ride)(capsules)	1
fluoxetine oral (fluoxetine (fluoxetine hdyrochlor	fluoxetine	1
fluoxetine hydrochloride (fluoxetine hydrocloride)	fluoxetine; fluoxetine hydrochloride	1
fluoxetine hydrochoride (fluoxetine hydrochloride)	fluoxetine hydrochloride; fluoxetine	1
fluoxetine (fluoxetine) (fluoxetine (r-fluoxetine)	fluoxetine	1
fluoxetine hydrchloride (fluoxetine hydrochloride)	fluoxetine hydrochloride; fluoxetine	1
symbyax-olanzpine 6mg / fluoxetine 50mg (olanza-pin	fluoxetine	1
fluoxetine (fluoxetine hydrochloride) (fluoxetine)	fluoxetine	1
fluoxetine (fluoxetine hydrochloride manufacturer)	fluoxetine hydrochloride; fluoxetine	1
fluoxetine capsules usp 20 mg (alphapharm pty ltd)	fluoxetine	1
fluoxetine hcl (fluoxetine hydrochloride) capsules	fluoxetine hcl (fluoxetine hydrochloride) cap-sules	1
fluoxetine hydrochloride (fluoxetie hydrochloride)	hydrochloride; fluoxetine hydrochloride	1
fluoxetine hydrochloride (floxetine hydrochloride)	hydrochloride; fluoxetine hydrochloride	1
symbyax-olanzapine 12mg / fluoxeint 50mg (olanzapi	fluoxetine; olanzapine	1
floxetine-oral (fluoxetine) (fluoxetine hydrochlor	fluoxetine	1
fluoxetine hcl (fluoxetine hydrochloride) (tablets)	fluoxetine hcl (fluoxetine hydrochloride) (tab-lets)	1
symbyax-olanzapinw 6mg / fluoxetine 25mg (olanzapin	fluoxetine	1
fluoxetine capsules 20mg (fluoxetine) capsule, 20mg	fluoxetine	1
fluoxetine hydrochloride (fluoxetine hydrachloride)	fluoxetine; fluoxetine hydrochloride	1
prozac-oral (fluoxetine) (fluoxetine hydrochlorode)	fluoxetine	1
symbyax-olanzapine 6mg / fluoxetine 50mg (olanazpin	fluoxetine; olanzapine	1
symbyax-olanazapine 6mg/ fluoxetine 25mg (olanzapin	fluoxetine	1
prozac-oral (fluoxetine) (dluoxetine hydrochloride0	fluoxetine	1
prozac-oral (fluoxetine) (fluoxetine hydrochloride)	fluoxetine hydrochloride; fluoxetine	1

prozac-weekly (once a week formulation) (fluoxetine	fluoxetine	1
fontex-oral (fluoxetine) (fluoxetine hydrochloride)	fluoxetine hydrochloride; fluoxetine	1
proazc-oral (fluoxetine) (fluoxetine hydrochloride)	fluoxetine hydrochloride; fluoxetine	1
fluoxetine hydrochlroide (fluoxetine hydrochloride)	fluoxetine hydrochloride; fluoxetine	1
symbyax-olanzapine 6mg / fluoxetine 25mg (olanxapin	fluoxetine; olanzapine	1
symbyax-olanzapine 6mg / fluoxetine 50mg (olanzapin	fluoxetine; olanzapine	1
symbyax-olanzapine 12mg /fluoxetine 25mg(olanzapine	olanzapine; fluoxetine	1
fluoxetine hcl (fluoxetine hydrochloride) (capsules)	fluoxetine hcl (fluoxetine hydrochloride) (capsules)	1
fluoxetine hydrochloride) (fluoxetine hydrochloride)	fluoxetine hydrochloride	1
fluoxetine hydrochloride (fluoxetine hyldrochloride)	fluoxetine; fluoxetine hydrochloride	1
fluoxetine (r-fluoxetine) (fluoxetine (r-fluoxetine)	fluoxetine	1
fluoxetine hydrochloride (fluoxetine hydroichloride)	fluoxetine; fluoxetine hydrochloride	1
symbyak-olanzapine 6mg / fluoxetine 25 mg (olanzapin	fluoxetine; olanzapine	1
fluoxetine (watson laboratories)(fluxoetine) capsule	fluoxetine	1
symbyax-olanzapine 6mg / fluxoetine 25mg (olanzapine	olanzapine; fluoxetine	1
fluoxetiine hydrochloride (fluoxetine hydrochloride)	hydrochloride	1
fluoxetine (watson laboratories)(fluoxetine) capsule	fluoxetine	1
lovan (fluoxetine hydrochloride) 20 mg;unknown;daily	fluoxetine	1
fluoxetine hydrochloride (fluxoxetine hydrochloride)	hydrochloride; fluoxetine hydrochloride	1
fluoxetine hydrochkloride (fluoxetine hydrochloride)	fluoxetine hydrochloride; fluoxetine	1
fluoxetine hydrochloride (fluoxdetine hydrochloride)	hydrochloride; fluoxetine hydrochloride	1
fluoxetine hydrochlioride (fluoxetine hydrochloride)	fluoxetine hydrochloride; fluoxetine	1
ladose (fluoxetine hydrochloride unknown formulation)	fluoxetine hydrochloride	1
symbiax-olanzapine 12 mg / fluoxetine 50 mg (olanzapi	fluoxetine; olanzapine	1
fluoxetine zentiva 20 mg, comprim? dispersible s?cable	fluoxetine zentiva 20 mg, comprim? dispersible s?cable	1
symbax-olanzapine 12 mg/ fluoxetine 25 mg (olanzapine)	olanzapine; fluoxetine	1
fluctin (fluoxetine hydrochloride unknown formulation)	fluoxetine hydrochloride	1
fluoxetine (formulation unknown) (fluoxetine) (generic)	fluoxetine	1

symyax(olanzapine and fluoxetine hydrochloride) capsule	olanzapine;	1
symbax(olanzapine and fluoxetine hydrochloride) capsule	olanzapine;	1
symbyax (olanzapine and fluoxetine hyrochloride) capsule	olanzapine; fluoxetine	1
prozac (fluoxetine hydrochloride) unspecified	fluoxetine	1
prozac (fluoxetine hydrochloride) (10 milligram, tablets)	fluoxetine	1
symbyax (olanzapine and fluoxetine hydrochloride) capsule	olanzapine; fluoxetine;	1
prozac(fluoxetine hydrochloride)(fluoxetine hydrochloride)	fluoxetine hydrochloride; fluoxetine	1
prozac (fluoxetine hydrochloride) fluoxetine hydrochloride)	fluoxetine hydrochloride; fluoxetine	1
prozac (fluoxetine hydrochloride) (fluoxetinehydrochloride)	fluoxetine	1
symbyax (olanzapine + fluoxetine) (olanzapine + fluoxetine)	olanzapine+fluoxetine; fluoxetine	1
prozac (fluoxetine) 20-80mg (varied over time period) lilly	fluoxetine	1
fluoxetine hydrochloride (fluoxetine hydrochloride) capsule	fluoxetine hydrochloride (fluoxetine hydrochloride) capsule	1
prozac (fluoxetine hydrochloride unknown formulation) 20 mg	fluoxetine	1
fluoxetine (watson laboratories) (fluoxetine) capsule, 10mg	fluoxetine	1
metilpredisona / fluoxetine / codeine phosphate / famotidine	famotidine; codeine (as phosphate)	1
prozac (fluoxetine hydrochlordie) (fluoxetine hydrochloride)	prozac (fluoxetine hydrochlordie) (fluoxetine hydrochloride)	1
fluoxetine (fluoxetine hydrochloride) (fluoxetine medoxomil)	fluoxetine	1
fluoxetine hydrochloride (fluoxetine hydrochloride) capsules	fluoxetine hydrochloride (fluoxetine hydrochloride) capsules	1
prozac (fluoxetine hydrochloride) (fluoxetine hydrochloride)	prozac (fluoxetine hydrochloride) (fluoxetine hydrochloride)	1
prozac (fluoxentine hydrochloride) (fluoxetine hydrochloride)	fluoxetine hydrochloride; hydrochloride; fluoxetine	1
fluoxetine hydrochloride (unknown) (fluoxetine hydrochloride)	fluoxetine hydrochloride	1
fluctin(fluoxetine hydrochloride unknown formulation) unknown	fluoxetine hydrochloride	1
fluoxeren - a. menarini industrie farmaceutiche riunite s.r.l.	fluoxetine hydrochloride	1
fluoxeren ? a. menarini industrie farmaceutiche riunite s.r.l.	fluoxetine hydrochloride	1

prozac (fluoxetine hydrochloride) (fluoxetine hydrochloride)	fluoxetine hydrochloride; fluoxetine	1
fluctine (fluoxetine hydrochloride) (fluoxetine hydrochloride)	fluoxetine hydrochloride	1
olanzapine/fluoxetine hydrochloride (fluoxetine hydrochloride)	fluoxetine hydrochloride; olanzapine / fluoxetine hydrochloride	1
fluoxetin (fluoxetine hydrochloride) (fluoxetine hydrochloride)	fluoxetine hydrochloride; fluoxetine	1
fluoxetin (fluoxetin- fluoxetine hydrochloride) (not specified)	fluoxetine hydrochloride; fluoxetine	1
fluxetine (fluoxetine hydrochloride) (fluoxetine hydrochloride)	fluxetine (fluoxetine hydrochloride) (fluoxetine hydrochloride)	1
fluoxetine oral solution usp, 20mg/ 5ml (alpharma) (fluoxetine)	fluoxetine	1
fluoxetine(fluoxetine hydrochloride unknown formulation) unknown	fluoxetine hydrochloride; fluoxetine	1
fluoxetine 10mg capsule, generic for prozac, mfr: pliva inc # 647	fluoxetine hydrochloride	1
symbyax(olanzapine and fluoxetine hydrochloride) capsule, 3/25 mg	olanzapine; fluoxetine;	1
symbyax (olanzapine and fluoxetine hydrochloride) capsule, 6/50mg	olanzapine; fluoxetine;	1
fluoxetine(fluoxetine hydrochloride unknown manufacturer) unknown	fluoxetine hydrochloride; fluoxetine	1
fluoxetine (fluoxetine hydrochloride unknown manufacturer) unknown	fluoxetine hydrochloride; fluoxetine	1
daforin (fluoxetin hydrochloride) (tablet) (fluoxetine hydrochloride)	; hydrochloride; fluoxetine	1
symbyax(olanzapine and fluoxetine hydrochloride unknown manufacturer)	olanzapine; fluoxetine; fluoxetine hydrochloride	1
fluoxetine / generic prozac 10 mg capsules pliva krakow pharmaceutical	fluoxetine hydrochloride	1
prozac (fluoxetine hydrochloride hydrochloride) (fluoxetine hydrochlor	fluoxetine; hydrochloride; fluoxetine hydrochloride	1
prozac (fluoxetine hydrochloride) (tablets) (fluoxetine hydrochloride)	; fluoxetine	1
generic prozac 20mg cannot take cause seizure from dye inthe medicine.	fluoxetine	1
olanzapine/fluoxetine hydrochloride (fluoxetine hydrochloride, , olanz	fluoxetine hydrochloride; olanzapine / fluoxetine hydrochloride	1
fluoxetine oral solution usp, 20mg/5ml (alpharma) (fluoxetine oral sol	fluoxetine	1
fluoxetine oral solution usp, 20mg/5 ml (alpharma) (fluoxetine oral so	fluoxetine	1
fluoxetine hydrochloride (fluoxetine hydrochloride unknown fromulation	fluoxetine hydrochloride	1
symbyax (olanzapine and fluoxetine hydrochloride	olanzapine; fluoxetine; fluoxetine hydrochlo-	1

unknown manufacturer)	ride	
symbyax(olanzapine and fluoxetine hydrochloride unknown formulation) u	olanzapine; fluoxetine; fluoxetine hydrochlo-ride	1
prozac (fluoxetine hydrochloride) capsule advil (mefenamic acid) tablet	ibuprofen; fluoxetine	1
fluoxetine hydrochloride (unknown)(fluoxetine hydrochloride) link, unkunk	fluoxetine hydrochloride	1
fluoxetine hydrochloride (fluoxetine hydrochloride) (fluoxetine hydrochloride)	fluoxetine hydrochloride	1
fluoxetin mylan 20 mg, kapsel, h?rd	fluoxetine hydrochloride	1
fluoxetine (fluoxetine)(fluoxetine)	fluoxetine	1
olanzapine/fluoxetine hydrochloride	olanzapine / fluoxetine hydrochloride	1
fluoxetine hcl - discontinued brand	fluoxetine hcl	1
premarin (fluoxetine hydrochloride)	conjugated estrogens	1
prozac (fluoxetrine hydrochloride)	hydrochloride; fluoxetine	1
fluoxetin (fluoxetin hydrochloride)	hydrochloride; fluoxetine	1
fluoxetine (fluoxetine / 00724401/)	fluoxetine	1
prozac (fluoxetine hydrochlororide)	fluoxetine	1
symbax-olanzapine / fluoxetine (ola	olanzapine / fluoxetine	1
prozac (fluoxetine hydrochloride) c	fluoxetine	1
fluoxetine (fluoxetine hcl 20mg cap)	fluoxetine hydrochloride	1
40mg capsules of fluoxetine hcl 7198	fluoxetine hydrochloride	1
fluoxetin mylan 20 mg kapsler, harde	fluoxetine hydrochloride	1
fluoxetin - fluoxetine hydrochloride	fluoxetine hydrochloride	1
proxetine (fluoxetine hydrochloride)	proxetine (fluoxetine hydrochloride)	1
fluoxeren (fluoxetine hydrochloride)	fluoxetine hydrochloride	1
fluoxetine /n/a/ (fluoxetine) /n/a/)	fluoxetine	1
fluoxetine (furosemide) (furosemide)	furosemide; fluoxetine	1
fluoxetin (fluoxetine hydrochloride0	fluoxetine	1
prozac 20 (fluoxetine hydrochloride)	fluoxetine	1
fluvoxetin (fluoxetine hydrochloride)	fluoxetine hydrochloride	1
fluoxetine 40mg cap ven, 40 mg vensun	fluoxetine hydrochloride	1
fluoxetina [fluoxetine hydrochloride]	fluoxetina [fluoxetine hydrochloride]	1
fluoxetine hydrochloride w/olanzapine	fluoxetine hydrochloride\olanzapine	1
fluoxetine 20mg, tablets (fluoxetine)	fluoxetine	1
fluoxetine-oral (fluoxetine hydrchlor	fluoxetine	1
prozac (fluoxetine hcl) 40mg capsules	fluoxetine	1
fluoxetine (lfuoxetine hydrochloride)	hydrochloride; fluoxetine	1
prozac(fluoxetine hydrochloride) (ta)	fluoxetine	1
fluoxetine (fluoxetuine hydrochloride)	fluoxetine (fluoxetuine hydrochloride)	1

fluoxetine generichealth 20 mg capsule	fluoxetine hydrochloride	1
fluoxetinne (fluoxetine hydrochloride)	fluoxetine hydrochloride	1
fluoxetine (fluoxetine) capsule, 10 mg	fluoxetine	1
fluoxetine (fluoxetine) capsule, 40 mg	fluoxetine	1
fluoxetine (fluoxetine hydrocfhloride)	fluoxetine (fluoxetine hydrocfhloride)	1
fluoxetine. mfr: lilly eli and company	fluoxetine. mfr: lilly eli and company	1
fluoxetine (nghx) (fluoxetine) unknown	fluoxetine	1
fluoxetine (fluoxetine) (10 milligram)	fluoxetine	1
fluoxetine (fluoxetine) (40 milligram)	fluoxetine	1
fluoxetine hcl (pmdd) oral capsule 10mg	fluoxetine hcl (pmdd) oral capsule 10mg	1
fluoxetine (fluoxetine) capsule ongoing	fluoxetine	1
fluoxetine (fluoxetine), capsule, 20 mg	fluoxetine	1
12mg/50mg olanzapine/fluoxetine capsule	fluoxetine	1
symbyax olanzapine 6 mg/fluoxeitne 25mg	fluoxetine; olanzapine	1
fluoxetine hydrochloride and olanzapine	fluoxetine hydrochloride; olanzapine	1
fluoxetine w/olanzapine (not specified)	fluoxetine w / olanzapine	1
prozac (fluoxetine hydrochlloride)	fluoxetine	1
prozac (fluoxeitine hydrochloride)	hydrochloride; fluoxetine	1
fluoxetine hcl oral solution akorn, inc.	fluoxetine hydrochloride	1
substituted for prozac pulvule pliva-647	fluoxetine hydrochloride	1
nor-fluoxetine (nor-fluoxetine), unknown	fluoxetine	1
prozac (fluoxetine)(tablets)(fluoxetine)	fluoxetine	1
12mg/50 mg olanzapine/fluoxetine capsule	fluoxetine	1
ladose(fluoxetine hydrochloride) capsule	ladose(fluoxetine hydrochloride) capsule	1
fluoxetine capsules (fluoxetine) capsule	fluoxetine	1
prozac (fluoxetine hydrochloride) liquid	fluoxetine	1
fluctine /sch/(fluoxetine hydrochloride)	fluoxetine hydrochloride	1
prozac weekly (fluoxetine ydrochloriide)	fluoxetine	1
fontex (fluoxetine hydrochloride) tablet	fontex (fluoxetine hydrochloride) tablet	1
symbyax olanzapine 6mg / fluoxetine 25mg	fluoxetine; olanzapine	1
prozac (fluoxetine hydrochloride)tablets	fluoxetine	1
fluoxetin ratiopharm 10 mg l?slig tablett	fluoxetine hydrochloride	1
fluoxetin ratiopharm 10 mg loslig tablett	fluoxetine hydrochloride	1
fluoxetine hcl 20mg rx number 706066-7009	fluoxetine hydrochloride	1
prozac (fluoxetine hydrochloride) ongoing	fluoxetine	1
prozac durapac (fluoxetine hydrochloride)	prozac durapac (fluoxetine hydrochloride)	1
prozac(fluoxetine hydrochloride)(unknown)	fluoxetine	1
prozac (fluoxetine) (tablets)(fluoxetine)	fluoxetine	1
apo-fluoxetine (fluoxetine hydrochloride)	apo-fluoxetine	1

ladose (fluoxetine hydrochloride) capsule	ladose (fluoxetine hydrochloride) capsule	1
fluctin (fluoxetine hydrochloride) tablet	fluctin (fluoxetine hydrochloride) tablet	1
fluoxetin mepha(fluoxetine hydrochloride)	fluoxetine hydrochloride; fluoxetine	1
fluoxetine hydrochloride (generic prozac)	fluoxetine; fluoxetine hydrochloride	1
prozac (tablets) fluoxetine hydrochloride	; fluoxetine	1
prozac (unknown) fluoxetine hydrochloride	fluoxetine hydrochloride; fluoxetine	1
allegra (fluoxetine hydrochloride)	fexofenadine	1
fluoxetine 10 mg generic for prozac ep 360	fluoxetine hydrochloride	1
affex (fluoxetine) (capsules) (fluoxetine)	fluoxetine	1
fluctine(fluoxetine hydrochloride) capsule	fluctine(fluoxetine hydrochloride) capsule	1
fluoxetin mepha (fluoxetine hydrochloride)	fluoxetine hydrochloride; fluoxetine	1
cloridrato de fluoxetina (ngx)(fluoxetine)	fluoxetine	1
fluoxetine (ngx) (fluoxetine) tablet, 20mg	fluoxetine	1
symbyax olanzapine 12 mg/ fluoxetine 25 mg	fluoxetine; olanzapine	1
fluoxetine hydrochloride-0ral (fluoxetine)	fluoxetine; fluoxetine hydrochloride	1
prozac (fluoxetine hydorchloride) (unknown)	fluoxetine	1
genrx fluoxetine (fluoxetine hydrochloride)	fluoxetine	1
prozac (generic) (fluoxetine hydrochloride)	fluoxetine hydrochloride; fluoxetine	1
fluctine (fluoxetine hydrochloride) capsule	fluctine (fluoxetine hydrochloride) capsule	1
fluoxetine capsules, 20 mg (siegfried ltd.)	fluoxetine	1
cloridrato de fluoxetina (ngx) (fluoxetine)	fluoxetine	1
fluoxetine, letrozole, losartan hctz, zofran	fluoxetine, letrozole, losartan hctz, zofran	1
fluoxetine hcl 20mg caps, ndc# 50111-0648-03	fluoxetine hydrochloride	1
fluoxetine hcl 40 mg caps generic for prozac	fluoxetine hydrochloride	1
fluoxetine (fluoxetine hydrochloride) liquid	fluoxetine hydrochloride	1
fluoxetine hcl 40mg capsule, 40 mg par pharm	fluoxetine hydrochloride	1
fluoxetine 40 mg, 40 mg teva pharmaceuticals	fluoxetine hydrochloride	1
zoplicone wellbutrin fluoxetine valacyclovir	valaciclovir; fluoxetine; bupropion	1
fluoxetine (fluoxetine hydrochloride), 10 mg	fluoxetine	1
fluoxetine hydrochloride (generic of prozac)	fluoxetine; fluoxetine hydrochloride	1
fluoxetine (fluoxetine) (fluoxetine) (20 mg)	fluoxetine	1
fluctine (fluoxetine hydrochloride) capsule	fluctine (fluoxetine hydrochloride) capsule	1
fluoxetine (fluoxetine hydrochloride) (20 mg)	fluoxetine (fluoxetine hydrochloride) (20 mg)	1
prozac (unspecified) fluoxetine hydrochloride	fluoxetine hydrochloride; fluoxetine	1
fluoxetina 20 mg 28 c?psulas	fluoxetine hydrochloride	1
fluoxetine 10mg 3 caps daily	fluoxetine hydrochloride	1
fluoxetine hcl (prozac oral)	fluoxetine; fluoxetine hcl	1
fluoxetine /n/a/ (fluoxetine	fluoxetine	1
zyprexa /symbyax /olanzapine	olanzapine; fluoxetine	1

fluoxetine usp - ipi unknown	fluoxetine	1
fluoxetine unknonw generic -	fluoxetine	1
fluoxetine (ngx) (fluxetine)	fluoxetine	1
fluoxetine (fluoxetine) (ka)	fluoxetine	1
fluoxetine (fluoxetine, , 0)	fluoxetine	1
fluoxetine 40 mg rx #6172091	fluoxetine	1
generic prozac 40 mg per day	fluoxetine	1
fluoxetine 60mg hard capsules	fluoxetine hydrochloride	1
fluoxetine hydrochloride 40mg	fluoxetine hydrochloride	1
fluoxetine teva 20 mg, g?lule	fluoxetine hydrochloride	1
fluoxetine hydrochloride 10mg	fluoxetine hydrochloride	1
fluoxetine hcl 40 mg cap auro	fluoxetine hydrochloride	1
fluoxetine hcl 40 mg cap teva	fluoxetine hydrochloride	1
fluoxetine (fluoxetine), 40mg	fluoxetine	1
prozac (fluoxetine) (tablets)	fluoxetine	1
fluoxetine (fluoxetine /n/a/)	fluoxetine	1
olaznapine/fluoxetine capsule	fluoxetine	1
fluoxetine usp capsules - ipi	fluoxetine	1
fluoxetine 40 mg rx # 6150571	fluoxetine	1
restoril 15mg, fluoxetine 20mg	restoril 15mg, fluoxetine 20mg	1
fluoxetine hydrochloride 40 mg	fluoxetine hydrochloride	1
fluoxetine tablets, usp 60 mg.	fluoxetine hydrochloride	1
calcium, celecoxib, fluoxetine	calcium, celecoxib, fluoxetine	1
flute/fluoxetine hydrochloride	flute/fluoxetine hydrochloride	1
fluoxitine 20 mg. tablet mylan	fluoxetine hydrochloride	1
fluoxetine hydrochloride (ngx)	fluoxetine hydrochloride	1
prozac (20 milligram, tablets)	fluoxetine	1
fluoxetine family (fluoxetine)	fluoxetine	1
fluoxetine gelule (fluoxetine)	fluoxetine	1
olanzapine /fluoxetine capsule	fluoxetine	1
rize [fluoxetine hydrochloride]	rize [fluoxetine hydrochloride]	1
fluoxetine (generic for prozac)	fluoxetine (generic for prozac)	1
azur [fluoxetine hydrochloride]	fluoxetine hydrochloride	1
modafinil, ursodiol, fluoxetine	modafinil, ursodiol, fluoxetine	1
fluoxetin hexal 10 mg tabletten	fluoxetine	1
fluxin/fluoxetine hydrochloride	fluxin/fluoxetine hydrochloride	1
generic prozac 40mg - northstar	fluoxetine	1
fluoxetine (fluoxetine) (60 mg)	fluoxetine	1
fluoxetine ranbaxy 20 mg gelule	fluoxetine	1

fluoxetine hydrochloride (con.)	fluoxetine hydrochloride	1
fluoxetine manufacturer unknown	fluoxetine	1
fluox?tine (chlorhydrate de)	fluoxetine hydrochloride	1
fluoxetine solution 20mg/5ml	fluoxetine solution 20mg/5ml	1
fluoxetine mylan 20 mg, capsules	fluoxetine hydrochloride	1
fluoxetine(fluoxetine)(capsules)	fluoxetine	1
mutan (fluoxetine hydrochloride)	fluoxetine hydrochloride	1
prozac(fluoxetine hydrochloride)	prozac(fluoxetine hydrochloride)	1
symeyax (ulanzapine, fluoxetine)	fluoxetine	1
fluoxetine 10 mg unknown-generic	fluoxetine	1
fluoxetine polyfarma(fluoxetine)	fluoxetine	1
fontex(fluoxetine hydrochloride)	fluoxetine hydrochloride	1
affex (fluoxetine hydrochloride)	affex (fluoxetine hydrochloride)	1
prozac (fluoxetine hydorchoride)	fluoxetine	1
prozac (fluoxetine hyrochloride)	fluoxetine	1
prozac (fluoxetine hydochloride)	fluoxetine	1
prozac (luoxetine hydrochloride)	hydrochloride; fluoxetine	1
fluoxetin (fluoxetine hydrochlor	fluoxetine	1
fluoxetine (fluoxetine) capsules	fluoxetine	1
fluoxetine (fluoxetine) tablets	fluoxetine	1
prozac (fluoxeine hydrochloride)	hydrochloride; fluoxetine	1
fluoxetin beta 20, tabletten	fluoxetine hydrochloride	1
prizma [fluoxetine hydrochloride]	prizma [fluoxetine hydrochloride]	1
flocet [fluoxetine hydrochloride]	flocet [fluoxetine hydrochloride]	1
prozin [fluoxetine hydrochloride]	fluoxetine hydrochloride	1
fluoxetine hcl - 10mg - pliva 647	fluoxetine hydrochloride	1
motivest (fluoxetine) 20mg, 20 mg	fluoxetine hydrochloride	1
extine [fluoxetine hydrochloride]	extine [fluoxetine hydrochloride]	1
fluzac (fluoxetine hydrochloride)	fluoxetine hydrochloride	1
fluoxetine hydrochloride (watson)	fluoxetine hydrochloride	1
fluoxetine (fluoxetine) (tablets)	fluoxetine	1
ladose (fluoxetine hydrochloride)	ladose (fluoxetine hydrochloride)	1
fluoxetine(fluoxetine(fluoxetine)	fluoxetine	1
prozac (fluoxetine) (fluoxetine)	fluoxetine	1
prozak (fluoxetine hydrochloride)	fluoxetine hydrochloride	1
prozac (floxetine hydrochloriede)	fluoxetine	1
prozac (fluoextine hydrochloride)	hydrochloride; fluoxetine	1
prozac (fluoxetine hdyrochloride)	fluoxetine	1
prozac (fluoxeitne hydrochloride)	hydrochloride; fluoxetine	1

proazc (fluoxetine hydrochloride)	fluoxetine hydrochloride	1
fluoxetine polyfarma (fluoxetine)	fluoxetine	1
prozac (fluoxdtine hydrochloride)	hydrochloride; fluoxetine	1
prozac (fluoxetine nydrochloride)	fluoxetine	1
fluoxetine (fluoxetine hydrochlor	fluoxetine	1
prozac (fluoxentine hydrocloride)	fluoxetine	1
prozac (fluoxerine hydrochloride)	hydrochloride; fluoxetine	1
prazac (fluoxetine hdyrochloride)	fluoxetine	1
fluoxetine hydrochloride capsules	fluoxetine hydrochloride capsules	1
prozac (fluoxetine fydrochloride)	fluoxetine	1
flagyl (fluoxetine hydrochloride)	metronidazole	1
prozac (fluocetine hydrochloride)	hydrochloride; fluoxetine	1
prozac (fluxoetine hydrochloride)	hydrochloride; fluoxetine	1
prozac (flooxetine hydrochloride)	hydrochloride; fluoxetine	1
symbyax (fluoxetine, olanazapine)	fluoxetine	1
prozca (fluoxetine hydrochloride)	fluoxetine hydrochloride	1
prozaxc(fluoxetine hydrochloride)	fluoxetine hydrochloride	1
fluoxetine 20 mg (generic prozac)	fluoxetine	1
prozac (fluoxetine hydrohcloride)	fluoxetine	1
prozac (fluoxeyine hydrochloride)	hydrochloride; fluoxetine	1
prozaca(fluoxetine hydrochloride)	fluoxetine hydrochloride	1
prozac (fluoxetine hydrochloried)	fluoxetine	1
fluoxetine hcl 30 mg capsule	fluoxetine hydrochloride	1
fluoxetine hcl 40mg capsule, 40 mg	fluoxetine hydrochloride	1
fodiss 20 mg disperzibilne tablete	fluoxetine hydrochloride	1
zedprex (fluoxetine hydrochloride)	zedprex (fluoxetine hydrochloride)	1
fluoxetine (prozac) 20 mg oral cap	fluoxetine (prozac) 20 mg oral cap	1
lovan (fluoxetine hydrochloride) c	fluoxetine hydrochloride	1
daforin (fluoxetine hydrochloride)	fluoxetine hydrochloride	1
prozac (flucoxetine hydrochloride)	hydrochloride; fluoxetine	1
fluoxetine (fluoxetine) (unknown)?	fluoxetine	1
prozac (fluoxetine hydrochlolride)	fluoxetine	1
flutine (fluoxetine hydrochloride)	fluoxetine hydrochloride	1
lovan (fluoxetine hydrochloride,)	fluoxetine hydrochloride	1
prozac (fluoxetine hydrochloridde)	fluoxetine	1
fluoxetine/n/a/ (fluoxetine /n/a/)	fluoxetine	1
prozac (fludoxetine hydrochloride)	hydrochloride; fluoxetine	1
fluctin (fluoxetine hydrochloride)	fluoxetine hydrochloride	1
prozac (fluxoxetine hydrochloride)	hydrochloride; fluoxetine	1

fluoxetine 10 mg/20 mg mallincrodt	fluoxetine	1
fluoxetine (fluoxetine/ 00724401/)	fluoxetine	1
fluctine(fluoxetine hydrochloride)	fluoxetine hydrochloride	1
fluoxetine 40 mg po bid	fluoxetine 40 mg po bid	1
fluoxetine 20mg capsule	fluoxetine hydrochloride	1
fluoxetine 20 mg po qid	fluoxetine 20 mg po qid	1
fluoxetine hcl 10mg cap	fluoxetine hydrochloride	1
fluoxetina - get s.r.l.	fluoxetine hydrochloride	1
fluoxetine, 60mg sandoz	fluoxetine hydrochloride	1
generic fluoxetine 20mg	fluoxetine hydrochloride	1
chlorhydrate fluoxetine	chlorhydrate fluoxetine	1
fluoxetine 40mg po qday	fluoxetine 40mg po qday	1
fluoxitine 40mg capsule	fluoxetine hydrochloride	1
olanzapine/ wfluoxetine	fluoxetine hydrochloride\olanzapine	1
fluoxetine (non-abbvie)	fluoxetine (non-abbvie)	1
fluoxetine hcl 40mg cap	fluoxetine hydrochloride	1
fluoxetine hydrochlorot	fluoxetine hydrochlorot	1
10 unknown (fluoxetine)	fluoxetine hydrochloride	1
fluoxetine (fluoextine)	fluoxetine	1
fluoxetine + olanzapine	fluoxetine hydrochloride\olanzapine	1
olanzapine w/fluoxetine	fluoxetine hydrochloride\olanzapine	1
olanzapine + fluoxetine	olanzapine+fluoxetine	1
fluoxetine hydrochoride	fluoxetine	1
fluoxetine hydrchloride	fluoxetine	1
fluoxetine (fluexitine)	fluoxetine	1
flioxetine (fluoxetine)	fluoxetine	1
fluocetine (fluoxetine)	fluoxetine	1
paroxetine (fluoxetine)	paroxetine	1
flouxetine (fluoxetine)	fluoxetine	1
fluoxetine hyrochloride	fluoxetine hyrochloride	1
fluoxetine hydrohloride	fluoxetine	1
fluoxetine (fluocetine)	fluoxetine	1
fluoxetine (fluoxteine)	fluoxetine	1
fluxoetine (fluoxetine)	fluoxetine	1
fluoxetine (flyoxetine)	fluoxetine	1
fluoxitine (fluoxetine)	fluoxetine	1
fluoxetine hcl 40 mg cap	fluoxetine hydrochloride	1
fluoxetine (prozac) 20mg	fluoxetine (prozac) 20mg	1
fluoxetine 40mg po daily	fluoxetine 40mg po daily	1

teva fluoxetine capsules	fluoxetine hydrochloride	1
fluoxetine 20 mg cap nor	norfluoxetine	1
fluoxetine/norfluoxetine	fluoxetine/norfluoxetine	1
fiuoxetine 40mg, 40mg mg	fluoxetine hydrochloride	1
fluoxetine/prozac (lsid)	fluoxetine/prozac (lsid)	1
fluoxetine hydorchloride	fluoxetine	1
fluoxetine hcl 20mg tabs	fluoxetine hcl 20mg tabs	1
fluoxetine usp, 20mg/5ml	fluoxetine	1
fluoxetine (fluoxetine)	fluoxetine	1
fluoxfetine (fluoxetine)	fluoxetine	1
fluoxetine solution, ups	fluoxetine	1
fluoxetine hcl 20 mg unk	fluoxetine hcl 20 mg unk	1
fluoxetine (flouoxetine)	fluoxetine	1
fluoxetine 10 mg tablets	fluoxetine	1
fluoxetine (fluxoxetine)	fluoxetine	1
fluoxetine 20mg daily po	fluoxetine	1
fluoxetine hcl 20mg sidm	fluoxetine hcl 20mg sidm	1
fluoxetinie (fluoxetine)	fluoxetine	1
fluoxetine hydrochlroide	fluoxetine	1
hydrochloride fluoxetine	fluoxetine; hydrochloride	1
fluoxetin nm(fluoxetine)	fluoxetine	1
fluox basan (fluoxetine)	fluoxetine;	1
pms-fluoxetine - cap 20mg	fluoxetine hydrochloride	1
fluoxetin 40 - 1 a pharma	fluoxetine hydrochloride	1
fluoxetin 20 - 1 a pharma	fluoxetine hydrochloride	1
fluoxetine 40 mg po daily	fluoxetine 40 mg po daily	1
fluoxetine karica/viorele	fluoxetine karica/viorele	1
proair hfa fluoxetine hcl	proair hfa fluoxetine hcl	1
fluoxetine 20 mg po daily	fluoxetine 20 mg po daily	1
fluoxetine and olanzapine	fluoxetine hydrochloride\olanzapine	1
fluoxetine capsules 20 mg	fluoxetine capsules 20 mg	1
niche generics fluoxetine	niche generics fluoxetine	1
fluoxetine (non-specific)	fluoxetine	1
fluoxetine 40mg par pharm	fluoxetine hydrochloride	1
fluoxetine prozac sarafem	fluoxetine	1
pms-fluoxetine - cap 10mg	fluoxetine hydrochloride	1
fluoxetine hyudrochloride	fluoxetine	1
fluoxetine (barr) (20 mg)	fluoxetine	1
fluoxetine hyidrochloride	fluoxetine	1

fluctine (fluoxetine hcl)	fluoxetine hcl	1
fluoxetine 40 mg bid parr	fluoxetine	1
fluoxetine (floxetine)	fluoxetine	1
fluoxetine 20mg once daily	fluoxetine 20mg once daily	1
fluoxetine, prozac generic	fluoxetine hydrochloride	1
fluoxetine, 20mg eli lilly	fluoxetine hydrochloride	1
fluoxetine (fluoxetine)???	fluoxetine (fluoxetine)???	1
fluoxetine hcl 10mg prozac	fluoxetine; fluoxetine hcl	1
fluoxetine capsules 76-990	fluoxetine	1
fluoxetine hcl-prozac oral	fluoxetine; fluoxetine hcl	1
fluoxetine oral (alpharma)	fluoxetine	1
luvox (fluoxetine maleate)	maleate; fluoxetine; fluvoxamine	1
fluoxetine hcl 20 mg tevau	fluoxetine hcl 20 mg tevau	1
fluoxetine/na/(fluoxetine)	fluoxetine; fluoxetine / na	1
fuoxetine (fluoxetine hcl)	fluoxetine hcl	1
fluoxetine (generic prozac)	fluoxetine (generic prozac)	1
fluoxetine hcl 10mg or caps	fluoxetine hydrochloride	1
pliva 647 fluoxetine, 10 mg	fluoxetine hydrochloride	1
fluoxetine 20 mg cap, 20 mg	fluoxetine hydrochloride	1
gerozac 20 mg capsules hard	fluoxetine hydrochloride	1
fluoxetine hcl 20 mg (pmdd)	fluoxetine hcl 20 mg (pmdd)	1
generic prozac (fluoxetine)	fluoxetine hydrochloride	1
fluoxetine (no pref. namre)	fluoxetine	1
fluoxetine hcl (fluoxetine)	fluoxetine hcl	1
fluctine (fluoxetine) 20 mg	fluoxetine	1
fluoxetine /na/(fluoxetine)	fluoxetine; fluoxetine / na	1
fluoxetine (fluoxetine, ,0)	fluoxetine	1
fluoxzetine-prozac	fluoxetine	1
fluoxetine cap 10mg	fluoxetine cap 10mg	1
fluoxetine tab 20mg	fluoxetine tab 20mg	1
fluoxetine tab 40mg	fluoxetine tab 40mg	1
fluoxetine (procac)	fluoxetine (procac)	1
fluoxetine cap 40mg	fluoxetine cap 40mg	1
ferosul, fluoxetine	ferosul, fluoxetine	1
fluoxetine 20mg cap	fluoxetine 20mg cap	1
fuoxetine hcl 20 mg	fluoxetine hydrochloride	1
generic prozac 40mg	fluoxetine hydrochloride	1
fluoxetine 40mg/day	fluoxetine 40mg/day	1
prosac (fluoxetine)	prosac (fluoxetine)	1

fluoxetine/generics	fluoxetine hydrochloride	1
tramodol fluoxetine	tramodol fluoxetine	1
fluoxetine biogaran	fluoxetine	1
prozac (fluoxatine)	fluoxetine	1
prozac-generic form	fluoxetine	1
fluoxetine(unknown)	fluoxetine	1
floxet (fluoxetine)	fluoxetine	1
fluoxetine hcl caps	fluoxetine	1
sarafem (fluoxetne)	fluoxetine	1
adofen (fluoxetine)	fluoxetine	1
prozac -fluoxetine0	fluoxetine	1
bioxin (fluoxetine)	fluoxetine	1
sarafem(fluoxetine)	fluoxetine	1
fluoxetine 20 mg bid	fluoxetine 20 mg bid	1
fluoxetine tab 10 mg	fluoxetine tab 10 mg	1
fluoxetine cap 10 mg	fluoxetine cap 10 mg	1
fluoxetine 20 mg cap	fluoxetine 20 mg cap	1
fluoxetine 20mg caps	fluoxetine 20mg caps	1
fluoxetine 20mg/5 ml	fluoxetine 20mg/5 ml	1
fluoxetine 10 mg cap	fluoxetine 10 mg cap	1
fluoxetine hci 10 mg	fluoxetine hydrochloride	1
fluoxetine 20mg teva	fluoxetine	1
fluoxetine 10 mg 1xd	fluoxetine 10 mg 1xd	1
fluoxetin ratiopharm	fluoxetine	1
fluoxetine oxycodone	fluoxetine oxycodone	1
fluoxetine sandoz 20	fluoxetine	1
fluoxetine (sarafem)	fluoxetine	1
prozac 20mg tablet	fluoxetine	1
fluoxetine tablets	fluoxetine tablets	1
fluoxetine,olanzapine	fluoxetine hydrochloride\olanzapine	1
fluoxetine 20mg, oral	fluoxetine 20mg, oral	1
florexal [fluoxetine]	florexal [fluoxetine]	1
fluoxetine 40mg ranba	fluoxetine hydrochloride	1
compounded fluoxetine	compounded fluoxetine	1
fluoxetine teva 20 mg	fluoxetine hydrochloride	1
fluoxetine;olanzapine	fluoxetine;olanzapine	1
prozac (fluoxetine)	prozac (fluoxetine)	1
fluoxetine (capsules)	fluoxetine	1
fluoxetine hcl prozac	fluoxetine; fluoxetine hcl	1

prozac (80 milligram)	fluoxetine	1
fluoxetine --/--/2007	fluoxetine --/--/2007	1
olanzepine/fluoxetine	fluoxetine	1
fluoxetine 40 mg teva	fluoxetine	1
fluoxetine gabapentin	gabapentin; fluoxetine	1
bioxetin (fluoxetine)	fluoxetine	1
prozac / 00724401/	fluoxetine	1
cozac (fluoxetine)	fluoxetine	1
fluoxetine capsule	fluoxetine	1
fluoxetine 20 mg daily	fluoxetine 20 mg daily	1
fluoxetine/dicyclomine	fluoxetine/dicyclomine	1
venlafaxine/fluoxetine	venlafaxine/fluoxetine	1
fluoxetine, ropinerole	fluoxetine, ropinerole	1
fluoxetine/ olanzapine	fluoxetine hydrochloride\olanzapine	1
fluoxetine arrow 20 mg	fluoxetine hydrochloride	1
fluoxetine, olanzapine	fluoxetine hydrochloride\olanzapine	1
fluoxecap [fluoxetine]	fluoxetine hydrochloride	1
prozep chemidex pharma	fluoxetine hydrochloride	1
fluoxetine 20mg sandoz	fluoxetine hydrochloride	1
fluoxetine fluoxetine)	fluoxetine	1
fluoxetine hcl capsule	fluoxetine	1
fluoxetine (fuoxetine)	fluoxetine	1
prozac generic capsule	fluoxetine	1
fluoxetine 10mg po qam	fluoxetine	1
fluoxetine 20 mg lilly	fluoxetine	1
fluoxetine (fluoxtine)	fluoxetine	1
lovan (fluoxetine hcl)	fluoxetine hcl	1
fluoxetine and placebo	fluoxetine	1
pms fluoxetine	fluoxetine hydrochloride	1
apo fluoxetine	fluoxetine hydrochloride	1
fluoxetin base	fluoxetine	1
anzack tablets	fluoxetine hydrochloride	1
fluoxetine sol	fluoxetine sol	1
prozac pulvule	fluoxetine	1
nor-fluoxetine	fluoxetine	1
fluoxetine/n/a	fluoxetine	1
fluoxetine hc1	fluoxetine	1
fluoxetine-teva	fluoxetine hydrochloride	1
fluoxetine 25mg	fluoxetine 25mg	1

fluoxeren 20 mg	fluoxetine hydrochloride	1
fluoxetine h cl	cl; fluoxetine	1
oral fluoxetine	fluoxetine	1
fluoxetine 15 mg	fluoxetine 15 mg	1
portal (croatia)	fluoxetine hydrochloride	1
fluoxetine, oral	fluoxetine, oral	1
fluoxetine 30 mg	fluoxetine 30 mg	1
arrow-fluoxetine	fluoxetine hydrochloride	1
fluoxetine merck	fluoxetine merck	1
fluoxetine cinfa	fluoxetine cinfa	1
fluoxetine, 20mg	fluoxetine hydrochloride	1
fluoxetine hexal	fluoxetine hexal	1
mylan?fluoxetine	fluoxetine hydrochloride	1
fluoxetine 25 mg	fluoxetine 25 mg	1
prozac 20 mg qam	fluoxetine	1
lovan/fluoxetine	lovan/fluoxetine	1
fluoxetine g gam	fluoxetine g gam	1
fluoxetine almus	fluoxetine	1
fluoxetine 20 mg	fluoxetine	1
lilly-fluoxetine	fluoxetine	1
prozac clonidine	clonidine; fluoxetine	1
fluoxetin (con.)	fluoxetine	1
prozac/risperdal	risperidone; fluoxetine	1
fluoxetina nodepe	fluoxetine hydrochloride	1
fluoxetine hcl 40	fluoxetine hcl 40	1
fluoxetine hcl 20	fluoxetine hcl 20	1
fluoxetine tablet	fluoxetine tablet	1
fluoxetine accord	fluoxetine	1
fluoxetine 20mg ?	fluoxetine	1
fluoxetina sandoz	fluoxetine hydrochloride	1
epipen fluoxetine	fluoxetine; epinephrine	1
fluoxetine (pill)	fluoxetine	1
generic of prozac	fluoxetine	1
nexium fluoxetine	fluoxetine; esomeprazole	1
prozac iron pills	fluoxetine	1
symbyax (tablets)	fluoxetine	1
zyprexa / symbyax	fluoxetine; olanzapine	1
prozac / 00724401	fluoxetine	1
fluoxetine 60mg hs	fluoxetine 60mg hs	1

fluoxetine hyorhax	fluoxetine hyorhax	1
fluoxetine (paxil)	fluoxetine (paxil)	1
arrow (fluoxetine)	fluoxetine hydrochloride	1
fluoxetine zentiva	fluoxetine zentiva	1
prozac prozac 40mg	fluoxetine	1
flutine 20	fluoxetine hydrochloride	1
gen prozac	fluoxetine	1
prozac 200	fluoxetine	1
prozac cap	fluoxetine	1
fluoxetineq	fluoxetine hydrochloride	1
fluoxetine,	fluoxetine,	1
flucoxetine	fluoxetine hydrochloride	1
prozac 40mg	fluoxetine	1
fluoxetine?	fluoxetine	1
(fluoxetine)	(fluoxetine)	1
prozac(con.)	fluoxetine	1
prozac orals	fluoxetine	1
fluoxetine an	fluoxetine an	1
co fluoxetine	co fluoxetine	1
neo fluoxetin	fluoxetine;	1
prozac?	fluoxetine	1
depricap	fluoxetine hydrochloride	1
fluoxetin 40 mg	fluoxetine	1
fluoxetine 20 mg po once daily	fluoxetine	1
prozac 20mg tablets	fluoxetine	1
fluoxetine br	fluoxetine	1
fluoxetin 30mg	fluoxetine hydrochloride	1
pms?fluoxetine ? cap 20mg	fluoxetine hydrochloride	1
flouxetine sandoz	fluoxetine hydrochloride	1
fluoxetine hcl caps 20 mg	fluoxetine hydrochloride	1
fluoxetine (fluoxetine hcl 40mg cap)	fluoxetine hydrochloride	1
fluoxetine hcl 20 mg capsule	fluoxetine hydrochloride	1
fluoxetina-ratiopharm 20 mg compresse solubili	fluoxetine hydrochloride	1
prozac 40mg daily	fluoxetine	1
alprazolam;fluoxetine hydrochloride	alprazolam; fluoxetine hydrochloride	1
fluoxetine hcl oral capsule 20 mg		1
prozac 40	fluoxetine	1
fluoxetine actavis 20 mg, comprime dispersible secable	fluoxetine hydrochloride	1

fluoxetine disper mylan 20 mg, dispergeerbare tab-letten	fluoxetine hydrochloride	1
fluoxetine hcl 60mg, oral		1
olanzapine and fluoxetine hcl	olanzapine; fluoxetine hcl	1
fluoxetine 20 mg, oral	fluoxetine	1
fluoxetine 80 mg daily	fluoxetine	1
fluoxetine 5 mg	fluoxetine	1
fluoxetin 10mg	fluoxetine hydrochloride	1
fluoxetine (non?abbvie)	fluoxetine	1
fluoxetine ratiopharm	fluoxetine	1
fluoxetine pliva	fluoxetine hydrochloride	1
pliva teva (fluoxetine)	fluoxetine hydrochloride	1
fluoxetine dispertablet 20mg / prozac disp tablet 20mg	fluoxetine	1
fluoxetine sun	fluoxetine hydrochloride	1
fluoxetin hexal 40 mg tabletten	fluoxetine hydrochloride	1
fluoxetine 80 mg po qam	fluoxetine	1
fluoxetine tablets, 10 mg	fluoxetine hydrochloride	1
fluoxetine oral solution usp 20 mg/5 ml	fluoxetine hydrochloride	1
singulair, dulera, spiriva, prozac	montelukast; formoterol; tiotropium; fluoxetine	1
chemidex pharma fluoxetine	fluoxetine	1
dulera, spiriva, prozac	formoterol; tiotropium; fluoxetine	1
fluoxetine 20 mg/5 ml solution	fluoxetine hydrochloride	1
fontex 20mg	fluoxetine hydrochloride	1
fluoxetine buspirone	buspirone; fluoxetine	1
fluoxetine arrow 20 mg, g?lule	fluoxetine hydrochloride	1
ashlyna, pantoprazole, trazodone, fluoxetine, atorvastatin	ashlyna; pantoprazole; trazodone; fluoxetine; atorvastatin	1
fluoxetin teva 20 mg kapsel, hard	fluoxetine hydrochloride	1
meloxicam/fluoxetine	meloxicam / fluoxetine	1
fluoxetine 60 mg tablet	fluoxetine	1
fluoxetine, atovasquone, prednisone, metoclopram, diltiazem	fluoxetine; prednisone; diltiazem	1
fluo [fluoxetine]	fluoxetine hydrochloride	1
fluozetin (fluoxetine)	fluoxetine hydrochloride	1
fidia generics (fluoxetine)	fluoxetine hydrochloride	1
fluoxetina teva 20 mg capsulas	fluoxetine hydrochloride	1
prozac 60 mg	fluoxetine	1
prozac 20mgproair relafen ds 1000mg	nabumetone; fluoxetine	1
fluoxetine hcl,	fluoxetine hcl	1

fluoxetin ratiopharm 20 mg loslig tablett	fluoxetine hydrochloride	1
ephedra, fluoxetine	ephedra; fluoxetine	1
fluoxetine propranolol	propranolol; fluoxetine	1
fluoxetine (home medication)	fluoxetine	1
fluoxetine hcl 10mg capsule	fluoxetine hydrochloride	1
fluoxetine cf	fluoxetine hydrochloride	1
fluoxetine, benztropine, hydroxyzine pamoate	fluoxetine; benzatropine; hydroxyzine pamoate	1
fluoxetin stada 20 mg dispergerbar tablett	fluoxetine	1
fluoxetine/amino acids nos	amino acids	1
olanzapine-fluoxetine	fluoxetine; olanzapine	1
fluoxetine 40 mg po	fluoxetine	1
fluoxetine (prozac) 40 mg capsule	fluoxetine	1
?prozac	fluoxetine	1
fluoxetine 20mg hs	fluoxetine	1
fluval [fluoxetine]	fluoxetine	1
prozac cap 20mg	fluoxetine	1
proctin [fluoxetine hydrochloride]	fluoxetine hydrochloride	1
fluoxetine, 40mg daily	fluoxetine	1
flomax fluoxetine hci	fluoxetine; tamsulosin	1
fluoxetine hcl er	fluoxetine hcl	1
fluoxetine hcl (10 mg)		1
fluoxetine capsule 20mg / brand name not specified-fluoxetine capsul.	fluoxetine	1
symbyax cap	fluoxetine	1
fluoxetine 10mg in the morning	fluoxetine	1
fluoxetine aurobindo	fluoxetine	1
fluoxetine 10 mg tablet	fluoxetine	1
fluoxetine merinal	fluoxetine	1
fluoxetine 20 daily	fluoxetine	1
fluoxetine syrup	fluoxetine	1
prozac (if necessary)	fluoxetine	1
fluoxetine hcl 20mg cap		1
patanol prozac	fluoxetine; olopatadine	1
ativan prozac	fluoxetine; lorazepam	1
fluoxetine capsules - 20 mg by mouth daily	fluoxetine	1
fluoxetine - 60 mg by mouth daily	fluoxetine	1
fluoxetine hcl oral tablet 10 mg		1
tylenol 8 hour fluoxetine hcl	fluoxetine hcl; tylenol 8 hour	1
fluoxetine hcl.	fluoxetine	1

fluoxetine h tab 20mg	fluoxetine	1
fluoxetine hydrochloride\olanzapine	fluoxetine hydrochloride\olanzapine	1
floxet [fluoxetine]	fluoxetine	1
fluoxentine/prozac	fluoxetine	1
fluoxetine hci 10 mg capsule	fluoxetine	1
medreich fluoxetine	fluoxetine	1
fluoxetine h 20 milligram	fluoxetine	1
prozac, 10mg daily	fluoxetine	1
fluoxetine 10mg po 4 days before menses	fluoxetine	1
fluoxan [fluoxetine hydrochloride]	fluoxetine hydrochloride	1
fluoxetine (hydrochloride)	fluoxetine	1
fluoxetine 20 mg qd	fluoxetine	1
fluoxetine injection	fluoxetine	1
fluoxetine 20	fluoxetine	1
fluoxetine(prozac)	fluoxetine	1
levalbuterol (rescue inhaler) fluoxetine	fluoxetine; levosalbutamol	1
hanall fluoxetine hcl	fluoxetine hcl	1
prozac 20 mg po qd	fluoxetine	1

Table S5. Basic Information Table.

variable	Fluoxetine Total	Sertraline Total	Escitalopram Total	Fluvoxamine Total
age_yr	39.00(22.00,56.00)	43.00(26.00,61.00)	46.00(30.00,63.00)	39.00(25.00,57.00)
age_yrQ				
<18	3005(11.82)	4102(8.02)	1382(6.40)	144(11.14)
>=18	14405(56.65)	31396(61.36)	13219(61.21)	844(65.27)
unknow	8019(31.53)	15669(30.62)	6995(32.39)	305(23.59)
Indications				
abnormal behaviour	17(0.07)	26(0.05)	12(0.05)	
anxiety disorder	138(0.53)	386(0.74)	232(1.02)	
social phobia		55(0.11)	21(0.09)	16(1.19)
stress at work		15(0.03)		
stress symptoms		10(0.02)		
attention deficit hyperactivity disorder	15(0.06)	13(0.02)		
agitated depression		10(0.02)		
attention deficit/hyperactivity disorder	20(0.08)	46(0.09)	10(0.04)	
agitation		17(0.03)	10(0.04)	
autism spectrum disorder	29(0.11)	52(0.10)	15(0.07)	

agoraphobia		19(0.04)		
back pain	11(0.04)	10(0.02)		
bipolar disorder	370(1.42)	364(0.70)	162(0.72)	17(1.27)
bipolar i disorder	81(0.31)	68(0.13)	45(0.20)	
burnout syndrome			15(0.07)	
bipolar ii disorder	14(0.05)	16(0.03)	20(0.09)	
body dysmorphic disorder	11(0.04)			
borderline personality disorder	45(0.17)	50(0.10)	23(0.10)	
asperger's disorder		14(0.03)		
adjustment disorder with depressed mood	15(0.06)	39(0.07)	31(0.14)	
acute psychosis		22(0.04)		
bulimia nervosa	43(0.17)			
chronic fatigue syndrome	12(0.05)			
completed suicide	25(0.10)	11(0.02)		
autism		11(0.02)		
decreased appetite	16(0.06)			
depressed mood	194(0.75)	480(0.92)	123(0.54)	
epilepsy			15(0.07)	
depression	7595(29.23)	15302(29.25)	7362(32.52)	274(20.45)
behaviour disorder		20(0.04)		
depression suicidal	20(0.08)	203(0.39)		
depressive symptom	66(0.25)	152(0.29)	88(0.39)	
drug abuse	68(0.26)	60(0.11)	50(0.22)	
drug exposure during pregnancy	23(0.09)	21(0.04)		17(1.27)
adverse drug reaction	22(0.08)	109(0.21)	13(0.06)	
acute stress disorder		10(0.02)		
covid-19				21(1.57)
drug use for unknown indication	194(0.75)	158(0.30)	18(0.08)	44(3.28)
dysthymic disorder	30(0.12)	40(0.08)	16(0.07)	
dementia		23(0.04)	11(0.05)	
eating disorder	38(0.15)	14(0.03)		
dementia alzheimer's type		25(0.05)		
exposure during pregnancy	21(0.08)		78(0.34)	
fatigue	12(0.05)	26(0.05)	14(0.06)	
fibromyalgia	28(0.11)	41(0.08)	22(0.10)	
foetal exposure during preg-	37(0.14)	35(0.07)		

nancy				
maternal exposure timing unspecified			72(0.32)	
generalised anxiety disorder	89(0.34)	301(0.58)	344(1.52)	
ill-defined disorder	82(0.32)	905(1.73)	35(0.15)	21(1.57)
insomnia	36(0.14)	91(0.17)	53(0.23)	
affective disorder	120(0.46)	126(0.24)	95(0.42)	
adjustment disorder		27(0.05)	21(0.09)	
intentional overdose	51(0.20)	50(0.10)	41(0.18)	
intentional self-injury	15(0.06)	41(0.08)	32(0.14)	
major depression	712(2.74)	945(1.81)	749(3.31)	19(1.42)
emotional disorder		35(0.07)		
menopausal symptoms	26(0.10)	45(0.09)	10(0.04)	
family stress		14(0.03)		
menopause	16(0.06)	18(0.03)		
mental disorder	77(0.30)	131(0.25)	76(0.34)	
feeling abnormal		21(0.04)		
migraine	29(0.11)	47(0.09)	16(0.07)	
mixed anxiety and depressive disorder	117(0.45)	209(0.40)	84(0.37)	
mood swings	24(0.09)	35(0.07)	11(0.05)	
myasthenic syndrome	25(0.10)			
grief reaction		11(0.02)		
aggression	12(0.05)	26(0.05)		
narcolepsy	11(0.04)	10(0.02)		
headache		18(0.03)	14(0.06)	
obsessive-compulsive disorder	605(2.33)	1036(1.98)	166(0.73)	226(16.87)
hot flush		16(0.03)		
others	684(2.63)	1037(1.98)	743(3.28)	167(12.46)
overdose	10(0.04)			
pain	24(0.09)	41(0.08)	27(0.12)	
panic attack	74(0.28)	349(0.67)	125(0.55)	
panic disorder	75(0.29)	250(0.48)	128(0.57)	
irritability		15(0.03)		
perinatal depression	15(0.06)	113(0.22)	18(0.08)	
irritable bowel syndrome		24(0.05)	16(0.07)	
persistent depressive disorder	18(0.07)	32(0.06)	17(0.08)	
personality disorder	27(0.10)	35(0.07)		

mania		13(0.02)		
anger	11(0.04)	14(0.03)		
adjustment disorder with mixed anxiety and depressed mood		22(0.04)		
poisoning deliberate	38(0.15)	17(0.03)	33(0.15)	
post-traumatic stress disor-der	92(0.35)	414(0.79)	80(0.35)	
postpartum depression	46(0.18)	127(0.24)	33(0.15)	
premenstrual dysphoric disorder	33(0.13)	29(0.06)	11(0.05)	
premenstrual syndrome	63(0.24)	90(0.17)	28(0.12)	
product used for unknown indication	7138(27.47)	8716(16.66)	4639(20.49)	287(21.42)
mood altered		33(0.06)		
psychiatric symptom	13(0.05)	12(0.02)	11(0.05)	
psychotic disorder	64(0.25)	75(0.14)	34(0.15)	
schizoaffective disorder	43(0.17)	61(0.12)	23(0.10)	
nervous system disorder		11(0.02)		
schizoaffective disorder bipolar type	17(0.07)			
nervousness		24(0.05)	15(0.07)	
anorexia nervosa	20(0.08)	32(0.06)		
schizophrenia	106(0.41)	113(0.22)	62(0.27)	22(1.64)
neuralgia		12(0.02)		
schizophrenia, paranoid type	14(0.05)			
seasonal affective disorder	13(0.05)	12(0.02)		
obsessive thoughts		43(0.08)	10(0.04)	
serotonin syndrome	12(0.05)			
sleep disorder	16(0.06)	50(0.10)	17(0.08)	
social anxiety disorder	36(0.14)	92(0.18)	30(0.13)	
stress	23(0.09)	141(0.27)	64(0.28)	
suicidal ideation	30(0.12)	27(0.05)	17(0.08)	
panic reaction		23(0.04)		
suicide attempt	172(0.66)	78(0.15)	91(0.40)	
parkinson's disease		10(0.02)		
tourette's disorder	13(0.05)	10(0.02)		
antidepressant therapy	135(0.52)	304(0.58)	112(0.49)	
affect lability		11(0.02)		
toxicity to various agents	22(0.08)	34(0.06)	30(0.13)	
unknown	4319(16.62)	12230(23.37)	3636(16.06)	148(11.04)

weight decreased	26(0.10)			
phobia		12(0.02)		
withdrawal syndrome	12(0.05)	10(0.02)		
postpartum anxiety		10(0.02)		
premature ejaculation		14(0.03)		
anxiety	1277(4.91)	5326(10.18)	2365(10.45)	61(4.55)
prophylaxis		12(0.02)		
psychotherapy		12(0.02)		
seizure		20(0.04)		
Reporter				
Consumer	8850(34.80)	19566(38.24)	6111(28.30)	269(20.80)
Physician	6865(27.00)	11959(23.37)	5581(25.84)	346(26.76)
Other health-professional	4034(15.86)	7830(15.30)	5246(24.29)	178(13.77)
Pharmacist	3401(13.37)	7117(13.91)	3035(14.05)	209(16.16)
unknown	1601(6.30)	3257(6.37)	1238(5.73)	286(22.12)
Lawyer	666(2.62)	1429(2.79)	351(1.63)	3(0.23)
Registered Nurse	12(0.05)	9(0.02)	34(0.16)	2(0.15)
Outcomes				
other serious	13460(44.60)	28218(51.34)	10551(44.05)	654(47.56)
hospitalization	7684(25.46)	13116(23.86)	7471(31.19)	441(32.07)
death	3325(11.02)	3598(6.55)	1550(6.47)	118(8.58)
congenital anomaly	2154(7.14)	2519(4.58)	833(3.48)	19(1.38)
life threatening	2075(6.88)	3418(6.22)	1681(7.02)	74(5.38)
disability	1325(4.39)	3783(6.88)	1244(5.19)	54(3.93)
required intervention to Prevent Permanent Impairment/Damage	157(0.52)	310(0.56)	622(2.60)	15(1.09)
Reported countries				
United States	8850(46.04)	13759(34.01)	5499(34.14)	326(40.00)
India	255(1.33)	150(0.37)	237(1.47)	
Australia	229(1.19)	237(0.59)	189(1.17)	
Japan		501(1.24)	318(1.97)	86(10.55)
Turkey	228(1.19)	322(0.80)	90(0.56)	
Spain	226(1.18)	729(1.80)	399(2.48)	
Denmark	185(0.96)	286(0.71)	1416(8.79)	
Portugal	170(0.88)	263(0.65)	150(0.93)	
China	112(0.58)	443(1.10)	123(0.76)	
Switzerland	111(0.58)	116(0.29)	137(0.85)	
Poland	93(0.48)	262(0.65)	162(1.01)	

Brazil	90(0.47)	194(0.48)		
United Kingdom	3564(18.54)	12489(30.87)	1111(6.90)	
New Zealand	72(0.37)			
Croatia		130(0.32)	75(0.47)	
Austria		118(0.29)	77(0.48)	
Belgium		86(0.21)	86(0.53)	
Czechia		85(0.21)	63(0.39)	
Norway		74(0.18)	78(0.48)	
Finland			56(0.35)	
Peru		74(0.18)		
Iran		66(0.16)		
Ireland			53(0.33)	
Venezuela		64(0.16)		
Greece		62(0.15)	56(0.35)	
France	1826(9.50)	1978(4.89)	1777(11.03)	
other	692(3.60)	933(2.31)	600(3.72)	349(42.82)
Germany	616(3.20)	2068(5.11)	1293(8.03)	
Canada	563(2.93)	732(1.81)	889(5.52)	54(6.63)
Sweden	498(2.59)	1424(3.52)	240(1.49)	
Italy	436(2.27)	1549(3.83)	776(4.82)	
Netherlands	405(2.11)	1259(3.11)	159(0.99)	
route				
other	13112(51.69)	25102(49.07)	8302(38.50)	492(38.38)
oral	9634(37.98)	22738(44.45)	12030(55.78)	762(59.44)
transplacental	2574(10.15)	3235(6.32)	1190(5.52)	28(2.18)
transmammary	25(0.10)	66(0.13)	33(0.15)	
intra-uterine	22(0.09)			
buccal		16(0.03)	10(0.05)	
sex				
female	14823(58.29)	29227(57.12)	12210(56.54)	641(49.57)
male	6938(27.28)	15869(31.01)	6825(31.60)	534(41.30)
unknown	3668(14.42)	6071(11.87)	2561(11.86)	118(9.13)
tto	12.00(0.00,99.00)	10.00(0.00,120.00)	11.00(0.00,104.00)	23.00(2.00,151.00)
ttoQ				
<7	2502(23.49)	7114(26.90)	3427(27.52)	159(23.31)
7~28	1012(9.50)	2451(9.27)	1082(8.69)	79(11.58)
28~60	505(4.74)	1359(5.14)	699(5.61)	36(5.28)
>=60	1698(15.94)	4940(18.68)	2305(18.51)	148(21.70)
unknow	4934(46.32)	10587(40.02)	4942(39.68)	260(38.12)

wt	68.94(55.00,83.91)	69.00(56.00,82.00)	69.00(56.00,82.00)	69.00(54.00,85.00)
Year				
2004	601(2.36)	998(1.95)	572(2.65)	67(5.18)
2005	734(2.89)	898(1.76)	562(2.60)	62(4.80)
2006	633(2.49)	784(1.53)	609(2.82)	23(1.78)
2007	537(2.11)	992(1.94)	619(2.87)	14(1.08)
2008	615(2.42)	786(1.54)	550(2.55)	83(6.42)
2009	755(2.97)	907(1.77)	599(2.77)	74(5.72)
2010	684(2.69)	2093(4.09)	764(3.54)	109(8.43)
2011	884(3.48)	1813(3.54)	648(3.00)	22(1.70)
2012	1197(4.71)	2192(4.28)	793(3.67)	42(3.25)
2013	1019(4.01)	2970(5.80)	821(3.80)	49(3.79)
2014	1202(4.73)	2798(5.47)	883(4.09)	29(2.24)
2015	3420(13.45)	3049(5.96)	771(3.57)	52(4.02)
2016	1238(4.87)	3187(6.23)	1143(5.29)	72(5.57)
2017	1168(4.59)	3476(6.79)	1216(5.63)	81(6.26)
2018	1556(6.12)	3625(7.08)	1895(8.77)	82(6.34)
2019	1996(7.85)	5185(10.13)	2017(9.34)	82(6.34)
2020	1530(6.02)	4375(8.55)	1742(8.07)	62(4.80)
2021	1427(5.61)	3094(6.05)	1245(5.76)	59(4.56)
2022	1445(5.68)	2941(5.75)	1400(6.48)	73(5.65)
2023	1232(4.84)	1746(3.41)	1176(5.45)	69(5.34)
2024	1121(4.41)	2216(4.33)	1153(5.34)	54(4.18)
2025	435(1.71)	1042(2.04)	418(1.94)	33(2.55)
